# Preventive Antibacterial Therapy in Acute Ischemic Stroke: A Randomized Controlled Trial

**DOI:** 10.1371/journal.pone.0002158

**Published:** 2008-05-14

**Authors:** Hendrik Harms, Konstantin Prass, Christian Meisel, Juliane Klehmet, Witold Rogge, Christoph Drenckhahn, Jos Göhler, Stefan Bereswill, Ulf Göbel, Klaus Dieter Wernecke, Tilo Wolf, Guy Arnold, Elke Halle, Hans-Dieter Volk, Ulrich Dirnagl, Andreas Meisel

**Affiliations:** 1 Department of Neurology, Charité Universitaetsmedizin Berlin, Berlin, Germany; 2 Department of Microbiology, Charité Universitaetsmedizin Berlin, Berlin, Germany; 3 Institute of Medical Immunology, Charité Universitaetsmedizin Berlin, Berlin, Germany; 4 Department of Biometrics and Clinical Epidemiology, Charité Universitaetsmedizin Berlin, Berlin, Germany; 5 Department of Neurology, Landesklinikum Waldviertel Horn, Horn, Austria; 6 Department of Neurology, Unfallkrankenhaus Berlin, Berlin, Germany; 7 Department of Neurology, Klinikum Sindelfingen-Böblingen, Sindelfingen, Germany; 8 Department of Neurology, James Cook University Hospital, Middlesbrough, United Kingdom; Stroke Unit, Hospital Clinic, Barcelona, Spain

## Abstract

**Background:**

Pneumonia is a major risk factor of death after acute stroke. In a mouse model, preventive antibacterial therapy with moxifloxacin not only prevents the development of post-stroke infections, it also reduces mortality, and improves neurological outcome significantly. In this study we investigate whether this approach is effective in stroke patients.

**Methods:**

Preventive ANtibacterial THERapy in acute Ischemic Stroke (PANTHERIS) is a randomized, double-blind, placebo-controlled trial in 80 patients with severe, non-lacunar, ischemic stroke (NIHSS>11) in the middle cerebral artery (MCA) territory. Patients received either intravenous moxifloxacin (400 mg daily) or placebo for 5 days starting within 36 hours after stroke onset. Primary endpoint was infection within 11 days. Secondary endpoints included neurological outcome, survival, development of stroke-induced immunodepression, and induction of bacterial resistance.

**Findings:**

On intention-to treat analysis (79 patients), the infection rate at day 11 in the moxifloxacin treated group was 15.4% compared to 32.5% in the placebo treated group (p = 0.114). On per protocol analysis (n = 66), moxifloxacin significantly reduced infection rate from 41.9% to 17.1% (p = 0.032). Stroke associated infections were associated with a lower survival rate. In this study, neurological outcome and survival were not significantly influenced by treatment with moxifloxacin. Frequency of fluoroquinolone resistance in both treatment groups did not differ. On logistic regression analysis, treatment arm as well as the interaction between treatment arm and monocytic HLA-DR expression (a marker for immunodepression) at day 1 after stroke onset was independently and highly predictive for post-stroke infections.

**Interpretation:**

PANTHERIS suggests that preventive administration of moxifloxacin is superior in reducing infections after severe non-lacunar ischemic stroke compared to placebo. In addition, the results emphasize the pivotal role of immunodepression in developing post-stroke infections.

**Trial Registration:**

Controlled-Trials.com ISRCTN74386719

## Introduction

The prognosis of stroke mainly depends on the incidence of complications [1], [2]. Stroke-associated pneumonia, occurring in 7 to 22% [3], is one of the main severe complications [4], [5] and thought to be the most common cause of poor outcome and death in stroke patients [6]–[8]. The risk for pneumonia is highest in the acute state of stroke [9] and in patients with non-lacunar strokes in the MCA territory [7]. Several risk factors contribute to the increased susceptibility of stroke patients for infections: aspiration due to drowsiness, impaired bulbar reflexes, dysphagia, and hypostasis in bed-ridden patient's, as well as the need for invasive medical procedures [10]. Recently, we demonstrated in a mouse stroke model that infections and mortality can effectively be reduced, and neurological outcome improved by preventive antibacterial therapy with the fluoroquinolone antibiotic moxifloxacin [11]. Based on these findings we designed the PANTHERIS trial to investigate whether preventive antibacterial short-term therapy (PAT) reduces the incidence of infections compared to the current standard therapy. In an explorative fashion we tested whether PAT also reduces mortality and improves neurological outcome. To evaluate the safety of the proposed preventive regimen of treatment we tested whether PAT promotes resistance among facultative pathogenic bacteria. Finally, we seek for underlying immunological mechanisms of increased infectious susceptibility after stroke.

## Methods

The protocol for this trial and supporting CONSORT checklist are available as supporting information; see Checklist S1 and Protocol S1.

This investigator initiated study was designed as a randomized, double-blind, placebo-controlled trial in two academic centers (Charité Campus Mitte, Charité Campus Benjamin Franklin) and one community hospital (Unfallkrankenhaus Berlin), and was conducted between May 2003 and July 2006. The protocol was approved by the local ethics committee. Informed consent was obtained from the patient or their legal guardian. PANTHERIS was registered with ISRCTN74386719 at Current Controlled Trials (http://www.controlled-trials.com/ISRCTN74386719). Data safety was independently monitored and audited by local monitoring authorities (Koordinationszentrum für klinische Studien, KKS Charité). Study entry criteria were the occurrence of an acute ischemic stroke (between 9 and 36 h after onset) in the MCA territory with a score of at least 12 on the National Institute of Health Stroke Scale (NIHSS), and patient age of at least 18 years. Exclusion criteria were: hemorrhagic stroke, clinical signs of infection on admission, contraindications against moxifloxacin, preceding or ongoing antibiotic therapy within the last 24 h, participation in another interventional trial, or immunosuppressant treatment within the last 30 days. In order to avoid inclusion of lacunar strokes, only patients with signs of cortical involvement (e.g. aphasia, neglect) or with disturbances of consciousness in addition to hemiparesis were included if initial CT scan was negative for signs of ischemic stroke. We used a computer generated allocation schedule. Because of the greater number of strata, an adaptive randomization was adopted, including sex, affected MCA territory, age (≤64 vs. >64), and centre as stratifying factors [12]. Trial pharmacists in each site labelled the trial drugs with sequential study numbers according to randomisation lists prepared by the trial statistician and dispensed the drugs. Study investigators and enrolling stuff were masked to the assignment.

Patients were randomly assigned to receive a daily iv infusion over 60 minutes either verum [n = 40, moxifloxacin 400 mg/250 mL solution for infusion (Avalox®, Bayer Vital GmbH)] or placebo [n = 40, riboflavin as a colorant, 20 mg/250 mL NaCl 0.9%] for five days starting within 36h after stroke onset. Verum and placebo medication were visually indistinguishable. In case of an intercurrent infection warranting treatment, in both groups the study medication was continued and supplemented by a treatment following predefined schemes: Treatment of 1) pneumonia: 3×1 g/d iv ceftazidime and 1×5 mg/kg bw/d iv tobramycin; 2) endocarditis/bacteriemia: 2×1g/d iv vancomycin 3) urinary tract infections: 2×400 mg iv ciprofloxacin. However, when infections were refractory to therapy, study medication could be discontinued, and treatment could be adapted following medical reasoning.

After admission all patients underwent cranial CT scanning to exclude intracranial hemorrhage. Patients were treated on stroke units according to recommendations of the European stroke initiative [13]. C-reactive protein was measured daily. Neurological diagnostic work up aimed at identification of stroke cause, which was classified following TOAST criteria [14]. Comorbidity factors were recorded. Neurological deficit was measured at admission and on days 2, 6, and 11 using NIHSS [15].

Primary endpoint was the infection rate within 11 days after stroke onset. The day when infection occurred refers to the day after stroke onset and not study treatment initiation. Infections were diagnosed according to modified criteria of the U.S. Centers for Disease Control and Prevention (CDC): Pneumonia was diagnosed when at least one of the first and one of the latter criteria were fulfilled: A) abnormal respiratory examination, pulmonary infiltrates in chest x-rays; B) productive cough with purulent sputum, microbiological cultures from lower respiratory tract or blood cultures, leukocytosis, and elevation of C-reactive protein (CRP). Diagnosis of urinary tract infection (UTI) was based on two of the following criteria: fever (>38·0°C), urine sample positive for nitrite, leucocyturia, and significant bacteriuria. Secondary endpoint measures were survival and functional outcome (Barthel Index [16]; BI) at day 180 after stroke. BI was dichotomized into good (BI≥60) and bad outcomes (BI<60).

Quantitative or semiquantitative cultures from lower respiratory tract (sputum, tracheobronchial or bronchoalveolar lavage), urine, and blood cultures (BacT/Alert FA aerobic and anaerobic bottles, Biomerieux, INC. Durham, NC 27704, USA) were performed in each case of infection. Urine samples were obtained from previously inserted urinary catheters. Significant bacteriuria was considered when >10^4^ cfu/mL of a uropathogen were isolated. Quantitative cultures of bronchoalveolar lavage or tracheobronchial samples have used a diagnostic threshold of >10^4^ cfu/mL and of >10^5^ cfu/mL, respectively. Species identification and antimicrobial susceptibility testing (microbroth dilution test) followed standard operating procedures according to Clinical and Laboratory Standards Institute (CLSI). For isolation, identification and susceptibility testing of *E. coli* isolates before (day 1) and after treatment (day 9) with moxifloxacin or placebo, stool samples of each patient were cultured on TBX-Chromogen-Agar (Tryptone Bile-X-Glucoronic Agar-SIFIN GmbH, Berlin, Germany). After overnight incubation at 37°C, ten individual putative *E. coli* colonies were isolated. Following additional biochemical identification with VITEK2/GN-cards (Biomerieux, INC. Durham, NC 27704, USA) minimal inhibitory concentrations (MICs) of all *E. coli* isolates were determined by microdilution testing according to CLSI standards for ciprofloxacin (MIC ≤1 mg/L susceptible, MIC ≥4 mg/L resistant) and according to the European Committee on Antimicrobial Susceptibility Testing for moxifloxacin (MIC ≤0.5 mg/L susceptible, ≥2 mg/L resistant). *E. coli* isolates were further investigated for the presence of known mutations in the quinolone resistance determining region (QRDR) of the DNA gyrase subunit A gene *gyr*A. The *gyr*A (Ser-83) mutation was detected in isolated DNA by the mismatch amplification mutation assay (MAMA) as described earlier [17]. Expression of human leukocyte antigen-DR (HLA-DR) on monocytes was determined by flow cytometry using a highly standardized quantitative assay as described earlier [18]. Briefly, on days 1, 3, 8, 90, and 180 50 µl of EDTA-blood was stained with 20 µl of monoclonal phycoerythrin-conjugated anti-human leukocyte antigen-DR (HLA-DR) antibodies and peridin chlorophyll (PerCP-Cy5.5)-conjugated anti-CD14-antibodies (QuantiBrite™, Becton Dickinson) for 30 min in the dark at room temperature. For lysis of erythrocytes, samples were incubated with 500 µl FACS Lysing solution (Becton Dickinson) for 15 min in the dark at room temperature. Subsequently, cells were washed with 1 ml of FACS buffer and analyzed on a FACS Calibur flowcytometer using CellQuest software after QuantiBrite calibration for 1∶1 quantification. Final analysis was performed using Quanticalc software (all from Becton Dickinson) to obtain the molecules HLA-DR per cell from the measured geometric means. The inter-assay CV was <5% and the inter-lab CV of this assay was <20% [19].

### Statistical analysis

Based on a retrospective analysis of infection rates in patients with acute ischemic stroke in the MCA territory treated in the participating centers (data not shown), an infection rate of 40% in the placebo group, and 10% in the group treated with moxifloxacin was assumed. A priori power analysis revealed that a sample size of n = 32 per group was needed (two-sided α = 5%, power 80%). With an estimated drop out rate of 20%, 40 patients per group were needed for enrolment. After enrolment of the last patient a blinded review meeting assessed study patients with respect to protocol violations, which were labelled as “invalid” for per protocol analysis. The efficacy of preventive antibacterial therapy in reducing the incidence of post-stroke infections was investigated using the *per protocol* population as the main analysis set instead of the *intention to treat* approach in this phase IIb trial.

Continuous variables were described using arithmetic mean, standard deviation, and 95% confidence interval (CI) for normally distributed data, or median and range for non-normally distributed data, respectively. Normal distribution was checked by Q-Q-Plots and tested using the modified Kolmogorov-Smirnov test [20]. Absolute and relative frequencies were used for dichotomous variables. The infection rates (and other proportions) of placebo and verum group were compared using Fisher's exact test and the Cochran-Mantel-Haenszel-test, stratified for study centers. For analysis of CRP levels and daily maximum body temperature the multivariate repeated measures analysis of variance was done as described previously [21]. Differences in CRP values between treatment groups were analyzed based on a mixed model of logarithmically transformed data. The parameters day and treatment are assumed fixed, the factor patient is assumed random. Measurements between patients are assumed independent distributed. Within patients a common, otherwise arbitrary covariance matrix is assumed. The following models were used. For the “no interaction model“: log(CRP_ij,p_) = μ+α_i_+τ_j_+p(τ_j_)+ε_ij_., for the “interaction model”: log(CRP_ij,p_) = μ+α_i_+τ_j_+γ_ij_+p(τ_j_)+ε_ij,_ where μ is the grand mean, α_i_ the day (i = 1, 2, 3, 4, 5, 6, 7, 9, 11), τ_j_ the treatment (j = 1 for verum, 2 for placebo), p(τ_j_) the patient nested within treatment, γ_ij_ the interaction treatment by day, and ε_ij_ the error term. In order to analyse the impact of risk factors for stroke-associated infection multivariately, we used a multivariate logistic regression analysis, including the calculation of odds ratios and the corresponding 95% confidence intervals. Feature selection (in reduction steps) was applied to show the most important influencing (independent) factors. The clinical characteristics of both treatment groups and of patients without and with infection were compared in univariate analyses by Student's t test for continuous variables with a normal distribution. For non-normal data and categorical variables the Mann-Whitney U test and the Chi-Square test for few categories were used, respectively. Survival in treatment and infection groups was calculated according to Kaplan-Meier and compared univariately with Log-Rank-statistics. Multiple tests for differences between the groups in question have been regarded as exploratory ones and were not adjusted for multiplicity. A two-tailed p<0.05 was considered statistically significant. The statistical analysis used the Software Package for Social Sciences, SPSS for Windows, 13.0, SPSS, Inc., Chicago, IL and SAS, Version 9.1, by SAS Institute, Inc., Cary, NC, USA.

## Results

### Study population

**Figure 1:**
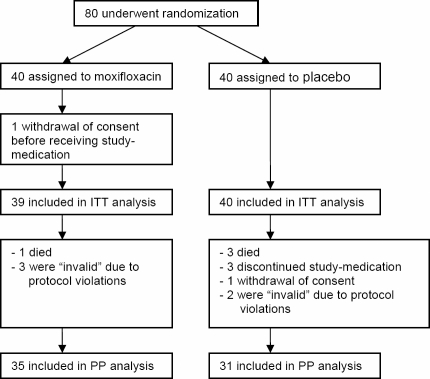
PANTHERIS trial profile.

Eighty patients with acute ischemic stroke were randomized. For one patient informed consent was withdrawn before receiving study medication. Therefore, 79 patients were included into intention-to-treat (ITT) analysis (verum n = 39; placebo n = 40). Baseline characteristics were similarly matched in both treatment groups (Table 1), except for coronary heart disease which occurred more frequent in the verum group (p = 0.05). 66 patients (verum n = 35; placebo n = 31) were analysed *per protocol* (PP). Eight patients, who neither reached the primary endpoint nor completed the study protocol until day 11 had to be excluded from the PP analysis: Four patients (1 verum, 3 placebo) died within 3 days. In three patients, study medication was discontinued due to medical reasons (withdrawal of life supporting therapy, hemicraniectomy, blinding opened due to a suspected CNS infection as the cause of the neurological deficits instead of ischemic stroke). In one patient informed consent was withdrawn. Five additional patients (3 verum, 2 placebo) were retrospectively excluded due to protocol violations (e.g. allocated in infection group while failing predefined infection criteria) and considered “invalid” in the blinded reviewing process (Figure 1).

**Table 1:** Baseline characteristics

	Placebo (n = 40)		Verum (n = 39)		p
	n	%	n	%	
Centre 1	25	62.5	22	56.4	0.65
Centre 2	11	27.5	10	25.6	1.00
Centre 3	4	10.0	7	18.0	0.35
Male/female	14/26	35.0/65.0	15/24	38.5/61.5	0.82
Right/left MCA	18/22	45.0/55.0	20/19	51.3/48.7	0.66
Mean age (years) (95%CI)	72·7	68.6–76.9	72.4	68.8–76.1	0.80
NIHSS ad admission (Median,min-max)	15	12–25	17	12–21	0.66
**Qualifying event**					
*Cardioembolism*	19	47.5	16	41.0	0.65
*Atherothrombotic*	12	30.0	14	35.9	0.64
*Small-vessel*	2	5.0	1	2.6	1.00
*Other*	2	5.0	3	7.7	0.66
*Unknown*	5	12.5	5	12.8	1.00
**Risk factors**					
*Hypertension*	24	60.0	27	69.2	0.48
*Diabetes mellitus*	13	32.5	12	30.8	1.00
*Current smoking*	6	15·0	3	7.7	0.48
*Hypercholesterolaemia*	6	15.0	5	12.8	1.00
*Atrial fibrillation*	16	40.0	11	28.2	0.34
*Malignancy*	4	10.0	3	7.7	1.00
*Coronary heart disease*	7	17.5	15	38.5	0.05
*Peripheral arterial disease*	1	2.5	2	5.1	0.62
*COPD*	3	7.5	3	7.7	1.00
*Prior stroke*	5	12.5	4	10.3	1.00
**Gastric tube feeding**	27	67.5	25	64.1	0.82
**Mechanical ventilation**	6	15.0	1	2.5	0.11
**Urinary catheters**	37	92.5	39	100	0.24
**Time delay to first dose [h] (mean, 95%CI)**	24.0	20.3–27.7	24.0	20.8–27.2	0.70
**Baseline parameters**	mean	95%CI of mean	mean	95%CI of mean	
Heart rate [beats/min]	81.7	76.2–87.2	77.6	72.4–82.9	0.24
Body temperature [°C]	37.0	36.9–37.2	37.0	36.8–37.2	0.69
Breathing rate, in patients breathing spontaneously [breaths/min]	21.2	18.9–23.5	18.7	16.9–20.6	0.07
Systolic BP [mmHg]	161.6	154.3–168.8	159.0	150.8–167.2	0.55
Diastolic BP[mmHg]	76.8	71.0–82.6	74.5	70.0–79.0	0.54
Glucose [mg/dl]	146.4	130.9–162.0	144.3	129.2–159.3	0.88
C-reactive protein [mg/dl]	1.4	0.8–2.0	0.9	0.5–1.3	0.41
WBC×10^9^/L	9.3	8.3–10.4	9.0	8.0–10.1	0.70

MCA: middle cerebral artery; NIHSS: National Institute of Health Stroke Scale; COPD: chronic obstructive pulmonary disease; BP: blood pressure, WBC: white blood cells.

### Primary endpoint: Prevention of post-stroke infections

In the ITT population (n = 79; Table 2A), 19 patients (24.1%) developed infections within 11 days after stroke onset. Six patients (15.4%) treated with moxifloxacin developed an infection as compared with 13 patients (32.5%) in the placebo group (17.1% reduction, Fisher exact: p = 0.114; Mantel-Haenszel-test stratified for centres: p = 0.068). In PP population (n = 66) infection rate with n = 6 in the moxifloxacin group (17.1%) was significantly lower (Fisher exact: p = 0.032; Mantel-Haenszel: p = 0.033) compared to the placebo group with 13 patients (41.9%; Table 2B). The reduction of 24.8% (95% CI 2.8%–44.3%) approximates to a number needed to treat (NNT) of 4 (95% CI 2.3–35.3). Treatment with moxifloxacin led to a relative risk reduction of 46.4% in ITT and 59.1% in PP analyses, respectively. A difference in infection rate between both groups emerged between day 2 and 4 and was maintained until day 11 after stroke onset (data not shown). In either population 11 patients suffered from pneumonia (3 verum, 8 placebo) and 8 patients had a urinary tract infection (3 verum, 5 placebo). On average, pneumonia was diagnosed 4.7 days (±2.5) after stroke, and urinary tract infection after 5.2 days (±2.9). In the ITT population the incidence of pneumonia was 8/40 (20.0%) of the placebo group compared with 3/39 (7.7%) of the verum group (12.3% reduction, p = 0.193). In the PP population 8/31 (25.8%) of placebo treated patients suffered from pneumonia, compared to 3/35 (8.6%) of the verum group (reduction 17.2%; p = 0.097). The incidence for urinary tract infections was similar for both treatment groups [ITT: 3/39 (7.7%) verum *vs.* 5/40 (12.5%) placebo; p = 0.712; PP: 3/35 (8.6%) verum *vs.* 5/31 (16.1%) placebo; p = 0.459).

**Table 2:** Incidence of infections in ITT and PP population

Treatment arm	No infection n [%]	infection n [%]	Sum n
ITT population			
Placebo	27 [67.5]	13 [32.5]	40
Verum	33 [84.6]	6 [15.4]	39
Sum	60 [75.9]	19 [24.1]	79
PP population			
Placebo	18 [58.1]	13 [41.9]	31
Verum	29 [43.9]	6 [17.1]	35
Sum	47 [71.2]	19 [28.8]	66

The numbers and rates [in brackets] of infections in both treatment groups are shown for ITT and PP populations, respectively.

### Bacterial spectrum

Microbiological analysis of microorganisms isolated from patients in the placebo group with infections identified a typical bacterial spectrum of early onset nosocomial pneumonia or urinary tract infection (Table 3). The identification of relevant pathogens was successful in 5 of 11 lower respiratory tract infections and in 4 of 8 urinary tract infections. In the verum group we identified one patient with post-stroke pneumonia caused by methicillin-resistant *Staphylococcus aureus* (MRSA). Analyzing microbiological isolates obtained from the same patient before starting study medication revealed MRSA colonization.

**Table 3:** Bacterial pathogens isolated from stroke patients specified for pneumonia and urinary tract infection

Pneumonia (n = 11)	Placebo (n = 8)	Verum (n = 3)
*S. aureus* (methicillin sensitive)	3	
*S. aureus* (methicillin resistant)		1
*S. pneumoniae*	1	
no pathogen	2	2
Not done	2	
**Urinary tract infection (n = 8)**	Placebo (n = 5)	Verum (n = 3)
*E. coli*	1	1
*E. coli+Enterococcus faecalis*	1	
*Proteus mirabilis*	1	
no pathogen	2	2

### Analysis of moxifloxacin resistance

We investigated ciprofloxacin and moxifloxacin susceptibility in *E. coli* isolated from stool samples before and after treatment. Before treatment, microbiological analysis of stool samples from 59 patients revealed that 40 patients, 20 in each treatment group, were colonized with *E. coli* (Table 4). Susceptibility testing showed that isolates from 39 patients were susceptible to ciprofloxacin and moxifloxacin. One patient in the placebo group carried a mixed culture of susceptible and resistant *E. coli*. Three days after the end of the treatment period (day 9), stool samples of the same 20 patients of each study group revealed that *E. coli* could be re-isolated from 3 patients of verum group (15%) and from 14 patients of placebo group (70%). Resistant isolates were detected in the one patient from the placebo group, who was already initially colonized by resistant *E. coli* (see above) and in one additional patient in the verum group. Genetic analysis by MAMA assay demonstrated that the *gyr*A (Ser-83) mutation was present in resistant *E. coli* isolates but absent in all other susceptible isolates. This indicates that fluoroquinolone resistance in both patients was caused by known molecular changes in the DNA gyrase.

**Table 4:** Susceptibility analysis of *E. coli* isolates

	*E. coli* isolates before treatment		*E. coli* isolates after treatment	
Treatment arm	Sensitive# n	Resistant## n	Sensitive# n	Resistant## n
Placebo	20	1*	13	1*
Verum	20	0	2	1

# ciprofloxacin MIC ≤1 mg/L, moxifloxacin MIC ≤0.5 mg/L;

## ciprofloxacin MIC ≥4 mg/L, moxifloxacin MIC ≥2 mg/L;

* Isolated from a patient carrying a mixture of resistant and sensitive *E. coli* isolates

### Mechanical ventilation

At study entry no patient was under mechanical ventilation (MV). Within 11 days, seven patients (1 verum, 6 placebo) in the ITT population (Table 1) and five patients (1 verum, 4 placebo) in the PP population underwent MV. Frequencies of MV were not significantly different between both treatment groups (Table 1). Median time point for beginning of MV was at day 3 (range 1–8). The MV of the verum-treated patient started at day 2. This patient developed pneumonia on day five. From the 6 patients of the placebo group which underwent MV (median day 3; range: 1–8) two underwent intubation with clinical signs of pneumonia, and in one patient MV was necessary four days after onset of pneumonia (median day 3, range: 2–4). From the 7 patients undergoing MV two (both from placebo group) died within one month (day 22 and 30).

### Daily maximum body temperature and C-reactive protein

**Figure 2:**
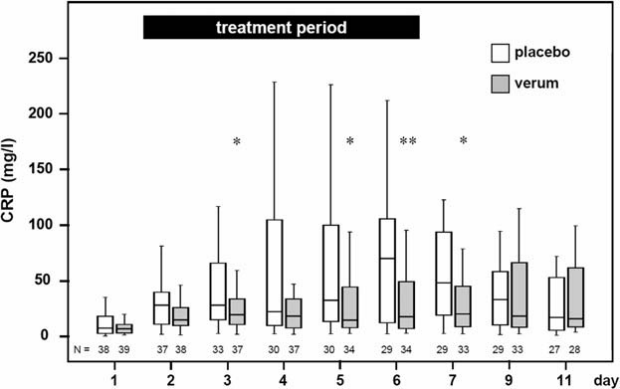
Time course of CRP serum concentration. Time course of CRP levels in placebo and verum group shown by boxplots. Number of patients is indicated by N. CRP levels in the moxifloxacin group are significant lower compared to the placebo group. The p-values for the main effects are <0.0001 for day of CRP measurement and 0.0186 for treatment (multivariate analysis of variance for repeated measurements on logarithmically transformed data, no interaction model). The dependency between treatment and day of CRP measurement has a p-value of 0.0845 (interaction model). In patients of the verum group CRP concentration are significantly lower compared to patients of the placebo group 3, 5, 6, and 7 days after stroke (* *p<*0.05; ** *p*<0.005).

Patient maximum body temperature per day did not differ significantly between both treatment groups (ITT population; data not shown). CRP levels from patients (ITT population) of the placebo group increased significantly more than in the verum group (p = 0,0186; Figure 2). Comparing CRP levels univariately, a significantly lower CRP concentration was found in the verum group at days 3, 5, 6 and 7, which followed the treatment period with an interval of one day (Figure 2).

### Survival

**Figure 3:**
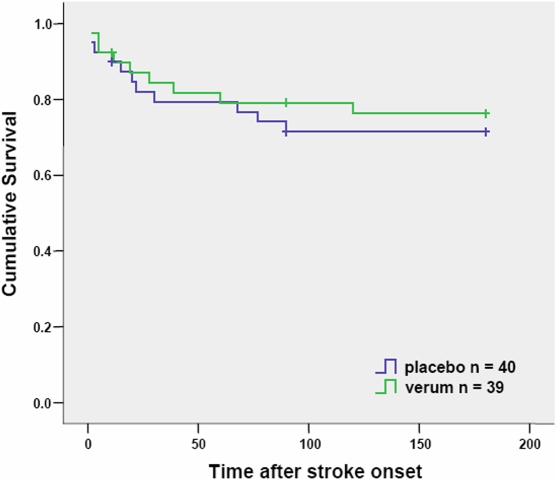
Kaplan-Meier curves of survival in both treatment groups. Crosses indicate time points when patients were lost to follow-up (‘censored’; n = 7; verum n = 3, placebo n = 4 placebo). Seven (verum n = 3, placebo n = 4) of 79 patients (8.9%) died within 11 days after stroke onset. Within 6 month after stroke 13 (verum n = 6, placebo n = 7) of 72 patients (18.1%) died.

**Figure 4:**
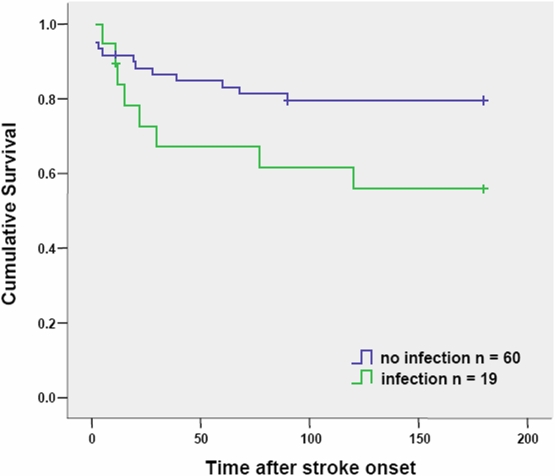
Kaplan-Meier curves of survival in patients with and without infections. Crosses indicate time points when patients were lost to follow-up (‘censored’; n = 7, infection n = 1, no infection n = 6). Seven (no infection n = 5, infection n = 2) of 79 patients (8.9%) died within 11 days after stroke onset. Within 6 month after stroke 13 (infection n = 6, no infection n = 7) of 72 patients (18.1%) died.

To avoid a preselection bias, cumulative survival analyses are based on ITT and not PP population, since patients who died within the first 11 days after stroke without reaching the primary endpoint were considered as ‘drop outs’ for PP population. Seven (3 verum, 4 placebo) of 79 patients (8.9%) died within 11 days after stroke onset. Within 6 month after stroke 13 (6 verum, 7 placebo) of 72 patients (18.1%) died, and 7 patients were lost in follow-up (3 verum, 4 placebo). Survival did not differ significantly between placebo and verum group 180 days after stroke onset (Log Rank p = 0.618, Figure 3). However, cumulative survival of patients with infection compared to patients without infection was significantly different (p<0.001) during follow-up period (Figure 4). Patients with stroke-associated pneumonia had a significantly lower (p = 0.022) survival rate in follow-up compared with patients without infections whereas patients with urinary tract infections showed no differences in cumulative survival compared with patients without infections (p = 0.898, data not shown).

### Neurological Outcome

Neurological outcome 6 month after stroke onset was determined by using the Barthel Index (BI) as a measure of limitations in activities of daily living. Comparing both treatment arms after dichotomization, in the ITT population the rate of patients with good outcome (BI≥60) was 64% in the verum group (14 out of 22) compared to 55% in the placebo group (11 out of 20) (p = 0.574). In the PP population the rate of patients with good outcome was 67% in the verum group (14 out of 21) compared to 55% in the placebo group (10 out of 18) (p = 0.483). In ITT as well as PP population, both treatment groups displayed the same median 70 (p = 0.700; p = 0.543). In the ITT population the rate of patients with good outcome was 61% in the non-infection group (20 out of 33 patients) compared to 63% in the infection group (5 out of 8, p = 0.922). In the PP population the rate of patients with a BI≥60 was 61% in the non-infection group (19 out of 31 patients) compared to 63% in the infection group (5 out of 8, p = 0.951).

### Levels of monocytic HLA-DR expression

**Figure 5:**
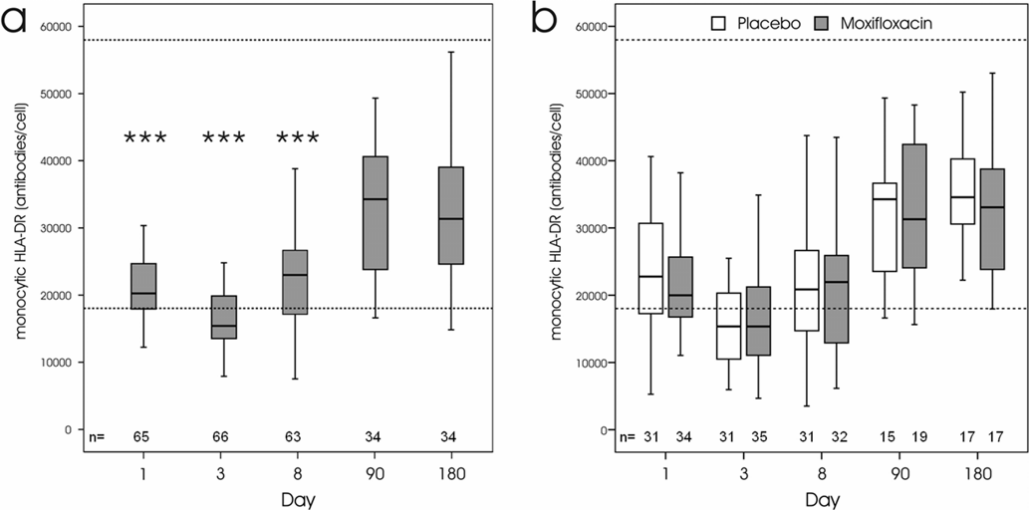
Time course in monocytic HLA-DR expression after stroke. a: Monocytic HLA-DR expression is significantly reduced in all patients at days 1, 3, and 8 compared to 90 and 180 days after stroke (*** *p*<0.001). b: The time course of monocytic HLA-DR expression is not significantly different between patients from the placebo and verum groups. Dashed lines indicate the upper and lower reference range for monocytic HLA-DR levels in healthy individuals (5% percentile = 18036 mAb/cell; 95% percentile = 57958 mAb/cell).

**Figure 6:**
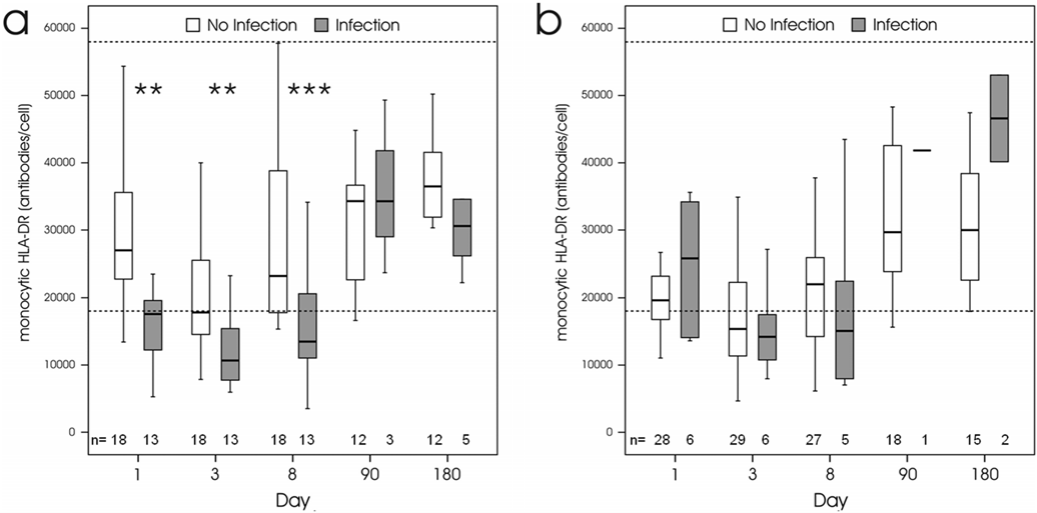
Monocytic HLA-DR expression in patients with and without infections. a: In the placebo group, monocytic HLA-DR expression in patients with infections is significantly reduced compared to patients without infections at day 1 (** *p* = 0.003), 3 (** *p* = 0.005), and 8 (*** *p*<0.001) days, but not at day 180 after stroke. b: In the verum group, monocytic HLA-DR expression is not significantly different between patients with and without infections. Dashed lines indicate the upper and lower reference range for monocytic HLA-DR levels in healthy individuals (5% percentile = 18036 mAb/cell; 95% percentile = 57958 mAb/cell).

To determine the impact of cerebral ischemia on immune competence in relation to the risk of post-stroke infections we used a novel standardized assay to quantify the expression of monocytic HLA-DR. All patients (PP population) showed signs of post-stroke immunodepression, as indicated by significantly reduced monocytic HLA-DR expression at days 1, 3, and 8 compared to 3 and 6 months after stroke (Figure 5a). There were no significant differences in the time course of monocytic HLA-DR levels between the placebo and verum group (Figure 5b). However, infected patients in the placebo group had significantly lower monocytic HLA-DR levels at days 1, 3, and 8, as shown in Figure 6a. In contrast, no significant differences in monocytic HLA-DR expression were observed between infected and non-infected patients in the verum group at all time points (Figure 6b). Notably, patients without infections in the verum group had significantly lower monocytic HLA-DR levels at day 1 compared to patients without infections in placebo group corroborating that moxifloxacin treatment effectively prevented infections in a proportion of these patients. At 6 month after stroke, monocytic HLA-DR expression returned to normal levels and was not signifcantly different between surviving patients with and without infections in both groups (Figure 6a, b).

### Risk factors of post-stroke infections

Infection rate within 11 days was significantly associated with tube feeding, mechanical ventilation, treatment, and monocytic HLA-DR levels at baseline (day 1 after stroke onset and before study treatment onset) in univariate PP analysis of the placebo group (Table 5). Comparing patients with and without infections of both treatment arms in the PP population (data not shown) only tube feeding was significantly more frequent in infected patients (p = 0.001). We performed regression analysis in PP population adjusted for NIHSS at admission, age, tube feeding, mechanical ventilation, monocytic HLA-DR expression at day 1 post stroke and treatment arm, which revealed that treatment arm and the interaction between treatment arm and HLA-DR expression were the strongest independent predictors for post-stroke infections (Table 6).

**Table 5:** Univariate analysis for predictors of post-stroke infections in the placebo arm of PP population.

	Infection (N = 13)		No Infection (N = 18)		P
	n	%	n	%	
male:female	6/7	46.2/53.8	6/12	33.3/66.7	0.710
Right:left MCA	4/9	30.8/69.2	8/10	44.4/55.6	0.484
Age (95%CI)	75.1	68.5–81.6	70.3	64.9–75.7	0.154
NIHSS ad admission (Median,min-max)	17	13–25	14.5	12–20	0.080
NIHSS 180 days. (Median,min-max)	11.5	4–16	5	1–14	0.108
**Qualifying event**					
*Cardioembolism*	8	61.5	7	38.9	0.285
*Atherothrombotic*	5	38.5	6	33.3	1.000
*Small-vessel*	0	0.0	1	5.6	1.000
*Other*	0	0.0	2	11.1	0.497
*Unknown*	0	0.0	2	11.1	0.497
**Risik factors**					
*Hypertension*	7	53.8	13	72.2	0.449
*Diabetes mellitus*	4	30.8	7	38.9	0.718
*Current smoking*	3	23.1	2	11.1	0.625
*Hypercholesterinemia*	3	23.1	2	11.1	0.625
*Atrial fibrillation*	5	38.5	7	38.9	1.000
*Malignoma*	1	7.7	2	11.1	1.000
*Coronary heart disease*	2	15.4	2	11.1	1.000
*Peripheral arterial disease*	0	0.0	0	0.0	NA
*COPD*	2	15.4	0	0.0	0.168
*Prior Stroke*	1	7.7	3	16.7	0.621
**Tube feeding**	11	84.60	8	44.40	0.032
**Urinary catheters**	13	100.00	15	83.30	0.245
**Mechanical ventilation**	4	30.80	0	0.00	0.023
**Delay to first dose h (mean, 95%CI)**	28.6	19.9–37.3	20.9	16.3–25.5	0.173
**Baseline parameters**	mean	95%CI of mean	mean	95%CI of mean	
Heart beats [/min]	68.5	75.0–97.9	78.2	70.8–85.6	0.346
Temperature [°C]	37.1	36.9–37.4	36.9	36.6–37.1	0.097
Breathing frequency [/min]	19.3	13.8–24.9	22.4	18.4–26.4	0.472
Systolic BP [mmHg]	166.3	151.1–181.5	159.5	147.4–171.6	0.446
Diastolic BP[mmHg]	82.1	74.4–89.7	76.9	67.8–86.0	0.326
Glucose [mg/dl]	143.6	124.6–162.7	160.3	128.1–192.4	0.781
C-reactive protein [mg/dl]	0.988	0.317–1.659	1.070	0.420–1.720	0.737
WBC×10^9^/L	9.0	7.6–10.4	8.6	7.4–9.8	0.594
mHLA-DR [antib./cell]	17273	12197–22349	28541	23521–33561	0.003

Tube feeding, mechanical ventilation, and expression of mHLA-DR were associated univariately with post-stroke infection.

**Table 6:** Logistic regression analysis of independent predictors for post-stroke infections

Effect	Parameter estimation			Standard	p-value
	Estimate	95% Confidence Interval		error	
		Lower	Upper		
Model					
Treatment					0.015
Interaction: treatment and HLA-DR					0.036
Estimates					
Intercept	−12.7	−38.4	13.1	13.1	0.336
Treatment	47.2	9.0879	85.4014	19.5	0.015
HLA-DR level in verum arm	1.113	−1.4563	3.6827	1.311	0.396
HLA-DR level in placebo arm	−3.507	−6.3319	−0.6822	1.441	0.015

Shown are the nominal p-values and estimates for the parameters remaining in the model after backward selection. Factors and covariates included for selection were: NIHSS at admission, age, tube feeding, mechanical ventilation, HLA-DR expression (Day 1), treatment, and interaction between treatment and HLA-DR (Day 1) expression. The final logistic model was: log( p/1-p) = μ+treatment+ß_treatment_^*^log(HLA-DR); p: number of infected patients within 11 days/total number of patients (in the respective treatment group).

### Adverse events

Table 7 shows types and frequencies of adverse events (AE) in ITT population reported over the whole study period including 6 month follow-up. There were no serious adverse events other than the above reported 19 cases of infections and 12 deaths of patients. Overall 40 cardiovascular, neurological, gastrointestinal, laboratory, and general medical AE were reported. Concerning the frequency of AE both treatment groups did not differ. There was no report of AE related to study medication.

**Table 7:** Safety analysis

	Placebo (n = 40)		Verum (n = 39)		
	n	%	n	%	p
1 AE	14	35	9	22.5	0.32
≥2 AE	2	5	5	12.5	0.43
*Pulmonary*	3	7.5	0	0	0.24
Dyspnoea	2	5	0	0	
Pneumothorax	1	2.5	0	0	
*Neurological*	4	10	8	20.5	0.23
Increased intracranial pressure	2	5	2	5.1	
Hemorrhagic transformation of stroke	1	2.5	2	5.1	
Seizures	1	2.5	1	2.6	
Recurrent stroke	0	0	1	2.6	
Frontal lobe syndrome	0	0	1	2.6	
Hallucination	0	0	1	2.6	
*Cardiac arrhythmia*	1	2.5	1	2.6	1
Ventricular tachycardia	1	2.5	0	0	
Sinus bradycardia	0	0	1	2.6	
*Gastrointestinal*	4	10	3	7.7	1
Abdominal pain	1	2.5	0	0	
Diarrhea	2	5	2	5.1	
Nausea/Vomitting	1	2.5	1	2.6	
*Laboratory changes*	2	5	4	10.3	0.43
Hypothyreosis	1	2.5	0	0	
Hyponatriaemia	1	2.5	0	0	
Alanine aminotransferase increased	0	0	1	2.6	
Blood creatine phosphokinase increased	0	0	1	2.6	
Haemoglobin decreased	0	0	1	2.6	
Hypocalcaemia	0	0	1	2.6	
*Other*	1	2.5	3	7.7	0.36
Arthralgia	0	0	2	5.1	
Hemolysis	0	0	1	2.6	
Pharyngeal bleeding	1	2.5	0	0	

## Discussion

PANTHERIS, is an investigator-initiated, multi-center, randomised, double-blind, placebo-controlled trial. PP analysis demonstrates that preventive antibiotic therapy with moxifloxacin within 36 hours after stroke onset reduces infection rate in patients with severe ischemic stroke in the territory of the middle cerebral artery, while ITT analysis revealed only a trend towards beneficial effects for this treatment strategy. Patients in the placebo group were treated according to current treatment guidelines. With respect to antibiotics, they were fully and effectively treated as soon as antibiotic medication was indicated, i.e. as soon as an infection was diagnosed [13], [22]. Thus, PP analysis of our proof of principle trial provides for the first time evidence for a superiority of a preventive anti-infective therapy over current standard therapy.

Up to 95% of patients suffer from at least one relevant complication within the first three months after stroke [1]. Thus, for the majority of stroke patients, general measures targeting early identification and treatment of complications remain the major medical challenge. Complications impair functional and neurological outcome [2], [4], [8]. Treatment in dedicated stroke units was demonstrated to reduce the incidence of stroke-associated complications [23]. However, even in stroke units, infections after stroke remain common complications with a frequency between 21 and 65% [3]. Among infections, pneumonia have the greatest impact on the neurological outcome and mortality rate after stroke [5], [6], [8], [24]. Pneumonia represents the leading cause of in-hospital mortality of stroke patients, accounting for 31% of all death [8].

Currently, post-stroke pneumonia is thought to be a consequence of aspiration due to dysphagia and immobilization [2], [4], [25]–[27]. Forty to 70% of patients develop dysphagia within 3 days after stroke, 40% of them aspirate and approximately one third develop pneumonia [10], [28]. As stroke patients with dysphagia are at risk for pneumonia, post-stroke pneumonia is thought to be a preventable complication. Once dysphagia is diagnosed, measures can be taken aimed at prevention of aspiration [28], [29]. Feeding tubes are often used to prevent aspiration pneumonia [25], [30] but were also identified as the strongest independent predisposing factor for acute post-stroke infections [31]. In the PANTHERIS population, tube feeding is associated with a significantly higher risk of infections, however, it was not an independent risk factor for infections. Results from the large FOOD trials suggest that early tube feeding neither prevents nor favours infection rates [32].

Although post-stroke aspiration increases the risk of developing pneumonia by sevenfold, aspiration alone is not sufficient to explain the high incidence of pneumonia in acute stroke [2], [4], [25], [26], [33]. About 50% percent of healthy subjects also aspirate pharyngeal secrets every night to a similar extend as stroke patients, however, without developing pneumonia [34], [35]. Thus, our understanding of the high incidence of pneumonia in stroke patients is still incompletely. Since several immune dysfunctions have been reported in patients with acute CNS injury, stroke-induced changes in immunity may explain the high incidence of infections [3], [36], [37]. In a murine model of cerebral ischemia we provided experimental proof that a neuro-endocrine-mediated systemic immunodepression is an essential cause for a decreased antibacterial defense after acute ischemic stroke leading to post-stroke pneumonia [38]. We further demonstrated that otherwise harmless minor bacterial aspiration leads to severe pneumonia and bacteriemia after ischemic stroke suggesting that the deleterious combination of stroke-induced immunodeficiency with stroke-facilitated aspiration dramatically increases the susceptibility to infection [39]. Results from the PANTHERIS trial provides further evidence for a stroke-induced immunodepression in stroke patients causing post-stroke infections. As marker for immune competency we measured the monocytic HLA-DR expression, which is a well known and sensitive marker for a disturbed immune function in septic and critically ill patients [17], [40]–[42]. Here we describe a significantly reduced monocytic HLA-DR expression in the very early phase after stroke compared to 3 and 6 months after stroke. Remarkably, patients with infections in the placebo, but not verum group had significantly lower monocytic HLA-DR levels at days 1, 3, and 8 compared to non-infected patients. Notably, patients without infections in the verum group had significantly lower monocytic HLA-DR levels at the first day after stroke onset compared to patients without infections in placebo group corroborating that moxifloxacin treatment effectively prevented infections in a proportion of these patients. Furthermore, reduced monocytic HLA-DR expression at day 1 after stroke and before study treatment onset was a strong independent predictor of subsequent post-stroke infections in the placebo group, but not in the moxifloxacin group. Since there were no significant differences in the time course of monocytic HLA-DR levels between the placebo and verum groups, it is rather unlikely that post-stroke infections by itself cause the reduced HLA-DR expression.

Since the established general measures do not effectively prevent post-stroke infections, and in the light of our pathophysiological concept [36] of post-stroke infections and our preclinical data [38], Preventive Antibacterial Therapy (PAT) warrants further attention as promising new strategy. Using our experimental model of post-stroke infections, we have demonstrated that post-stroke infections can effectively be reduced by preventive antibiotic therapy with moxifloxacin. More importantly, in this stroke model moxifloxacin not only prevents the development of infections and fever, it also significantly reduces mortality, and improves neurological outcome [11]. Consequently, the primary endpoint of the PANTHERIS trial aimed at preventing severe stroke-associated infections, in particular pneumonia. Based on a retrospective analysis of stroke patients in participating stroke units infections arise between 2 and 7 days after stroke onset (data not shown). In order to cover this critical time period effectively, in PANTHERIS treatment was started 9 to 36 hours after stroke onset and continued for 5 days. In order to ensure that preventive antibacterial therapy not only shifts infections to a later time point infection rate was studied within 11 days post stroke. In the per protocol analysis, PANTHERIS demonstrated the efficacy of preventive anti-infective therapy with moxifloxacin. Compared to an infection rate of 41.9% in the standard therapy (placebo) group, only 17.1% of verum treated patients suffered from infections. Further evidence for the efficacy of preventive anti-infective therapy is the significant reduction of C-reactive protein in the moxifloxacin treated group, compared with placebo. Of all infections, predominantly pneumonia seems to be prevented, although this difference did not reach statistic significance. Mechanical ventilation, a known strong risk factor for nosocomial pneumonia [43], appeared to be more frequent in placebo compared to moxifloxacin group, again without reaching statistical significance level. Although we cannot exclude an inclusion bias, it would seem rather unlikely that the higher pneumonia rate in the placebo group should be caused by the higher frequency of mechanical ventilation in this group. On the contrary, since pneumonia preceded mechanical ventilation in the placebo-treated patient, our data suggest that PAT might reduce the need for mechanical ventilation in patients with acute stroke.

Stroke-associated infections lead to poor outcome [3], however the support for an independent causal relationship between infections and poor neurological outcome was recently challenged [37]. The PANTHERIS trial confirms that infections are associated with a significantly reduced survival rate. However, we remained short of demonstrating that patients treated with moxifloxacin should have improved clinical outcome or increased survival rate at 6 months after stroke, compared with patients treated according to current standards. These results seem to support the conclusion that stroke-associated infections are not the cause but rather a marker for a poor neurological outcome following stroke. However, since PANTHERIS was not sufficiently powered to address this issue, it remains open whether post-stroke infections lead to a worse outcome in stroke or not.

Chamorro and co-workers were the first to investigate in a randomized, placebo-controlled, double-blind trial the effect of preventive antibiotic therapy in stroke [44]. Their single-center ESPIAS trial included 136 patients using levofloxacin in a dose of 500 mg/d for 3 days starting within 24 hours after stroke onset. Based on an interim futility analysis, ESPIAS was prematurely stopped since levofloxacin neither prevented post-stroke infections nor improved outcome in patients with ischemic or hemorrhagic stroke. ESPIAS even suggested a non-significant rise of mortality rate in the verum group. Although both trials are similar in design, several differences may explain the discordant results. For example, in ESPIAS patients were treated sooner but shorter after stroke onset compared to PANTHERIS. Since the majority of stroke-associated infections occur within five days after stroke onset [33], [45], [46], the longer treatment period in PANTHERIS may be a crucial advantage covering this critical time period. Furthermore, in PANTHERIS moxifloxacin was chosen for its superior effect against aerobic pathogens [47], as local epidemiological data of stroke patients (unpublished data) demonstrated a predominating Gram positive spectrum (*Streptococcus pneumoniae, Staphylococcus aureus*) in pneumonia with a low incidence of moxifloxacin resistant pathogens such as MRSA and *Pseudomonas aeruginosa*. In addition, *in vitro* antibacterial activity of moxifloxacin against anaerobes is superior compared to levofloxacin [48], which might be of relevance, since anaerobic pathogens need consideration in aspiration pneumonia [49].

A major drawback of preventive antibiotic therapy is the potential induction of antibiotic resistance. The observation that susceptible *E. coli* isolates from one patient became resistant during moxifloxacin treatment indicates selection of fluoroquinolone-resistant bacteria by preventive treatment with moxifloxacin. However, the low *E. coli* re-isolation rate of 15% among patients treated with moxifloxacin (as compared to 70% in the placebo group) suggests moxifloxacin affects normal gut flora and was effective enough to eradicate *E. coli* in 17 out of 20 patients. The fact that in six patients of the placebo group, *E. coli* could not be re-isolated after treatment suggests that the intestinal *E. coli* populations were not solely affected by moxifloxacin treatment.

In conclusion, PP analysis of PANTHERIS suggests that preventive antibiotic therapy with moxifloxacin reduces the infection rate in patients with severe ischemic stroke in the MCA territory. Since PANTHERIS as proof of concept trial was not designed to demonstrate clinical superiority of preventive antibacterial treatment in a routine environment and ITT analysis revealed only a trend towards beneficial effects of preventive antiinfective treatment caution is needed. Analyses of secondary endpoints demonstrate a significant lower survival rate during 6 month follow-up for patients with infections. Even though infection rate was reduced in patients treated with moxifloxacin, survival and neurological outcome were not significantly improved compared to placebo. PANTHERIS provides the framework of data for our current planning of a phase III trial to further investigate the innovative concept of preventive antibacterial therapy in stroke patients focussing on effectiveness rather than efficacy. If post-stroke infections are independent risk factors for poor outcome and if they can be effectively prevented, there will be an urgent need for predicting factors of post-stroke infections. Indicators of immunodepression such as monocytic HLA-DR expression may be interesting candidate markers to identify patients at high risk for infectious complications, which warrant further confirmation in prospective trials.

## Supporting Information

Protocol S1 Trial Protocol
(3.93 MB PDF)

CONSORT S1 Checklist S1
(0.05 MB DOC)
