# Supplementary material for: Preventive Antibacterial Therapy in Acute Ischemic Stroke: A Randomized Controlled Trial
Source: PLoS One. 2008 May 14;3(5):e2158. doi: 10.1371/journal.pone.0002158 (PMC2373885; doi:10.1371/journal.pone.0002158)
Supplement: Protocol S1 — Trial Protocol (3.93 MB PDF) [file pone.0002158.s001.pdf]

Research Protocol

# of the

Pilot Study

on

preventive antibacterial short-term therapy on patients with acute, MCA territory ischemic infarction

pantheris

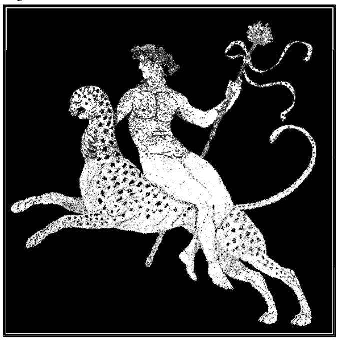

preventive antibacterial therapy in stroke

Table of Contents

1 Introduction.......5
1.1 Ischemic Stroke 5
1.2 Complicating Infections....... 5
1.3 Current State of Research 6
1.4 Own Preparatory Work 7
1.4.1 Retrospective Analysis of Severe Ischemic Strokes....... 7
1.4.2 Infections and Immune Depression after *Experimental* Stroke....... 10
1.4.3 Preventive Antibacterial Therapy after *Experimental* Stroke....... 10
1.5 Hypotheses and Objectives 11
1.6 Methods....... 11
1.6.1 Number of Patients and Statistics 12
1.6.2 Moxifloxacin For Antibacterial Therapy....... 13
1.7 Ethical Problems 16
1.7.1 The Risk of the Bacterial Development of Resistance 16
1.7.2 Patients who are Incapable of Giving Their Consent....... 16
2 Research Protocol 17
2.1 Designation of the Study 17
2.2 Research Group....... 17
2.2.1 The Scientific Project Management....... 17
2.2.2 The Clinical Examination Management....... 17
2.3 Sponsors....... 17
2.4 Endpoints and Objectives Criteria 18
2.4.1 Primary Endpoint (Main Objective Criterion) 18
2.4.2 Secondary Endpoints and/or Subordinate Objectives Criteria....... 18
2.5 Research Design....... 19
2.6 Duration of the Study....... 19
2.7 Number of Patients....... 19
2.8 Investigational Medical Product for the Study....... 19
2.9 Dosage, Administering and Duration of Treatment....... 20
2.10 Inclusion Criteria 20
2.11 Exclusion Criteria....... 20
2.12 The Documentation of Findings 22
2.12.1 Data Collection Prior to the Commencement Of the Study 22
2.12.2 Data Collection during the Study 23
2.13 Accompanying Medication 25
2.14 Complications....... 25
2.15 The Safety of the Study Patients 26
2.16 The Reporting of Serious Adverse Events (SAE) 26
2.17 Interruption of the Study....... 26
2.18 Statistical Methods....... 27
2.18.1 Primary Aim of the Study 27
2.18.2 Secondary Aims of the Study 27
2.18.3 Research Design....... 27
2.18.4 Hypotheses....... 27
2.18.5 Statistical Methods and the Planning of the Scope of the Study 28
2.18.6 The Definition of Evaluation Collectives 29

2.18.7 Randomisation 29
2.18.8 Stratification and De-Blinding 29
2.18.9 Quality Assurance 30
2.18.10 Data Management 30
2.19 Ethics 32
2.19.1 The Declaration of Helsinki 32
2.19.2 Ethics Commission 33
2.19.3 Informing the Patients 33
2.19.4 Consent to Participation in the Study 33
2.19.5 The Use, Storage and Communication of Data 33
2.19.6 Amendments to the Protocol 33
2.20 Legal and Administrative Regulations 34
2.20.1 GCP, Fundamental Legal Principles 34
2.20.2 Patients Insurance 34
2.20.3 Funding 34
2.20.4 Final Report and Publication 34
2.20.5 Adherence to the Protocol and the Protocol Amendments 34
2.21 Study Flowchart 35
3 Literature 36
4 Enclosure: 36
4.1 Clinical Evaluation Scales 36
4.1.1 NIH Stroke Scale 36
4.1.2 Scandinavian Stroke Scale (SSS) 39
4.1.3 Barthel Index 41
4.1.4 Stroke Impact Scale 42
4.1.5 Montgomery Asberg Depression Rating Scale (MADRS) 47
4.1.6 Multi Organ Failure Score (MOF-S) 53
4.2 Microbiology 55
4.3 Infection Definitions 56
4.3.1 Definition Pneumonia 56
4.3.2 Definition of Sepsis 58
4.3.3 Urinary Tract Infection 58
4.4 Immunology 60
4.4.1 Small Immune Status 60
4.4.2 Whole Blood LPS Stimulation (TNFa) 60
4.4.3 Whole Blood ConA Stimulation 61
4.4.4 T-Cell Stimulation with MBP/PLP/TT 61
4.4.5 Procalcitonin (PCT) 62
4.4.6 DC-PCR 62
4.5 Biochemistry 63
4.5.1 AP, Plasma 63
4.5.2 AST (GOT), Plasma 64
4.5.3 Bilirubin (whole), Plasma 65
4.5.4 Large Haemogram, Blood 66
4.5.5 Cortisol, 24-hr Urine 67
4.5.6 CRP, Plasma 68
4.5.7 Glucose, Plasma 69
4.5.8 Potassium, Plasma 70
4.5.9 Creatinine, Plasma 71
4.5.10 Sodium, Plasma 72

4.5.11 Noradrenaline, 24-hr. Urine 74
4.5.12 Urine Strip Test 75
4.6 The Manufacturing of the Investigational Medical Product 76
4.6.1 Moxifloxacin (Verum) 76
4.6.2 Riboflavin (Placebo) 76
4.7 AVELOX®– product characteristics 77
4.8 The Declaration of Helsinki 92
4.9 Medicinal Products Act (Extract) 96
4.10 Information Sheet for Patients **16/07/2002** 99
4.11 Declaration of Consent 111

1 Introduction

1.1 Ischemic Stroke

The ischemic stroke is the most frequent acute neurological disease. More than 200,000 Germans suffer from ischemic stroke every year, there are almost 2 million people currently living in Germany who have survived a stroke. In most cases, they are left with serious disabilities. Hence stroke is the most frequent cause of disabilities in adults.

The ischaemic stroke is primarily caused by a disturbance of the blood circulation in parts of the brain as a result of a occluded blood vessel. In this case, two different zones may be distinguished: An absolute ischaemic zone, in which, a few minutes after the occlusion of the blood vessel, the lack of blood supply has led to an irrevocable collapse of the *structural metabolism* (ischaemic **core**) - and a r**elative ischaemic zone,** in which, although it is not possible to maintain the current functional metabolism, by normalising the blood flow, it is possible to recover the cerebral tissue (**penumbra).** While the size of the area taken up by the ischaemic core is determined by the anatomical position of the occlusion in the blood vessel and, within the space of minutes, is final, even hours and days after the primary ischaemic event, various mechanisms contribute to an expansion of the ischaemic core, i.e. of the final infarct, causing further damage to the penumbra, which was initially only paralysed. These mechanisms include adverse excitotoxic effects (e.g. caused by the release of glutamate), cell-electric instability (peri-infarct depolarisations), cytotoxic noxes (e.g. radicals), infectious processes (e.g. the release of cytocine) and apoptosis (Dirnag U et al. 1999). This applies to the thrombolysis and to the intended slightly hypertone adjustment of the blood pressure, aimed at normalising the blood flow in the area of the penumbra and also intended to normalise the blood sugar level and to lower the excessive body temperature after a stroke.

1.2 Complicating Infections

Experience and clinical studies show that the stroke is associated with a high incidence of severe, complicating infections (Davenport et al. 1998, Georgilis et al. 1999, Langhorne et al. 2000, Castillo et al. 1998, Johnston et al. 1998, Grau et al. 1999).

By means of an experimental stroke model, using animals, we were able to show, for the first time, that a stroke-induced immune suppression considerably exacerbates this problem. A retrospective evaluation of stroke patients at our clinic supports these findings, also in the clinical field. In the clinical project described here, it shall be examined whether preventive antibacterial short-term therapy, in comparison with the currently practised post hoc antibacterial therapy already begun, reduces morbidity and lethality after acute strokes. To this end, a controlled double-blind trial shall be conducted. Concerning the *confirmative* answer to the question as to whether the effectiveness of a preventive antibacterial treatment additionally serves to prevent complicating infections in the acute phase, this trial shall contain an *explorative* examination of the supposed improvements, also of the neurological success of the treatment, and in addition shall provide a purely descriptive characterisation of the microbiological and immunological parameters of the immune suppression, not examined so far, after an acute stroke.

1.3 Current State of Research

The only scientifically founded, specific, acute therapy for the stroke is currently the systemic or local thrombolysis of the arterial occlusion. However, since, this has to be carried out within three to six hours after symptom onset, it is only able to benefit 5% to a maximum of 10% of the stroke patients.

All pharmacotherapeutic, neuroprotective approaches, in spite of numerous positive experimental studies on animals, have not proved very effective in human therapeutic studies. Primarily for this reason, the main attention of the therapy is still focussed on the optimal adjustment of such unspecific parameters as blood pressure, blood sugar and body temperature as well as ethiopathogenetic, specific secondary prophylaxis.

Clinical observations suggest that an immune depressive state arises also in consequence of a stroke (Howard & Simmons 1974). The patient suffering from stroke is particularly at risk in the acute and early remission phase, not only from the dangerous space consuming oedemas of a large-scale infarct and from the risk of renewed disturbances of the cerebral blood flow, but also in high degree from infections. Infections, and in particular pneumonia, constitute the main cause of lethality in the case of a stroke (Henon et al. 1995). In a non-selected patient population 16% to 61% developed pyrexia, 21 to 65% infections and 10% to 22% pneumonia (see table below).

n	Pyrexia	Infections	Pneumonia	HWI	Literature
607		28%	12%	16%	Davenport et al. 1996
330	38%	23%	10%	11%	Georgilis et al. 1999
311		65%	22%	24%	Langhorne et al. 2000
297	61%	35%			Castillo et al. 1998
279	16%		10%	11%	Johnston et al. 1998
119		21%	16%	3%	Grau et al. 1999

In comparison with the prevalence of nosocomial infections, given in the relevant literature at 4% to 9% (Kampf et al. 1997, Emmerson et al. 1996, Anderson et al. 2000, Gikas et al. 2002) and among post-operative patients at roughly 3% (Smyth & Emmerson 2000), the high rate of infection in the case of stroke patients is particularly striking. A systemic trial was able to show that, on the first and second day after an infarct, the risk of infection is at its highest (Grau et al. 1999). The risk of infections extended beyond the acute phase. For example, also during rehabilitation, complicating infections are frequent (Kalra et al. 1995, Black-Schaffer et al. 1999). Kong et al. (1998) report an infection incidence of 32%. Among these, infections of the urinary tract predominated.

The heterogeneity of the data available in the relevant literature concerning complicating infections in the case of strokes, reflects the spectrum of ischaemic strokes from reversible deficits extending to subtotal or total hemispherial infarcts. In view of our own preparatory work (see below), it is plausible to assume that, among the data cited, large ischaemia correlate with high rates of infection.

For this reason, the proposed trial shall initially examine that subpopulation of stroke patients for which the highest incidence of infection may be expected: namely the group suffering from serious strokes, which shall be characterised more precisely below.

1.4 Own Preparatory Work

1.4.1 Retrospective Analysis of Severe Ischemic Strokes

We conducted a retrospective evaluation of 173 patients with severe infarcts, who, during the last three years, had been under our intensive medical care. For the purposes of this retrospective trial, the indicator for the degree of severity of the strokes was the necessity to undertake intensive medical treatment. (In the proposed, prospective trial, instead of this surrogate parameter, neurological scales shall be used for measuring the severity of the infarctions.)

In the epicrises of the 173 stroke patients analysed retrospectively, pneumonia was diagnosed in 39% and sepsis in 6%.

Within the first week, fifty percent of the patients showed distinct lymphopenia, being an indication of immune suppression.

For 105 of the 173 patients under hospital treatment in the period mentioned, it was possible to conduct a detailed retrospective evaluation of the hospital records. The remaining patients could not be included in the exact analysis for formal reasons, either because, at the time of the stroke, there existed comorbidity, for example with previously existing infections, neoplases or large-scale surgical interventions or because the records did not contain enough information or could not be found.

The following graph shows the cumulative incidence of clinical or paraclinical signs of infection (pyrexia, leucocytosis, CRP increase), for 105 patients prior to intubation and antibiotic therapy and/or the death of the patients over the treatment period in days:

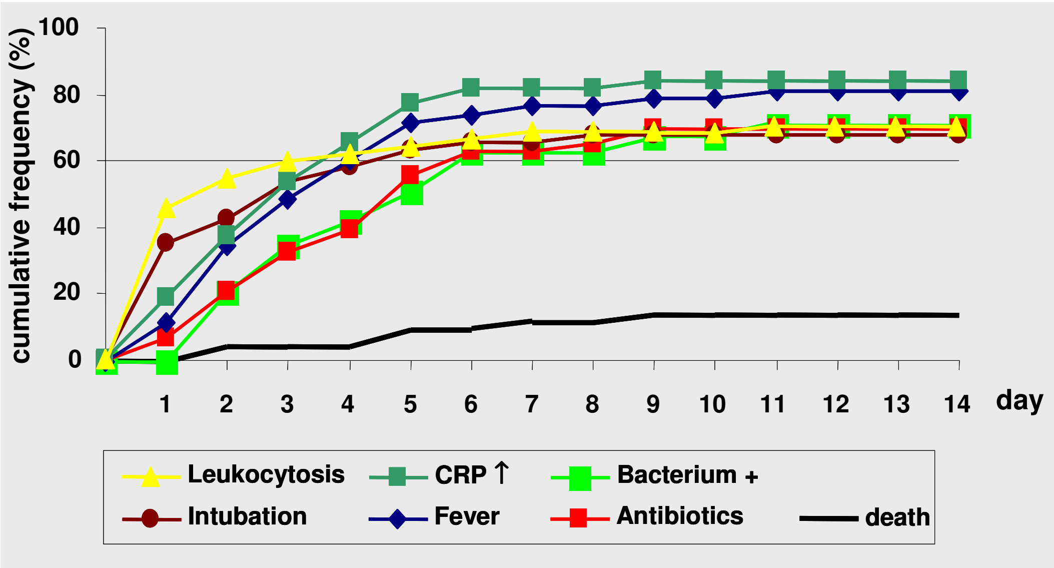

MCA-infarction	n = 71	1998-2001
age = 66 (34-88)	w/m = 32 / 39	ICU Neurology/Charité

The following three tables show the frequency of typical infection parameters and the median and or the mean incidence, divided according to the localisation of the ischaemia:

All Infarctions

n=105; 64 m; 41 w

		Temp. >37,5°C	Temp. >38°C	CRP>3	Leucocytes >10	Intubation	Chemotherapy	death
**Freuency**		**81%**	**64%**	**75%**	**74%**	**55%**	**61%**	**16%**
	**Age**	**d**	**d**	**d**	**d**	**d**	**d**	**d**
**Median**	65.0	2.0	3.0	3.0	1.0	1.0	4.0	5.0
**Mean**	63.5	2.4	3.3	3.2	1.9	2.1	4.3	5.8
**Standard-dev.**	13.9	1.7	2.3	2.2	2.0	1.7	2.4	3.8

Infarctions in the MCA Flow Territory

n=71; 37 m; 34 w

		Temp. >37.5°C	Temp. >38°C	CRP>3	Leucocytes >10	Intubation	Chemotherapy	Death
**Frequency**		**84%**	**68%**	**76%**	**69%**	**54%**	**61%**	**14%**
	**Age**	**d**	**d**	**d**	**d**	**d**	**d**	**d**
**Median**	64.5	2.0	3.0	3.0	1.0	1.0	4.0	5.0
**Mean**	64.3	2.5	3.5	3.4	1.7	2.1	4.2	6.1
**Standard-dev.**	13.6	1.8	2.3	2.0	1.4	1.7	2.2	4.5

Infarctions exclusively in the PCA Flow Territory

n=31; 23 m; 8 w

		Temp. >37.5°C	Temp. >38°C	CRP>3	Leucocytes >10	Intubation	Chemotherapy	Death
**Frequency**		**74%**	**64%**	**71%**	**84%**	**55%**	**58%**	**19%**
	**Age**	**d**	**d**	**d**		**d**	**d**	**d**
**Median**	63.0	2.0	2.0	2.0	1.0	1.0	3.0	5.0
**Mean**	59.0	2.0	2.8	3.0	2.0	2.0	4.3	4.8
**Standard-dev.**	14.3	1.2	2.2	2.7	2.0	1.9	2.8	1.5

Other Infarction Locations

Infarctions exclusively in the ACA Flow Territory n=2

Non-classified Infarctions n=1

The following bacterial pathogens were identified in 105 retrospectively analysed patients during the first 14 days after the stroke (n = number of analysed material samples).

**blood culture (n = 21)**	%	**absolute**
Staph. hominis	19	4
Staph. aureus	10	2
Acinetobac. junii	5	1

**midstream urine (n = 9)**	%	**absolute**
E. coli	33	3
Clebsiella pneumonia	11	1
Proteus mirabilis	11	1
Pseudomonas aeruginosa	11	1
Enterococc. faecium	11	1

**CVC (n = 17)**	%	**absolute**
Staph. epidermidis	41	7
coagulaseneg. Staph.	35	6
E. coli	6	1
Enterococc. faecium	6	1
Serratia marcescens	6	1
Staph. chromogenes	6	1

**Tracheal secretion (n = 180)**	%	**absolute**
Staph. aureus	21	29
gramneg. pathogenic rods	4	8
other ß-hemolis Streptoc.	5	7
Hemophilus spp.	3	6
E. coli	3	5
Proteus mirabilis	3	4
Pseudomonas aeruginosa	3	4
other ß-hemolis Streptoc. Gr B	3	4
Haemophilus influenzae	1	2
Proteus vulgaris	1	2
Streptoc. pneumoniae	1	1

**Bronchial secretion (n =29)**	%	**absolute**
Hafnia alvei	10	3
E. coli	3	1
Enterobact. sakazakii	3	1
gramneg. pathogenic rods	3	1
Proteus mirabilis	3	1
Staph. aureus	3	1

1.4.2 Infections and Immunedepression after *Experimental* Stroke

Starting from the clinical observation of a clinical immune depression, arising in connection with an acute stroke, we characterised this phenomenon in an experimental stroke model, using animals (Prass et al. submitted).

Among other things, we were able to prove that, already six hours after an extended cerebral ischaemia, a massive lymphopenia of the T, B and NC cells occurs, which is sustained over a period of 14 days. This is caused by excessive activation of the sympathetic and partially of the hypothalamus-hypophyses-adrenal-glands system.

In addition to the decrease in the number of immune competent cells in the blood, the activation of the sympathetic nervous system additionally leads to a functional deactivation of the remaining cell pool.

In our trial, the drastically increased susceptibility to infection connected with this after an acute stroke in the mouse model appears to be of clinical relevance. The animals spontaneously developed a severe pneumonia 1 -3 days after the stroke. In addition, roughly 50% of the stroke animals showed the clinical symptoms of a sepsis. The infection led to the death of roughly 60% of the animals between the fifth and seventh day.

Also in the case of the surviving animals, we were able to show, in a pneumococcal infection model, an increase in susceptibility to infection 14 days after the stroke. The central importance of the cerebral ischaemia becomes apparent in a comparison with the so-called sham control. Apart from the ischaemia, the control animals were subjected to the same operative interventions and stresses, but showed no immune depression and hardly any infections.

The drastically increased susceptibility to infection is basically and primarily caused by a massive disturbance of the T and NC cell functions. Here, the excessive activation of the sympathicus appears to be of great causal significance (Prass et al., in preparation). Other authors have demonstrated that such a simpathicotonic reaction is accompanied by infarctions of the insular region (Sander D et al. 2001;Tokgozoglu SL et al. 1999).

1.4.3 Preventive Antibacterial Therapy after *Experimental* Stroke

If we regard the current therapeutic options in the case of an acute stroke, it becomes clear that serious infections have an influence on two parameters, for which an influence on the final size of the infarct and the remaining deficits after a stroke could be shown: the body temperature and the blood pressure (Dirnagl et al. 1999). Moreover, additional systemic mechanisms may be assumed, which in the course of serious infections, cause a secondary extension of the damage to the cerebral tissue to the detriment of the so-called *penumbra,* for example, the release of pro-inflammatory cytocines.

In view of the relationships between the internistic and infection-correlated phenomena and the evolution of the infarct in the first days after the ischaemic event, it is plausible to suppose that the prevention of serious infections also brings with it an indirect neuroprotective effect.

For this reason, we set out to examine, in our stroke model of the mouse, the question as to whether an early, preventive therapy with antibiotics lowers lethality and has a neuroprotective effect. Our data showed that administering Moxifloxacin reliably prevented the occurrence of serious infections. In this way, in our model, it was not only possible to considerably lower lethality but also the infarction volumes and the neurological deficits of the experimental animals were reduced (Prass et al. 2002).

Thus, we were able to prove that, in consequence of an extended stroke, infectiologically relevant immune suppression occurs, a phenomenon which is known in the course of other critically acute diseases, such as myocardial infarctions, polytrauma - but also with large-scale operative and, above all, neurosurgical interventions (Livingston et al. 1998, Döcke et al. 1997, Woiciechowsky et al. 1998).

1.5 Hypotheses and Objectives

The central hypothesis of our project is:

> A preventive, antibacterial, short-term therapy lowers secondary morbidity -both as regards to complicating infections as well as concerning the success of neurological rehabilitation - and lowers lethality after a severe, acute stroke.

This hypothesis is too complex to be verified within the framework of a small-scale trial conducted solely under our own responsibility. It appears to be expedient, therefore, to approach the answer to this question in several steps.

In the currently proposed trial, therefore, we seek *confirmative* verification of the following hypothesis.

1. An early, preventive, antibacterial short-term therapy lowers the incidence of complicating infections after an MCA territorial infarction

The further reaching hypothesis claims that:

2. A preventive, antibacterial, short-term therapy in the first days after the stroke, in comparison with an antibacterial post-hoc therapy, reduces the infarct volume and the neurological deficit after an MCA territorial infarction.

Within the framework of this small-scale trial, it will only be possible to conduct an *explorative* examination of this hypothesis. The data obtained in this trial shall serve to enable the consideration and planning of further studies.

Furthermore, in the descriptive immunological and microbiological approaches of the trial, the following hypotheses shall be examined:

3. The stroke causes relevant immune depression in patients, just as in the animal model.
4. This immune depression is communicated through the sympathetic nervous system.
5. The preventive, antibacterial, short-term therapy with Moxifloxacin can lead to the development of resistance in facultative pathogenic bacteria.

1.6 Methods

In view of the limited dimensions of this trial and starting from the correlation of the probability of the incidence of complicating infections with the size of the infarct, only those patients with *large-scale MCA territory infarctions* shall be examined in this trial.

It shall be endeavoured to integrate a further centre into the trial. This shall be a clinical, neurological establishment whose patient population enables an adequate recruitment and whose infrastructure permits the examination and procurement of material in line with the aims of this trial. The processing of the microbiological and immunological materials shall be undertaken for all patients in the respective institutes of the Charité.

The patients shall be recruited from the acute stroke patient population admitted at both centres.

The statistical planning, the randomisation and the stratification of the trial shall be undertaken by The Institute for Medical Biometry of the Charité.

The clinical execution of the trial shall be placed in the hands of a qualified investigating physician at each of the centres.

The planning of the microbiological and infectiological aspects of the trial, as well as the processing of the microbiological materials, shall be guaranteed by The Institute for Microbiology and Hygiene of the Charité.

The processing of immunological questions shall be undertaken by the Institute for Immunology.

1.6.1 Number of Patients and Statistics

The estimation of the necessary number of patients was undertaken in cooperation with the Institute for Medical Biometry.

It shall be endeavoured to achieve a *confirmative* verification of Hypothesis 1 (see 1.5). This means that patients (A), treated *post hoc* according to the conventional therapy principle with antibiotics in the case of an infection, for the occurrence of complicating infections (main endpoint see 2.4.1) shall be compared with patients (B), who have received preventive, antibacterial treatment.

On the basis of scientific literature and our own retrospective trial, we shall assume a complicating infections rate of 40% for Group A, whereas we presuppose an infection rate of 10%, at the most, for Group B.

Both complication rates are conservatively estimated. In our retrospective review of severe stroke cases, the spontaneous infection rate was higher. For the infection rate under treatment with the broadband antibiotic Moxifloxacin, on the other hand, there are no data available.

On the basis of the presupposed numerical data, the verification of Hypothesis 1, the minimum size of Groups A and B, applying a power of 0.8, may be determined at 32 patients for each group respectively.

For the further reaching Hypothesis 2, which poses the question as to the *neurologically measurable benefit* of a preventive, antibacterial therapy, given the incomparably greater complexity of the data, the number of patients would have to be considerably higher . This hypothesis shall be examined *exploratively*.

When calculating the number of patients, we must expect that some of the trial patients will be very critically ill. For such patients the "therapia minima" must be seriously taken into consideration as a serious and responsible option for medical treatment and, for this reason, a comparatively high drop-out rate can be expected. We estimate this rate at 25%.

From this, the number of patients to be recruited is calculated at 80, 40 per group.

The patients shall be stratified, randomised and allocated to the respective group according to age (≤64 Jahre/>64 Jahre), sex and the side of the MCA infarction. The stratification according to age is conducted on the basis of our retrospective trial, in which the median age value was 64.5 years of age. Stratification according to the side affected by the ischaemia shall be conducted because a dependency of vegetative reactions on cerebral lesions according to which side is affected has been demonstrated (Sander D et al. 2001; Tokgozoglu SL et al. 1999).

1.6.2 Moxifloxacin For Antibacterial Therapy

As already described above, bacterial infections of the deep respiratory tracts constitute the majority of complicating infections following the ischaemic stroke. Here, bacterial pneumoniae are in the foreground. In this trial, therefore, it appears important to use an antibiotic, in the treatment of infections of the deep respiratory tracts, which is proven to be sure and practicable.

Furthermore, the antibiotic used shall have a broad antibacterial effectiveness and a narrow spectrum of side effects.

Moxifloxin (AVALOX®), approved in 1999, is a methoxy-fluoroquinolone, which has a high antimicrobial effectveness against the most important bacterial pathogenes of inflammation of the respiratory tracts, such as *Streptococcus pneumoniae, Haemophilus influenzae, Moraxella catarrhalis, Clebsiella pneumoniae* and atypical organisms *(Mycoplasma pneumoniae, Chlamydia pneumoniae, Legionella pneumophila).*

Like most fluoroquinolones, Moxifloxacin is very effective against gramnegative bacteria. However, it distinguishes itself, for example, from the widely used Ciprofloxacin, by virtue of its considerably broader spectrum of effectiveness against grampositive bacteria, atypical pathogenes (*Mycoplasma pneumoniae, Chlamydia pneumoniae, Legionella pneumophila*) and Anaerobians. In comparison with Ciprofloxacin, Moxifloxacin is less active against *Pseudomonas aeruginosa* (Bauernfeind 1997). On the other hand, its effectiveness in regard to grampositive pathogenes, compared with Ciprofloxacin, particularly against Staphylococcs, is considerably stronger.

From the therapeutical point of view, the effects of Moxifloxacin against Pneumococcs are particulary interesting. Both penicillin-sensitive as well as penicillin-resistant strains of *S. pneumoniae* are inhibited by concentrations of 0.125 mg/l. In comparison with Ciprofloxacin, this means a roughly eight-fold increase in the effectiveness (Barman-Balfour et al. 1999, Blondeau 1999, Buxbaum et al. 1999). Thus, Moxifloxacin encompasses the clinically most important pathogenes (Bartlett et al. 2000) of infections of the respiratory tracts, which is why many authors count it among the group of „respiratory" Fluoroquinolones (Lode 2001). Fluoroquinolones are named in the current recommendations of the American Association for Infectious Diseases (Bartlett et al. 2000) for the treatment of ambulantly contracted, bacterial pneumoniae as a first-choice means. Also, proposals for the treatment of early, nosocomial pneumonia suggest Moxifloxacin as a sensible, empirical therapy option (Ewing et al. 2002).

The antimicrobial effect of fluoroquinolones is based on the inhibition of two enzymes essential for the bacterial DNA metabolism: the topoisomerases. In this, Moxifloxacin interacts both with the so-called DNA-Gyrase as well as with the Topoisomerase IV.

Both enzymes are indispensable, among other things, for the replication, transcription, recombination and repair of the DNA. Thus, it is a matter of a two-pronged antibacterial attack. In contrast to other antibiotics, Moxifloxacine develops its bactericidal effect according to the dosage.

After the intravenous application of 400 mg of Moxifloxacin, peak concentrations in the plasma of up to 4.6 mg/l are measured. The plasma half-life period is around 13 hours, which enables the administering of a single dose (Stass et al. 1998).

Moxifloxacin not only distributes itself extracellularly but also achieves high intracellular concentrations. This behaviour manifests itself in a high distribution volume, which, for 400 mg of Moxifloxacin, has been calculated at 3.5 l/kg. Above all in the pulmonary compartments (mucosa, epithelial fluid film, alveolarmacrophages) significantly higher concentrations than in the plasma were measured (Stass et al. 1998; Barman-Balfour et al. 1999, Wise 1999).

Naturally, with every preventive antibacterial treatment, the risk of developing resistance is a principal problem. Moxifloxacin, however, appears to be preferable to other active agents also in this respect.

The resistance of bacteria against fluoroquinolones occurs through mutations of the genes which encode the target enzymes of the bactericidal effect (DNA-Gyrase, Topoisomerase IV). Moreover, bacteria can reduce the intracellular concentration of the active agent. With Methoxy-Fluoroquinolones (Moxifloxacin) the potential development of resistance appears to be lower than with fluoroquinolones of the Groups 2 and 3 (Ciprofloxacin/Levofloxacin) (Dalhoff 2001; Pong et al. 1999).

This favourable property is attributable, in particular, to two pharmacodynamic parameters: the „*mutant prevention concentration* (MPC)“ and the „*mutant selection window* (MSW)“. The MPC is to be regarded as the „threshold concentration“, above which these mutations cannot be detected. Ideally, this value should lie below the maximum concentration of the active agent which is achieved by normal dosage. For Moxifloxacin, the MPC (determined for *Streptococcus pneumoniae*), at 2 µg/ml, lies significantly below the maximal concentration of the active agent of 4.5 µg/ml with normal dosage (Blondeau et al. 2001).

The "*mutant selection window*" describes that concentration area in which mutants can be selected. This lies between the minimal inhibition concentration (MIC) and the MPC, as described above. Below the MIC, as a result of the lack of pressure to select, no mutants are selected, while above the MPC, mutants, by definition, do not occur. If this window can be kept small (*mutant selection window*), the risk of developing resistance is reduced. Bascially, this strategy aims at the pharmacokinetic convergence of the MIC and the MPC.

Moxifloxacin, in this sense, possesses pharmacodynamic and pharmacokinetic properties which counteract the development of resistance. In comparison with other clinically relevant fluoroquinolones (Ciprofloxacin, Levofloxacin) - but also with antibiotics of other classes of active agents (Penicillin G, Tobramycin), with Moxifloxacin, therefore, a considerable reduction of the potential development of resistance may be expected (Zhao et al. 2002).

In the case of critically ill patients and stroke patients, intravenous galenicals are preferable to oral galenicals, since these patients frequently have problems in swallowing.

During Phase II and III studies it was found that the undesirable effects caused by Moxifloxacin were mostly mild and of a temporary nature. A meta-analysis (Springsklee et al. 1999) of 20 clinical Phase II and III studies conducted so far (4926 patients) showed that the most frequent undesired effects, nausea (7.2 %) and diarrhoea (5.7 %) occurred. Of the patients treated, 2.8% reported feelings of dizziness. Amedicinal product-induced phototoxicity, known in the case of other quinolones, was not observed. In 3.3% of the patients, the therapy had to be broken off because of side-effects, corresponding to the dropout rate in other clinical studies with penicillin, cephalosporines, macrolides.

Thus, in the clinical trial, the findings of the preclinical experimental trials on animals and the experience gained with healthy test persons were confirmed, leading to the conclusion that Moxifloxacin possesses high tolerability.

In conclusion, it may be said that Moxifloxacin combines a broad spectrum of effectiveness with a low potential for the development of resistance, a favourable side-effects profile, as well as optimal pharmacokinetic properties and, therefore, seems particularly suitable for our trial.

1.7 Ethical Problems

1.7.1 The Risk of the Bacterial Development of Resistance

Every dosage of antibiotics is accompanied by a certain risk of the bacterial development of resistance. On principle, therefore, it is necessary, with every application, to consider the balancing of interests between the individual benefits and the medico-ecological risks to be borne by society. Whereas this consideration poses no great difficulty when it comes to the treatment of severe infections, in the case of preventive therapy, it is more problematic.

It may be considered beyond doubt that the application of the antibiotic Moxifloxacin in the concrete framework of this trial essentially constitutes no medico-ecological risk. This question becomes relevant, however, when as a result of the clinical studies on preventive antibacterial short-term therapy for stroke patients, such a therapy shall be standardised and applied on a large scale.

We endeavour to address the ethical problems involved by making the molecular biological examination of the potential development of resistance an essential component of the trial. We are striving to increase our knowledge, not only in respect of the *intended effects* of the new therapy principle, but are also examining its *problematic* aspect. Hence, it is possible that this trial may result in data which provides arguments against the standardised, preventive use of the antibiotic Moxifloxacin.

1.7.2 Patients who are Incapable of Giving Their Consent

The predominant proportion of the patient population to be examined will be limited in its capability to express consent. The typical clinical symptoms of the patient group to be examined include impaired consciousness, speech disturbances and a limited capability for making decisions. If the left cerebral half of the MCA territory is affected, the patients are mostly suffering from a form of aphasia; if it is the right cerebral half, infrequently from hemineglect and not infrequently from anosognosia. Without the participation of these patients, an examination of the large-scale MCA infarction is not possible.

As a matter of principle, the consent of the legal representative of patients incapable of expressing consent themselves is indispensable for the participation of such patients in this trial.

2 Research Protocol

2.1 Designation of the Study

Pilot Study on Preventive Antibacterial Short-term Therapy on Patients with Acute, MCA Territory Ischaemic Infarction

2.2 Research Group

The research group comprises the following physicians and scientists:

Prass K, Harms H, Volk HD*, Halle E**, Göbel, U**, Wernecke K-D#, Dirnagl U, Arnold G, Einhäupl KM, Meisel A / Charité Berlin, Neurological Clinic and/or *Institute for Immunology, **Institut for Mikrobiology and #Institute for Medical Biometry.

Further physicians shall be incorporated into the research group on the participation of a further neurological centre.

2.2.1 The Scientific Project Management

Dr. med. A. Meisel, Dr. med. K. Prass

Neurological Clinic of the Charité
Campus Charité Mitte
Schumannstr. 20/21
10117 Berlin

2.2.2 The Clinical Examination Management

PD Dr. med. G. Arnold

Neurological Clinic of the Charité
Campus Charité Mitte
Schumannstr. 20/21
10117 Berlin
Telephone: 030-450 560 082
Fax: 030-450 560 922

Since 1993, the clinical trial management has been performing Phase II, III, and IV studies at the Humboldt University and Neurological Clinic of the Charité.

2.3 Sponsors

This trial shall be conducted on the initiative of the Neurological Clinic and shall be led by physicians of the Charité.

The trial shall be funded largely by the Charité, the Hermann-and-Lilly-Schillung Foundation, as well as by the German Research Foundation and to a smaller extent by third-party funds, provided by the company BayerVital, 51368 Leverkusen.

2.4 Endpoints and Objectives Criteria

In all the main and subordinate objectives criteria of the trial, the following shall be compared:

A) a group of stroke patients which, immediately at the onset of a treatable infection, shall receive antibacterial treatment (normal therapy approach), with

B) a group of stroke patients which shall receive early, preventive antibacterial short-term therapy (**new therapy approach**).

2.4.1 Primary Endpoint (Main Objective Criterion)

1. The occurrence of a c**omplicating Infektion**♦ up to the 11th day after a large-scale MCA territory infarction.

2.4.2 Secondary Endpoints and/or Subordinate Objectives Criteria

2. **internistic state** measured with MOF-score on the 11th day after a large-scale MCA territory infarction.
3. **Death** of the patients up to the 11th day, as well as up to three and six months after a large-scale MCA territory infarction.
4. **Neurological deficit** and/or **disability**, measured with the *NIH-Stroke Scale* (NIH-SS), of the *Scandinavian Stroke Scale* (SSS), the *Barthel-Index* (BI) and with the *Stroke Impact Scale* (SIS) on the 11th day, as well as 3 and 6 (±5 d) months after a large-scale MCA territory infarction.
5. The occurrence of a **complicating infection**♦ up to the third month after a large-scale MCA territory infarction.
6. **Stroke volumetry** 3 months (±5) days after a large-scale MCA territory infarction.
7. The occurrence of **pyrexia** up to the 11th day after a large-scale MCA territory infarction.
8. Frequency of the prescription of antipyretics up to the 11th day after a large-scale MCA territory infarction.
9. **duration of the acute treatment** at the Stroke Unit or intensive care unit until being transferred to a normal care unit or to a rehabilitation establishment.
10. The **existence of a depression** measured with The Montgomery Asberg Depression Rating Scale (MADRS) six months (plus/minus five days) after large-scale MCA territory infarction.
11. The frequency of ***adverse events***.

Apart from these objectives criteria, the data gathered shall serve to characterise immunological parameters, in particular in regard to the supposed immune depression after a stroke and to characterise microbiological parameters after large-scale MCA

♦ according to criteria after CDC and RKI (see Annex)

territory infarction and/or after preventive treatment with Moxifloxacin; in particular in regard to resistance-causing mutations.

2.5 Research Design

This shall be in the form of a prospective, controlled, randomised, double-blind trial at two centres..

The second centre is still to be determined. This shall be a clinical, neurological establishment whose patient population enables an adequate recruitment and whose infrastructure permits the examination and procurement of material in line with the aims of this trial.

The processing of the microbiological and immunological materials shall be undertaken for all patients in the respective institutes of the Charité.

2.6 Duration of the Study

The estimated duration of the trial is approximately 24 months.

The acute clinical part (recruitment and intervention) will take approximately 18 months. This will be followed by the *follow up* and evaluation phase.

The trial period for each individual patient shall be six months.

2.7 Number of Patients

The trial will include 80 patients, who will be allocated to the groups A and B, each of which will comprise 40 patients.

The randomisation and stratification shall be carried out according to age (≤64a vs. >64a), sex and side of the infarction.

2.8 Investigational Medicinal product for the Study

The active agent Moxifloxacine will be provided by the company Bayer Vital Ltd, The preparation and packaging of the infusions (Moxifloxacine and placebo) will be carried out by the pharmacy of the Charité (see enclosure 4.6 .).

The necessary labelling, ensuring the blinding for patients, nursing personnel and investigating physicians shall be undertaken by the Institute for Medical Biometry of the Charité.

The therapy shall be routinely applied between 10 o'clock and 12 o'clock.

When a patient is admitted *between Monday and Friday* the requisition shall be submitted to the pharmacy by 15.30, otherwise the therapy shall begin on the following day.

Saturdays: The requisition shall be submitted to the pharmacy by nine o'clock.

Sundays: The requisition must be faxed to the pharmacy on Saturday or Sunday by nine o'clock at the latest and the pharmacist must be pre-warned of the on-call service by telephone.

Weekends: On Saturday morning, an additional bag can be prepared for the Sunday, which shall be applied within 24 hours (must be kept in the hospital ward at room temperature!).

2.9 Dosage, Administering and Duration of Treatment

The treatment shall begin immediately after inclusion and shall last for a standard period of five days.

The treatment shall be conducted as follows:

Group A: patients with severe MCA territory infarction *without* antibacterial prevention. These patients shall receive antibacterial treatment **in accordance with the current standard** as soon as an infection occurs which requires treatment. In the course of the trial, this group shall receive placebos during the **five-day** treatment phase, for reasons of blinding.

Group B: patients with severe MCA territory infarction *with* antibacterial prevention. These patients shall receive, as soon as possible after the event, for the initial **five days** of the treatment phase, an antibacterial **preventive short-term therapy** 1 x tgl. 400 mg of Moxifloxacin (verum, see enclosure 4.16).

The investigational medicinal product shall be infused intravenously over a period of one hour.

2.10 Inclusion Criteria

1. hospitalised patient
2. patient > 18 years of age
3. with an NIH-SS >11 points
4. and/or an ischaemic areal in the cCT (or the cMRT) with ≥ 50% of the MCA flow territory
5. Consent has been given
   a. by the patient or
   b. by the legal representative in the case of patients incapable of giving consent themselves.

2.11 Exclusion Criteria

1. computer tomographic proof of an intra-cerebral haemorrhage or of a lacunary infarction as the probable cause of the current illness
2. participation in a clinical, therapeutical trial within the last 30 days; this shall not include studies that do not contain an intervention and are only descriptive.

3. earlier participation in this trial
4. pregnancy or lactation
5. clinical suspicion or findings of a systemic infection requiring treatment (bacterial, viral, micotic or parasitary) prior to commencing the trial
6. an anamnestic proof of pathogens which are resistant to Moxifloxacin
7. systemic administering of an antibacterial medication within 24 hour's prior to the commencement of the trial
8. the existence of acute or chronic diseases (including immune suppressive diseases), which make it appear doubtful that the patient may safely conclude the trial or give reason to suppose that the evaluation of the patient during the trial may be impeded
9. disorders or circumstances which constitute a counter-indication against the use of Moxifloxacine, in particular
    - **a.** anamnestic or current epileptic seizures
    - **b.** oversensitivity against quinolones in the anamnesis
    - **c.** damage to the sinews resulting from the application of quinolones in the anamnesis or unclear damage to sinews in the anamnesis
    - **d.** prolongation of the QT interval - congenital or documented in the ECG
    - **e.** significant disturbances of the electrolyte balance, in particular
    - **f.** clinically relevant bradycardia
    - **g.** clinically relevant cardiac insufficiency
    - **h.** clinically relevant, symptomatic cardiac arrhythmia, atrial fibrillation, with the exception of
    - **i.** the application of other medicinal products, which are known to lead to a prolongation of the QT interval in the ECG, in particular Class IA and Class III antiarrhythmics
    - **j.** haemodialysis, peritoneal dialysis or plasma pheresis
    - **k.** a state of shock within the last 48 hours or systolic blood pressure <90 mm Hg for more than two hours.
    - **l.** the existence of a haematological or hepatic dysfunction, which manifests itself with
        - i. granulocytes <1/nl
        - ii. thrombocytes <100/nl
        - iii. AST, ALT, whole-bilirubine or alkaline phosphatase >3x upper norm limit
        - iv. and/or which makes treatment with haemodialysis necessary.
10. patients after bone marrow transplantation, organ transplantation or a longer previous treatment (more than seven days) with systemic steroids (equivalent to >25 mg prednisolone/day > 7days) during the last 30 days.

11. patients who have undergone a surgical intervention not longer than three months ago.

2.12 The Documentation of Findings

All findings shall be immediately documented as they are collected on standard CRF forms.

2.12.1 Data Collection Prior to the Commencement Of the Study

The patients shall be questioned and examined by the examining physician. They shall be admitted to the trial if all inclusion criteria are fulfilled and when all exclusion criteria can be negated.

The following data shall be collected prior to and on inclusion in the trial:

1. demographic data
2. anamnesis
3. physical examination
    - a. internistic - including
        - i. blood pressure
        - ii. heart rate
        - iii. breathing rate
        - iv. body temperature
        - v. MOF-S
    - b. neurological - including
        - i. NIH-SS
        - ii. SSS
4. ECG
5. imaging
    - a. thorax x-ray
    - b. computed tomography (cCT) or magnetic resonance tomography (cMRT) of the head
6. Laboratory
    - a. Adre/U/d
    - b. AP/P
    - c. AST/P
    - d. tBil/P
    - e. Cortisol/U/d
    - f. CRP/P
    - g. Diff-BB/B

h. K/P

i. Crea/P

j. Na/P

k. Noradr/U/d

l. UStat/TS

7. Microbiology

a. blood culture

b. rectal smear

8. Quantitative tracheal secretion or bronchoalveolar lavage **AND** blood culture (2x2 Fl.) **AND** urine culture on suspicion of an infection♦

9. Immunology

a. immune status

b. whole blood LPS (TNF)

c. whole blood ConA stimulation

d. PCT

e. T-cell MBP/PLP/TT

f. DC-PCR

2.12.2 Data Collection during the Study

During the first three days of treatment with the trial medication, the continual cardial monitoring shall be guaranteed; on the fourth and fifth days of the treatment ECG readings shall be taken.

During the course of the trial, the following parameters shall be routinely recorded (for a description of the parameters see Annex), for the time schedule for data collection, see trial flow chart):

1. Physical examination

a. internistic - including

vi. blood pressure

vii. heart rate

viii. breathing rate

ix. body temperature

x. MOF-S

b. neurological - including

iii. NIH-SS

iv. SSS

2. Internistic parameters

a. body temperature

   b. blood pressure
   c. heart rate
   d. breathing rate
3. ECG
4. imaging
   a. thorax x-ray
   b. cMRT
5. NIH-SS
6. BI and SIS
7. Laboratory
   a. Adre/U/d
   b. Cortisol/U/d
   c. CRP/P
   d. Diff-BB/B
   e. K/P
   f. Crea/P
   g. Na/P
   h. Noradr/U/d
   i. AP/P
   j. AST/P
   k. tBil/P
8. Microbiology
   c. blood culture
   d. rectal smear
9. Quantitative tracheal secretion or bronchoalveolar Lavage **AND** blood culture (2x2 Fl.) **AND** urine culture on suspicion of an infection♦
10. Immunology
    g. Immune status
    h. whole blood LPS (TNF)
    i. whole blood ConA stimulation
    j. PCT
    k. T-cell MBP/PLP/TT
    l. DC-PCR

2.13 Accompanying Medication

In each case, the patients shall receive the medication which is indicated in the opinion of the physicians. This shall be recorded in the trial documents.

Should medication be initiated with a medicinal product whose application was among the exclusion criteria at the beginning of the trial, the patient must be excluded from the trial. In this case, the decision is made not by the investigating physician, but by the attending physician. If the attending physician is, at the same time, the investigating physician, the decision in question shall be made by the senior physician responsible for the treatment of the patient.

If a patient is diagnosed with a viral all micotic disease after inclusion into the trial, continuation of the trial is permissible provided that a treatment with anti-micotics with topical or systemic effect and/or virostatics is sufficient.

2.14 Complications

All complications of the patient's illness - as well as the illness itself - shall be treated with all the medical means available.

All complications occurring during the entire course of the trial shall be documented as *adverse events*. During this procedure, the documenting physician shall note whether he supposes a relationship with the trial medication and the reasons for this supposition.

Patients who, during the 2nd to the 11th day (*treatment* and *post-treatment* phase), are diagnosed with a complicating infection, such as a pneumonia, an infection of the urinary tract, an endocarditis or sepsis, or a catheter-associated infection (according to the definitions under 4.2), shall receive, **after the collection of material for the immediate determination of pathogens and until specifically targeted treatment is initiated**, a calculated, antibacterial therapy, according to a consensus of recommendations provided by the Institute for Microbiology (SOP). During the second to the sixth day (*treatment* phase), this therapy shall be conducted as an *add-on* therapy in addition to continuing the trial medication.

The current recommendations for the diagnosis of a pneumonia, sepsis, an infection of the urinary tract and a catheter-associated infection are described below. The dosage shall be modified case-by-case.

In the case of a pneumonia:

mild and semi-severe pneumoniae with risk factors, or severe pneumonia with and without risk factors: 3 x 1000 mg Ceftazidim + 5mg/kgKG/d Tobramycin as a one-off dose.

In the case of an infection of the urinary tract:

2 x 500 mg oral (2 x 400 mg i.v.) Ciprofloxacin

In the case of an endocarditis or catheter associated infection or sepsis:

2 x 1000 mg Vancomycin or 1 x 800 mg and during the subsequent days 200 - 400 mg /d as one-off dose Teicoplanin.

The diagnosis of an infection requiring treatment **other than** those listed above shall lead to the de-blinding of the patient for the attending physician.

There is always the possibility of de-blinding the patient if the attending physician - for whatever reason - should deem it necessary. In this case, the decision is made not by the investigating physician, but by the attending physician.

Patients receiving a *therapia minima* shall also be excluded from the trial.

2.15 The Safety of the Study Patients

The safety of the patients shall be ensured by a closely-meshed monitoring system, whereby both clinical as well as paraclinical parameters shall be evaluated. In particular, permanent cardial monitoring shall be guaranteed during the first three days of the treatment.

The trial medication shall be infused intravenously over a period of one hour.

Adverse events shall be followed up from the beginning of the treatment until the end of the trial after a period of six months.

2.16 The Reporting of Serious Adverse Events (SAE)

Serious adverse events shall be reported within 24 hours to the Clinical Examination Management

Dr. G. Arnold,
Neurological Clinic of the Charité
10098 Berlin
Fax 030-450 650 922

and to

Bayer Vital GmbH
Department of Medicin /Medicaments Safety
Dr. Henryk Wroblewski
51368 Leverkusen
Tel. 0214/30-51699
Fax 0214/30-51341

2.17 Interruption of the Study

For individual patients, the trial shall be interrupted if:

1. The patient or his legal representative withdraws consent to his participation in the trial.
2. During the treatment phase, a circumstance arises or later becomes known, which, according to the exclusion criteria, would not permit inclusion in the trial (sea exclusion criteria).
3. an SAE occurs.
4. The investigating physician or the attending physicians are of the opinion that the safety of the patient would be jeopardised by his continued participation in the trial.
5. the attending physicians initiate a *therapia minima.*

The entire trial shall be interrupted if:

1. Approval for the preparation Moxifloxacin should be withdrawn by the authorities.

2. Interruption of the trial should be recommended by the manufacturers of the preparation Moxifloxacin as a result of new findings concerning the safety of themedicinal product.
3. in the course of the trial, more than three cases of SAE should occur which are probably connected with the active agent Moxifloxacin.
4. the trial group should decide for safety reasons, scientific or other reasons to break off the trial.

2.18 Statistical Methods

2.18.1 Primary Aim of the Study

Frequency of the occurrence of a complicating infection up to the 11th day after a large-scale MCA territory infarction.

2.18.2 Secondary Aims of the Study

1. **internistic state** measured with MOF-score on the 11th day after a large-scale MCA territory infarction.
2. **Death** of the patient up to the 11th day, as well as up to three and six months after a large-scale MCA territory infarction.
3. **Neurological deficit** and/or **disability**, measured with the *NIH-Stroke Scale* (NIH-SS), of the *Scandinavian Stroke Scale* (SSS), the *Barthel-Index* (BI) and with the *Stroke Impact Scale* (SIS) on the 11th day, as well as 3 and 6 (±5 d) months after a large-scale MCA territory infarction.
4. the occurrence of a complicating infection♦ up to the 11th day after a large-scale MCA territory infarction.stroke volumetry 3 months (±5) days after a large-scale MCA territory infarction.
5. the occurrence of **pyrexia** up to the 11th day after a large-scale MCA territory infarction.
6. frequency of the prescription of antipyretics up to the 11th day after a large-scale MCA territory infarction.
7. duration of the acute hospital treatment up to the transfer to a rehabilitation establishment.
8. the frequency of the prescription of anti-depressives in the follow-up period, i.e. up to the sixth month (±5 Tage) after a large-scale MCA territory infarction.
9. The frequency of *adverse events*.

2.18.3 Research Design

This shall be in the form of a prospective, controlled, randomised, double-blind trial at two centres..

2.18.4 Hypotheses

The following hypotheses shall be subjected to a *confirmative* examination: An early, preventive, antibacterial short-term therapy lowers the incidence of complicating infections after an MCA territory infarction

Zero hypothesis H0: P(A) = P(B)

Alternative hypothesis (two-sided formula): P(A) = P(B)

(P(A), P(B) percentile proportion of complicating infections in A and/or B).

Moreover, an additional aim of this trial is to conduct an *explorative* examination of further-reaching hypotheses in regard to improving the neurological and internistic success of the treatment. These data shall serve as the basis for considering and planning further, more detailed trials.

2.18.5 Statistical Methods and the Planning of the Scope of the Study

All target values (primary and secondary) shall be subjected to an initial data analysis and descriptive evaluation using exploratory examination methods. In this framework, the structural homogeneity of the treatment groups shall also be examined. The proportion of patients with complicating infections has been selected as the primary endpoint. A possible lowering of the proportion in treatment Arm B against Arm A shall be anaysed with adequate statistical procedures as a difference test via binomial test, χ^2- Test an/or exact Fisher-Test. Secondary endpoints shall be evaluated, according to scaling and distribution type of the observed values with parametric tests or distribution-free counterparts, whereby in the case of small-scale random tests, imbalance or scarcely compiled contingency tables, exact tests shall be used for the examination.

It shall be endeavoured to achieve a *confirmative* verification of Hypothesis 1 (see 1.5). This means that patients (A), treated *post hoc* according to the conventional therapy principle with antibiotics in the case of an infection, for the occurrence of complicating infections (main endpoint see 2.4.1) shall be compared with patients (B), who have received preventive, antibacterial treatment.

On the basis of scientific literature and our own retrospective trial, we shall assume a rate of complicating infections of 40% for Group A, whereas we presuppose an infection rate of 10% at the most for Group B.

Both complication rates are conservatively estimated. In our retrospective review of severe stroke cases, the spontaneous infection rate was higher. For the infection rate under treatment with the broadband antibiotic Moxifloxacin, on the other hand, there are no data available.

On the basis of the presupposed numerical data, the verification of Hypothesis 1, applying a power of 80%, the minimum size of Groups A and B may be determined at 32 patients for each group respectively.

Power Analysis for Proportions

Results with HA: P1 ≠ P2 (two-sided)

**Power**	**N1**	**N2**	**P1**	**P2**	**Alpha**	**Beta**
0.8064	32	32	0.4000	0.1000	0.0500	0.1936

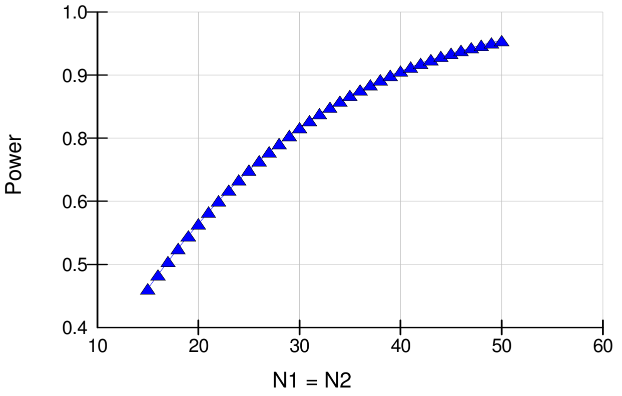

Graph: Relationship between Power and Number of Cases

2.18.6 The Definition of Evaluation Collectives

The analysis in regard to the main objectives criterion (primary endpoint) shall be conducted under consideration of all patients who have completed the treatment with the investigational medicinal product or have dropped out as a result of reaching the primary endpoint (*per protocol*); they must have been clinically assessed on the 11th day after The event. For the *intention-to-treat-analysis* all patients shall be taken into consideration who have received at least one treatment with the trial therapy.

For therapy failures, whose attending physicians, on reaching the primary endpoint during the treatment phase, they naturally have to be de-blinded for the trial medication, it shall, in any case, be ensured that the assessment in regard to the secondary endpoint in the follow-up of the trial shall be conducted by investigating physicians who have not been de-blinded, in order to enable a *per-protocol* analysis also for these patients.

2.18.7 Randomisation

Prior to admitting a patient to the protocol, the completed randomisation form (filled in with inclusion and exclusion criteria, initials, sex, date of birth) must be submitted or sent by fax to the Central Office of the trial. There, the randomisation and allocation of randomisation numbers, which are to be noted on the randomisation form, is carried out. The randomisation form shall be presented or sent by fax to the investigating physician responsible for inclusion. In the same way, the number of the corresponding patient's Case Report File (CRF) shall be notified to the investigating physician responsible for the inclusion.

2.18.8 Stratification and De-Blinding

The trial provides for a stratification according to the following criteria:

- sex (male/female)

- side affected by the cerebral infarction (right/left)
- Age of the patients (≤64 years/>64 years)

The frequently used randomisation in blocks, with numerous stratification dimensions or rare factor characteristics, has great disadvantages, since it can become problematic to achieve a balanced enrollment of the therapy arms. For this reason an adaptive randomisation shall be applied, for which we shall use a special computer programme (S-Plus) which works in correspondence with the envisaged database (MS Access) and guarantees a balanced enrolment of patients in the therapy arms, even when some individual strata remain vacant.

Sealed Emergency Letters in duplicate shall be prepared. One set of the emergency letters shall be sent out respectively as follows

- together with the trial medication to the investigating physician
- to the Head of the Clinical Examination Management

In those cases in which it is important to know to which treatment group a patient belongs in order to make a medical decision, de-blinding in individual cases may take place. To this end, a separate detailed documentation shall be compiled, which must include the reasons for breaking the test code.

2.18.9 Quality Assurance

The clinical trial shall be conducted according to the principles governing the proper execution of the clinical testing of medicinal products (Federal Gazette 1987, 243), according to EG-GCP directives.

For the purposes of quality assurance, a monitoring procedure shall be carried out. The monitoring procedure to be set up by the Central Administration Office of the trial shall comprise an initial medical examination and further medical examinations in the follow up phase and shall include a final medical examination, in which the documentation forms shall be reviewed and examined in regard to completeness and plausibility by comparison with the original patient records (Source Documentation Verification). During the entire test period, the participating hospital care units shall be monitored by telephone (telephone monitoring). Finally, the database of the trial shall be programmed according to GCP with a Log Book, so that an on-going monitoring of the data as well as the reporting deadlines is carried out automatically.

The quality of the trial data shall be ensured by setting up monitoring procedures for the data management in the form of Standard Operating Procedures (SOP) according to the EG recommendations for Good Clinical Practice (GCP).

Quality indicators to be used in the course of the trial comprise adherence to the selection criteria, compliance with the randomisation principle, adherence to the protocol in regard to treatment, as well as adherence to the trial and evaluation deadlines. In the course of a half-yearly intermediate evaluation, an examination and evaluation shall be carried out in respect of these criteria.

2.18.10 Data Management

The documentation as well as the data management and the data control, including data security, in compliance with data protection regulations, shall be organised and

carried out by the Central Administration Office of the trial. The volume of the data and the envisaged duration of the trial make it necessary, to support both the planned trial monitoring as well as the biometric evaluation by means of a common database. This shall ensure the prompt collection and evaluation of trial data as well as a continuous control of the data in respect of completeness and correctness. Furthermore, with the help of the database it is also possible, from the very beginning, to perform an accompanying, exploratory data analysis during the execution of the trial. The basic prerequisite for the reliable collection and statistical evaluation of the data is that the investigating physicians continually send the documentation forms, completely filled out, without any delays, to the Central Administration Office.

The compilation of the database, including the implementation of the randomisation and stratification algorithms, shall be guaranteed by the Institute for Medical Biometry of the Charité.

2.18.10.1 Study Secretary

The Study Secretary shall be available in the Central Administration Office for organisational questions and problems in connection with the documentation as well as the data management of the trial.

The Study Secretary shall provide the link to the Head of the Clinical Examination and to the Scientific Project Management.

2.18.10.2 Patient Identification List

All patient-related data shall be collected in pseudonymised form, in which each patient shall be unequivocably identified by a code number, which shall be allocated on registration, based on his initials, date of birth and sex. The investigating physician shall keep a confidential patient list, in which the code data are related to the patients full name.

2.18.10.3 Data Collection/Documentation Forms

The collected findings, measurement results, concomittant phenomena and all data collected in accordance with the trial schedule shall be entered on the trial report forms. Each individual trial report form must be properly signed and dated by the physician. The original is intended for the Central Administration Office, one copy shall be kept by the investigating physician. The forms shall be completed in ink or by ballpoint pen, pencil entries are not permissible. Corrections are to be made in the following manner: The erroneous entry shall be crossed out by a single line, the correct information shall be entered beside it and shall be initialled and dated by the investigating physician and, if necessary, the reasons for the correction shall be given. Data boxes which cannot be filled out because of missing information shall be annotated. The forms shall be filled out promptly and subsequently checked by the investigating physician, they shall be duly dated and signed and then sent to the Central Administration Office.

The patients' case report files (CRF) shall be kept together during the entire trial in the respective hospital care unit, i.e. the removal of individual trial report forms from the file is neither permissible nor shall it be possible. The number and sequence of the planned trials can be seen from the research design and/or from the CRF. The sending of patients' data to the Central Administration Office (in the form of copies of each

respective form in the CRF) shall be carried out continuously, but at least once per month per patient.

2.18.10.4 Patient Case Report File (CRF)

Each form of the CRF contains the allocated randomisation number, the date of birth and the initials of the patient. The ring folder additionally contains a planning sheet, on which the trial dates are entered - scheduled in relation to the date of inclusion. The patient shall keep his randomisation number during the entire period of the trial. Numbers shall be neither deleted nor added to the randomisation numbers.

2.18.10.5 Data Processing

In the Central Administration Office, the data shall be electronically recorded in pseudonymised form. The verification of the correctness of the data shall be carried out by range, validity and consistency checks. Non-plausible or missing data can be corrected or complemented in consultation with the investigating physician. Documents of proof of corrections shall be kept together with the trial report forms.

The validated data shall be stored in a suitable database. At the end of the trial, after completing all entries, the database shall be closed. This procedure shall be documented. For evaluation purposes, a suitable software shall be used, which shall be provided by the Institute for Medical Biometry of the Charité.

The evaluation shall be carried out under the supervision of the Institute for Medical Biometry of the Charité.

2.18.10.6 The Safekeeping of the Study Records

The originals of all central trial documents, including documentation forms, shall remain in the custody of the Central Administration office for at least 15 years after the compilation of the final report.

The chief investigating physician shall hold in safe custody all pertinent administrative documents (correspondence with the Ethics Commission, Supervisory Authority, Study Management, Central Administration office), the Patients Identification List, the signed declarations of consent, copies of the documentation forms and the general trial documentation (protocol, amendments) likewise for the above-mentioned period of time.

Original data pertaining to the trial patients (health records) shall be kept in accordance with the mandatory archive period applicable to research centres, but in any case for not less than 15 years.

2.19 Ethics

2.19.1 The Declaration of Helsinki

Phase IIb of the clinical trial shall be conducted in conformity with The Declaration of the World Medical Association in Helsinki on ethical principles for medical research on humans, in the version approved by the 52nd General Assembly of the World Medical Association in Edinburgh, Scotland in October 2000.

In particular, for those infarction patients whose capacity to express consent is limited, the procedure according to article 22 to 26 of the Declaration of Helsinki shall be strictly adhered to.

2.19.2 Ethics Commission

The research protocol, patients information sheets and declarations of consent shall be submitted for appraisal to the ethics commission (ethics commission of the Charité, Campus Charité Mitte, 10098 Berlin).

The ethics commission shall be informed, without delay, by the chief investigating physician concerning all amendments to the research protocol which could jeopardise the safety of patients. Furthermore, the commission shall be informed about all serious adverse events which are reported as well as concerning the regular or premature conclusion of the trial.

After the incorporation of a further centre, the ethics commission responsible there shall be consulted before patients are admitted to the trial. It is further required to await the verdict of this commission and likewise to inform this commission of any changes to the protocol, adverse events and the conclusion of the trial.

2.19.3 Informing the Patients

Prior to being admitted to the trial (randomisation), every patient or his legal representative shall be informed by the attending physician concerning the nature, aims expected advantages and possible risks of the trial.

2.19.4 Consent to Participation in the Study

Every patient or his legal representative must submit written consent to his participation in the trial prior to his admission. The patient shall be afforded sufficient time and opportunity to reach his decision and to clarify any open questions prior to the initiation of trial measures.

The declaration of consent shall be signed by the patient or his legal representative and by the informing physician. Should the patient be incapable of signing the declaration himself, the verbal information of the patient shall be confirmed by the signature of a witness. Samples of patient information and of the declaration of consent are included in the Annex. A written copy of the wording of the patient information and the declaration of consent shall be handed out to the patient.

2.19.5 The Use, Storage and Communication of Data

The patients shall be informed that the data relating to their illness will be stored in pseudonymised form and used for the purpose of scientific evaluations (publication, admission dossiers) in anonymised form. The patients shall be entitled to be informed about the data stored.

2.19.6 Amendments to the Protocol

Should it become necessary to make amendments to the protocol, these shall only be carried out in consultation with the Central Administration Office and shall be documented by the Study Secretary. The written amendments must be reviewed by the investigating physicians and shall be submitted to the ethics commission. In the event

that the amendments change the fundamental basis of the research design, a new declaration of consent must be prepared and signed once again by the patients included in the trial.

2.20 Legal and Administrative Regulations

2.20.1 GCP, Fundamental Legal Principles

The recommendations of Good Clinical Practice (GCP), valid since the 17th January 1997, shall be observed.

The principles governing the proper execution of clinical tests on medicinal products (German Federal Gazette No. 243, dated 30.12.1987), the regulations of the German Medicinal Products Act (AMG) 1976, last amended 1998 and the guidelines for testing medicinal products (1999) as well as the X-Ray Directive and the Data Protection Act shall be adhered to.

The investigating physicians possess many years of experience in the clinical testing of medicinal products.

2.20.2 Patients Insurance

For the envisaged clinical trial, the Charité shall take out an insurance policy according to the relevant legal regulations.

2.20.3 Funding

This trial shall be conducted on the initiative of the Neurological Clinic and shall be led by physicians of the Charité. Further physicians from a second neurological recruiting centre shall be included in the research group.

The trial shall be funded largely by the Charité, the Hermann-and-Lilly-Schillung FoANDation, as well as by the German Research FoANDation and to a smaller extent by third-party funds, provided by the company BayerVital, 51368 Leverkusen.

2.20.4 Final Report and Publication

After completion of the biometric evaluation, an integrated report shall be compiled and published. The report shall include the clinical report, the statistical report, individual evaluation tables and final conclusions.

The publication of the trial findings shall be carried out irrespective of the results obtained.

2.20.4.1 Adherence to the Protocol and the Protocol Amendments

The research protocol shall be precisely adhered to. Every deviation from the planned trial and treatment measures or timings under the responsibility of the investigating physician shall be documented and the reasons given (e.g. emergency measures). Amendments or extensions to the research protocol may only be initiated and authorised by members of the research group and shall be documented as *amendments*.

2.21 Study Flowchart

	pre-treat.	treatment					post-treatment			follow-up	
Visite	V1	V2	V3	V4	V5	V6	V7	V8	V9	V10	V11
**Day/Month**	**1*)**	**2**	**3**	**4**	**5**	**6**	**7**	**9**	**11**	**3 M**	**6 M**
**demografic data**	X										
**Anamnesis/Catamnesis**	X									X	X
**physical examination**	X	X	X	X	X	X	X	X	X		
**internististic monitoring**											
HF	X	X^{6h})	X^{6h})	X^{6h})	X^{6h})	X^{6h})	X	X	X		
ECG	X	X^{m})	X^{m})	X^{m})	X	X					
Temp.	X	X^{2h})	X^{2h})	X^{2h})	X^{2h})	X^{2h})	X	X	X		
AF	X	X^{6h})	X^{6h})	X^{6h})	X^{6h})	X^{6h})	X	X	X		
RR	X	X^{6h})	X^{6h})	X^{6h})	X^{6h})	X^{6h})	X	X	X		
MOF-S	X	X	X	X	X	X	X	X	X		
**neurological monitoring**											
NIH-SS	X	X				X			X	X	X
SSS	X	X				X			X	X	X
BI									X	X	X
SIS									X	X	X
MADRS									X	X	X
**Imaging**											
Rö-Thorax	X										
cCT/cMRT	X		X							X	
**Laboratory Testing**											
AP/P	X										
AST/P	X										
tBil/P	X										
Crea/P	X										
Na/P	X	X	X	X	X	X					
K/P	X	X	X	X	X	X					
Gluc/P	X	X	X	X	X	X	X	X	X		
CRP/P	X	X	X	X	X	X	X	X	X		
Diff-BB/B	X	X		X	X	X	X		X		
UStat/TS	X										
Cortisol/U/d	X	X	X					X			
Noradr/U/d	X	X	X					X			
**Mikrobiology**											
quantitative tracheal secretion or bronchoalveolar lavage		on infection					on infection				
blood culture(2x2FL)		on infection					on infection				
urine culture		on infection					on infection				
throat swab	X							X			
rectal smear	X							X			
stool sample	X							X			
**Immunology**											
clinical immune status	X		X					X			X
whole blood LPS (TNF)	X		X					X		X	X
whole blood ConABD	X		X					X		X	X
PCT	X		X					X		X	X
T-cell stimulation MBP/PLP/TT	X							X			X
T-cell stimulation PHA	X							X			X
DC-PCR	X							X			X
gene-expression-profile	X							X			X
**continual monitoring**	X	X	X	X	X	X	X	X	X	X	X
**inclusion and exclusion criteria**	X										
**declaration of consent**	X										
**adverse events monitoring**	X	X	X	X	X	X	X	X	X	X	X

*) Day 1 beginns as per definition <36hrs. after the event

^{2hrs}) daily at 2-hr. intervals

^{6hrs}) daily at 6-hr. intervals

^{m}) besides daily paper record - continual monitoring

3 Literature

4 Enclosure:

4.1 Clinical Evaluation Scales

4.1.1 NIH Stroke Scale

Instructions	Scale Definition
**1a. Level of Consciousness:** The investigator must choose a response if a full evaluation is prevented by such obstacles as an endotracheal tube, language barrier, orotracheal trauma/bandages. A 3 is scored only if the patient makes no movement (other than reflexive posturing) in response to noxious stimulation.	0 = **Alert;** keenly responsive.<br>1 = **Not alert**; but arousable by minor stimulation to obey, answer, or respond.<br>2 = **Not alert**; requires repeated stimulation to attend, or is obtunded and requires strong or painful stimulation to make movements (not stereotyped).<br>3 = Responds only with reflex motor or autonomic effects or totally unresponsive, flaccid, and areflexic.
**1b. LOC Questions:** The patient is asked the month and his/her age. The answer must be correct - there is no partial credit for being close. Aphasic and stuporous patients who do not comprehend the questions will score 2. Patients unable to speak because of endotracheal intubation, orotracheal trauma, severe dysarthria from any cause, language barrier, or any other problem not secondary to aphasia are given a 1. It is important that only the initial answer be graded and that the examiner not "help" the patient with verbal or non-verbal cues.	0 = **Answers** both questions correctly.<br>1 = **Answers** one question correctly.<br>2 = **Answers** neither question correctly.
**1c. LOC Commands:** The patient is asked to open and close the eyes and then to grip and release the non-paretic hand. Substitute another one step command if the hands cannot be used. Credit is given if an unequivocal attempt is made but not completed due to weakness. If the patient does not respond to command, the task should be demonstrated to him or her (pantomime), and the result scored (i.e., follows none, one or two commands). Patients with trauma, amputation, or other physical impediments should be given suitable one-step commands. Only the first attempt is scored.	0 = **Performs** both tasks correctly.<br>1 = **Performs** one task correctly.<br>2 = **Performs** neither task correctly.
**2. Best Gaze:** Only horizontal eye movements will be tested. Voluntary or reflexive (oculocephalic) eye movements will be scored, but caloric testing is not done. If the patient has a conjugate deviation of the eyes that can be overcome by voluntary or reflexive activity, the score will be 1. If a patient has an isolated peripheral nerve paresis (CN III, IV or VI), score a 1. Gaze is testable in all aphasic patients. Patients with ocular trauma, bandages, pre-existing blindness, or other disorder of visual acuity or fields should be tested with reflexive movements, and a choice made by the investigator. Establishing eye contact and then moving about the patient from side to side will occasionally clarify the presence of a partial gaze palsy.	0 = **Normal.**<br>1 = **Partial gaze palsy;** gaze is abnormal in one or both eyes, but forced deviation or total gaze paresis is not present.<br>2 = **Forced deviation,** or total gaze paresis not overcome by the oculocephalic maneuver.

**3. Visual:** Visual fields (upper and lower quadrants) are tested by confrontation, using finger counting or visual threat, as appropriate. Patients may be encouraged, but if they look at the side of the moving fingers appropriately, this can be scored as normal. If there is unilateral blindness or enucleation, visual fields in the remaining eye are scored. Score 1 only if a clear-cut asymmetry, including quadrantanopia, is found. If patient is blind from any cause, score 3. Double simultaneous stimulation is performed at this point. If there is extinction, patient receives a 1, and the results are used to respond to item 11.	0 = **No visual loss.**<br>1 = **Partial hemianopia.**<br>2 = **Complete hemianopia.**<br>3 = **Bilateral hemianopia** (blind including cortical blindness).	____
**4. Facial Palsy:** Ask – or use pantomime to encourage – the patient to show teeth or raise eyebrows and close eyes. Score symmetry of grimace in response to noxious stimuli in the poorly responsive or non-comprehending patient. If facial trauma/bandages, orotracheal tube, tape or other physical barriers obscure the face, these should be removed to the extent possible.	0 = **Normal** symmetrical movements.<br>1 = **Minor paralysis** (flattened nasolabial fold, asymmetry on smiling).<br>2 = **Partial paralysis** (total or near-total paralysis of lower face).<br>3 = **Complete paralysis** of one or both sides (absence of facial movement in the upper and lower face).	____
**5. Motor Arm:** The limb is placed in the appropriate position: extend the arms (palms down) 90 degrees (if sitting) or 45 degrees (if supine). Drift is scored if the arm falls before 10 seconds. The aphasic patient is encouraged using urgency in the voice and pantomime, but not noxious stimulation. Each limb is tested in turn, beginning with the non-paretic arm. Only in the case of amputation or joint fusion at the shoulder, the examiner should record the score as untestable (UN), and clearly write the explanation for this choice.	0 = **No drift;** limb holds 90 (or 45) degrees for full 10 seconds.<br>1 = **Drift;** limb holds 90 (or 45) degrees, but drifts down before full 10 seconds; does not hit bed or other support.<br>2 = **Some effort against gravity;** limb cannot get to or maintain (if cued) 90 (or 45) degrees, drifts down to bed, but has some effort against gravity.<br>3 = **No effort against gravity;** limb falls.<br>4 = **No movement.**<br>UN = **Amputation** or joint fusion, explain: ____________________<br><br>**5a. Left Arm**<br><br>**5b. Right Arm**	 <br><br><br><br><br><br><br>____<br><br>____
**6. Motor Leg:** The limb is placed in the appropriate position: hold the leg at 30 degrees (always tested supine). Drift is scored if the leg falls before 5 seconds. The aphasic patient is encouraged using urgency in the voice and pantomime, but not noxious stimulation. Each limb is tested in turn, beginning with the non-paretic leg. Only in the case of amputation or joint fusion at the hip, the examiner should record the score as untestable (UN), and clearly write the explanation for this choice.	0 = **No drift;** leg holds 30-degree position for full 5 seconds.<br>1 = **Drift;** leg falls by the end of the 5-second period but does not hit bed.<br>2 = **Some effort against gravity;** leg falls to bed by 5 seconds, but has some effort against gravity.<br>3 = **No effort against gravity;** leg falls to bed immediately.<br>4 = **No movement.**<br>UN = **Amputation** or joint fusion, explain: _______________<br><br>**6a. Left Leg**<br><br>**6b. Right Leg**	____

**7. Limb Ataxia:** This item is aimed at finding evidence of a unilateral cerebellar lesion. Test with eyes open. In case of visual defect, ensure testing is done in intact visual field. The finger-nose-finger and heel-shin tests are performed on both sides, and ataxia is scored only if present out of proportion to weakness. Ataxia is absent in the patient who cannot understand or is paralyzed. Only in the case of amputation or joint fusion, the examiner should record the score as untestable (UN), and clearly write the explanation for this choice. In case of blindness, test by having the patient touch nose from extended arm position.	0 = **Absent.**<br>1 = **Present in one limb.**<br>2 = **Present in two limbs.**<br>UN = **Amputation** or joint fusion, explain: ______________	___
**8. Sensory:** Sensation or grimace to pinprick when tested, or withdrawal from noxious stimulus in the obtunded or aphasic patient. Only sensory loss attributed to stroke is scored as abnormal and the examiner should test as many body areas (arms [not hands], legs, trunk, face) as needed to accurately check for hemisensory loss. A score of 2, "severe or total sensory loss," should only be given when a severe or total loss of sensation can be clearly demonstrated. Stuporous and aphasic patients will, therefore, probably score 1 or 0. The patient with brainstem stroke who has bilateral loss of sensation is scored 2. If the patient does not respond and is quadriplegic, score 2. Patients in a coma (item 1a=3) are automatically given a 2 on this item.	0 = **Normal;** no sensory loss.<br>1 = **Mild-to-moderate sensory loss;** patient feels pinprick is less sharp or is dull on the affected side; or there is a loss of superficial pain with pinprick, but patient is aware of being touched.<br>2 = **Severe to total sensory loss;** patient is not aware of being touched in the face, arm, and leg.	___
**9. Best Language:** A great deal of information about comprehension will be obtained during the preceding sections of the examination. For this scale item, the patient is asked to describe what is happening in the attached picture, to name the items on the attached naming sheet and to read from the attached list of sentences. Comprehension is judged from responses here, as well as to all of the commands in the preceding general neurological exam. If visual loss interferes with the tests, ask the patient to identify objects placed in the hand, repeat, and produce speech. The intubated patient should be asked to write. The patient in a coma (item 1a=3) will automatically score 3 on this item. The examiner must choose a score for the patient with stupor or limited cooperation, but a score of 3 should be used only if the patient is mute and follows no one-step commands.	0 = **No aphasia;** normal.<br>1 = **Mild-to-moderate aphasia;** some obvious loss of fluency or facility of comprehension, without significant limitation on ideas expressed or form of expression. Reduction of speech and/or comprehension, however, makes conversation about provided materials difficult or impossible. For example, in conversation about provided materials, examiner can identify picture or naming card content from patient's response.<br>2 = **Severe aphasia;** all communication is through fragmentary expression; great need for inference, questioning, and guessing by the listener. Range of information that can be exchanged is limited; listener carries burden of communication. Examiner cannot identify materials provided from patient response.<br>3 = **Mute, global aphasia;** no usable speech or auditory comprehension.	___
**10. Dysarthria:** If patient is thought to be normal, an adequate sample of speech must be obtained by asking patient to read or repeat words from the attached list. If the patient has severe aphasia, the clarity of articulation of spontaneous speech can be rated. Only if the patient is intubated or has other physical barriers to producing speech, the examiner should record the score as untestable (UN), and clearly write an explanation for this choice. Do not tell the patient why he or she is being tested.	0 = **Normal.**<br>1 = **Mild-to-moderate dysarthria;** patient slurs at least some words and, at worst, can be understood with some difficulty.<br>2 = **Severe dysarthria;** patient's speech is so slurred as to be unintelligible in the absence of or out of proportion to any dysphasia, or is mute/anarthric.<br>UN = **Intubated** or other physical barrier, explain:__________________________	___

**11. Extinction and Inattention (formerly Neglect):** Sufficient information to identify neglect may be obtained during the prior testing. If the patient has a severe visual loss preventing visual double simultaneous stimulation, and the cutaneous stimuli are normal, the score is normal. If the patient has aphasia but does appear to attend to both sides, the score is normal. The presence of visual spatial neglect or anosagnosia may also be taken as evidence of abnormality. Since the abnormality is scored only if present, the item is never untestable.	0 = **No abnormality.**<br>1 = **Visual, tactile, auditory, spatial, or personal inattention** or extinction to bilateral simultaneous stimulation in one of the sensory modalities.<br>2 = **Profound hemi-inattention or extinction to more than one modality;** does not recognize own hand or orients to only one side of space.	___

4.1.2 Scandinavian Stroke Scale (SSS)

Function	Score	Prognostic Score	Long Term Score
**Consciousness**:			
-fully conscious	**6**	____	
-somnolent, can be awaked to full consciousness	**4**		
-reacts to verbal command, but is not fully conscious	**2**		
**Eye movement**:			
-no gaze palsy	**4**	____	
-gaze palsy present	**2**		
-conjugate eye deviation	**0**		
**Arm, motor power *:**			
-raises arm with normal strength	**6**	____	____
-raises arm with reduced strength	**5**		
-raises arm with flexion in elbow	**4**		
-can move, but not against gravity	**2**		
-paralysis	**0**		
**Hand, motor power *:**			
-normal strength	**6**		____
-reduced strength in full range	**4**		
-some movement, fingertips do not reach palm	**2**		
-paralysis	**0**		
**Leg, motor power *:**			
-normal strength	**6**	____	____
-raises straight leg with reduced strength	**5**		
-raises leg with flexion of knee	**4**		
-can move, but not against gravity	**2**		
-paralysis	**0**		

**Orientation**:			
-correct for time, place and person	**6**		____
-two of these	**4**		
-one of these	**2**		
-completely disorientated	**0**		
**Speech**:			
-no aphasia	**10**		____
-limited vocabulary or incoherent speech	**6**		
-more than yes/no, but not longer sentences	**3**		
-only yes/no or less	**0**		
**Facial palsy**:			
-none/dubious	**2**		____
-present	**0**		
**Gait**:			
-walks 5 m without aids	**12**		____
-walks with aids	**9**		
-walks with help of another person	**6**		
-sits without support	**3**		
-bedridden/wheelchair	**0**		
**Maximal Score**	____	**22**	**48**

* Motor power is assessed only on the affected side.

4.1.3 Barthel Index

Activity	Score
**FEEDING**	
0 = unable	
5 = needs help cutting, spreading butter, etc., or requires modified diet	
10 = independent	_____
**BATHING**	
0 = dependent	
5 = independent (or in shower)	_____
**GROOMING**	
0 = needs to help with personal care	
5 = independent face/hair/teeth/shaving (implements provided)	_____
**DRESSING**	
0 = dependent	
5 = needs help but can do about half unaided	
10 = independent (including buttons, zips, laces, etc.)	_____
**BOWELS**	
0 = incontinent (or needs to be given enemas)	
5 = occasional accident	
10 = continent	_____
**BLADDER**	
0 = incontinent, or catheterized and unable to manage alone	
5 = occasional accident	
10 = continent	_____
**TOILET USE**	
0 = dependent	
5 = needs some help, but can do something alone	
10 = independent (on and off, dressing, wiping)	_____
**TRANSFERS (BED TO CHAIR AND BACK)**	
0 = unable, no sitting balance	
5 = major help (one or two people, physical), can sit	
10 = minor help (verbal or physical)	
15 = independent	_____
**MOBILITY (ON LEVEL SURFACES)**	
0 = immobile or < 50 yards	
5 = wheelchair independent, including corners, > 50 yards	
10 = walks with help of one person (verbal or physical) > 50 yards	
15 = independent (but may use any aid; for example, stick) > 50 yards	_____
**STAIRS**	
0 = unable	
5 = needs help (verbal, physical, carrying aid)	
10 = independent	_____
**TOTAL (0–100):**	_____

4.1.4 Stroke Impact Scale A

These questions are about the physical problems which may have occurred as a result of your stroke.

**1. In the past week, how would you rate the strength of your....**	**A lot of strength**	**Quite a bit of strength**	**Some strength**	**A little strength**	**No strength at all**
a. Arm that was most affected by your stroke?	5	4	3	2	1
b. Grip of your hand that was most affected by your stroke?	5	4	3	2	1
c. Leg that was most affected by your stroke?	5	4	3	2	1
d. Foot/ankle that was most affected by your stroke?	5	4	3	2	1

These questions are about your memory and thinking.

**2. In the past week, how difficult was it for you to...**	**Not difficult at all**	**A little difficult**	**Somewhat difficult**	**Very difficult**	**Extremely difficult**
a. Remember things that people just told you?	5	4	3	2	1
b. Remember things that happened the day before?	5	4	3	2	1
c. Remember to do things (e.g. keep scheduled appointments or take medication)?	5	4	3	2	1
d. Remember the day of the week?	5	4	3	2	1
e. Concentrate?	5	4	3	2	1
f. Think quickly?	5	4	3	2	1
g. Solve everyday problems?	5	4	3	2	1

Stroke Impact Scale B

These questions are about how you feel, about changes in your mood and about your ability to control your emotions since your stroke.

**3. In the past week, how often did you...**	**None of the time**	**A little of the time**	**Some of the time**	**Most of the time**	**All of the time**
a. Feel sad?	5	4	3	2	1
b. Feel that there is nobody you are close to?	5	4	3	2	1
c. Feel that you are a burden to others?	5	4	3	2	1
d. Feel that you have nothing to look forward to?	5	4	3	2	1
e. Blame yourself for mistakes that you made?	5	4	3	2	1
f. Enjoy things as much as ever?	5	4	3	2	1
g. Feel quite nervous?	5	4	3	2	1
h. Feel that life is worth living?	5	4	3	2	1
i. Smile and laugh at least once a day?	5	4	3	2	1

The following questions are about your ability to communicate with other people, as well as your ability to understand what you read and what you hear in a conversation.

**4. In the past week, how difficult was it to...**	**Not difficult at all**	**A little difficult**	**Somewhat difficult**	**Very difficult**	**Extremely difficult**
a. Say the name of someone who was in front of you?	5	4	3	2	1
b. Understand what was being said to you in a conversation?	5	4	3	2	1
c. Reply to questions?	5	4	3	2	1
d. Correctly name objects?	5	4	3	2	1
e. Participate in a conversation with a group of people?	5	4	3	2	1
f. Have a conversation on the telephone?	5	4	3	2	1
g. Call another person on the telephone, including selecting the correct phone number and dialing?	5	4	3	2	1

Stroke Impact Scale C

The following questions ask about activities you might do during a typical day.

5. In the past 2 weeks, how difficult was it to...	Not difficult at all	A little difficult	Somewhat difficult	Very difficult	Could not do at all
a. Cut your food with a knife and fork?	5	4	3	2	1
b. Dress the top part of your body?	5	4	3	2	1
c. Bathe yourself?	5	4	3	2	1
d. Clip your toenails?	5	4	3	2	1
e. Get to the toilet on time?	5	4	3	2	1
f. Control your bladder (not have an accident)?	5	4	3	2	1
g. Control your bowels (not have an accident)?	5	4	3	2	1
h. Do light household tasks/chores (e.g. dust, make a bed, take out garbage, do the dishes)?	5	4	3	2	1
i. Go shopping?	5	4	3	2	1
j. Do heavy household chores (e.g. vacuum, laundry or yard work)?	5	4	3	2	1

The following questions are about your ability to be mobile, at home and in the community.

6. In the past 2 weeks, how difficult was it to...	Not difficult at all	A little difficult	Somewhat difficult	Very difficult	Could not do at all
a. Stay sitting without losing your balance?	5	4	3	2	1
b. Stay standing without losing your balance?	5	4	3	2	1
c. Walk without losing your balance?	5	4	3	2	1
d. Move from a bed to a chair?	5	4	3	2	1
e. Walk one block?	5	4	3	2	1
f. Walk fast?	5	4	3	2	1
g. Climb one flight of stairs?	5	4	3	2	1
h. Climb several flights of stairs?	5	4	3	2	1
i. Get in and out of a car?	5	4	3	2	1

Stroke Impact Scale D

The following questions are about your ability to use your hand that was MOST AFFECTED by your stroke.

**7. In the past 2 weeks, how difficult was it to use your hand that was most affected by your stroke to...**	**Not difficult at all**	**A little difficult**	**Somewhat difficult**	**Very difficult**	**Could not do at all**
a. Carry heavy objects (e.g. bag of groceries)?	5	4	3	2	1
b. Turn a doorknob?	5	4	3	2	1
c. Open a can or jar?	5	4	3	2	1
d. Tie a shoe lace?	5	4	3	2	1
e. Pick up a dime?	5	4	3	2	1

The following questions are about how stroke has affected your ability to participate in the activities that you usually do, things that are meaningful to you and help you to find purpose in life.

**8. During the past 4 weeks, how much of the time have you been limited in...**	**None of the time**	**A little of the time**	**Some of the time**	**Most of the time**	**All of the time**
a. Your work (paid, voluntary or other)	5	4	3	2	1
b. Your social activities?	5	4	3	2	1
c. Quiet recreation (crafts, reading)?	5	4	3	2	1
d. Active recreation (sports, outings, travel)?	5	4	3	2	1
e. Your role as a family member and/or friend?	5	4	3	2	1
f. Your participation in spiritual or religious activities?	5	4	3	2	1
g. Your ability to control your life as you wish?	5	4	3	2	1
h. Your ability to help others?	5	4	3	2	1

Stroke Impact Scale E

9. Stroke Recovery

On a scale of 0 to 100, with 100 representing full recovery and 0 representing no recovery, how much have you recovered from your stroke?

_____ 100 Full Recovery
—
_____ 90
—
_____ 80
—
_____ 70
—
_____ 60
—
_____ 50
—
_____ 40
—
_____ 30
—
_____ 20
—
_____ 10

_______ 0 No Recovery

4.1.5 Montgomery Asberg Depression Rating Scale (MADRS)

**1. Apparent sadness**<br>Representing despondency, gloom and despair (more than just ordinary transient low spirits), reflected in speech, facial expression, and posture. Rate by depth and inability to brighten up.	
**0** = No sadness.	☐
**2** = Looks dispirited but does brighten up without difficulty.	☐
**4** = Appears sad and unhappy most of the time.	☐
**6** = Looks miserable all the time. Extremely despondent	☐

**2. Reported sadness**<br>Representing reports of depressed mood, regardless of whether it is reflected in appearance or not. Includes low spirits, despondency or the feeling of being beyond help and without hope.	
**0** = Occasional sadness in keeping with the circumstances.	☐
**2** = Sad or low but brightens up without difficulty.	☐
**4** = Pervasive feelings of sadness or gloominess. The mood is still influenced by external circumstances.	☐
**6** = Continuous or unvarying sadness, misery or despondency.	☐

**3. Inner tension**<br>Representing feelings of ill-defined discomfort, edginess, inner turmoil, mental tension mounting to either panic, dread or anguish. Rate according to intensity, frequency, duration and the extent of reassurance called for.	
0 = Placid. Only fleeting inner tension.	☐
2 = Occasional feelings of edginess and ill-defined discomfort.	☐
4 = Continuous feelings of inner tension or intermittent panic which the patient can only master with some difficulty.	☐
**6** = Unrelenting dread or anguish. Overwhelming panic.	☐

**4. Reduced sleep**<br>Representing the experience of reduced duration or depth of sleep compared to the subject's own normal pattern when well.	
**0** = Sleeps as normal.	☐
**2** = Slight difficulty dropping off to sleep or slightly reduced, light or fitful sleep.	☐
**4** = Moderate stiffness and resistance	☐
**6** = Sleep reduced or broken by at least 2 hours.	☐

**5. Reduced appetite**<br>Representing the feeling of a loss of appetite compared with when-well. Rate by loss of desire for food or the need to force oneself to eat.	
**0** = Normal or increased appetite.	☐
**2** = Slightly reduced appetite.	☐
**4** = No appetite. Food is tasteless.	☐
**6** = Needs persuasion to eat at all.	☐

**6. Concentration difficulties**<br>Representing difficulties in collecting one's thoughts mounting to an incapacitating lack of concentration. Rate according to intensity, frequency, and degree of incapacity produced.	
**0** = No difficulties in concentrating.	☐
**2** = Occasional difficulties in collecting one's thoughts.	☐
**4** = Difficulties in concentrating and sustaining thought which reduced ability to read or hold a conversation.	☐
**6** = Unable to read or converse without great difficulty.	☐

**9. Pessimistic thoughts**<br>Representing thoughts of guilt, inferiority, self-reproach, sinfulness, remorse and ruin.	
**0** = No pessimistic thoughts.	☐
**2** = Fluctuating ideas of failure, self-reproach or self- depreciation.	☐
**4** = Persistent self-accusations, or definite but still rational ideas of guilt or sin. Increasingly pessimistic about the future.	☐
**6** = Delusions of ruin, remorse or irredeemable sin. Self- accusations which are absurd and unshakable.	☐

**10. Suicidal thoughts**<br>Representing the feeling that life is not worth living, that a natural death would be welcome, suicidal thoughts, and preparations for suicide. Suicide attempts should not in themselves influence the rating.	
**0** = Enjoys life or takes it as it comes.	☐
**2** = Weary of life. Only fleeting suicidal thoughts.	☐
**4** = Probably better off dead. Suicidal thoughts are common, and suicide is considered as a possible solution, but without specific plans or intenstion.	☐
**6** = Explicit plans for suicide when there is an opportunity. Active preparations for suicide.	☐

**7. Lassitude**<br>Representing difficulty in getting started or slowness in initiating and performing everyday activities.	
**0** = Hardly any difficulty in getting started. No sluggishness.	☐
**2** = Difficulties in starting activities.	☐
**4** = Difficulties in starting simple routine activities which are carried out with effort.	☐
**6** = Complete lassitude. Unable to do anything without help.	☐

**8. Inability to feel**<br>Representing the subjective experience of reduced interest in the surroundings, or activities that normally give pleasure. The ability to react with adequate emotion to circumstances or people is reduced.	
**0** = Normal interest in the surroundings and in other people.	☐
**2** = Reduced ability to enjoy usual interests.	☐
**4** = Loss of interest in the surroundings. Loss of feelings for friends and acquaintances.	☐
**6** = The experience of being emotionally paralysed, inability to feel anger, grief or pleasure and a complete or even painful failure to feel for close relatives and friends.	☐

4.1.6 Multi Organ Failure Score (MOF-S)

	Normal organ function<br>0 point	Organ dysfunction<br>1 point	Organ failure<br>2 points
Lung	No mechanical ventilation	Mechanical ventilation with PEEP≤10 and FiO_2≤0.4	Mechanical ventilation with PEEP >10 or FiO_2 >0.4
Heart	Normal blood pressure (BP_{syst})	BP_{syst}≥100 mmHg with low dose of vasoactive drugs[a]	Periods with BP_{syst}<100 mmHg and/or high dose of vasoactive drugs[b]
Kidney	Serum creatinine <2 mg/dl (<150 µmol/l)	Serum creatinine≥2 mg/dl (≥150 µmol/l)	Hemodialysis or peritoneal dialysis
Liver	Normal SGOT and bilirubin	SGOT≥25 units/l; bilirubin ≥2 mg/dl (≥ 34 µmol/l)	SGOT≥50 units/l; bilirubin ≥6 mg/dl (≥100 µmol/l)
Blood	Normal counts	Leukocytes ≥30,000; platelets ≤50,000	Leukocytes≥60,000 or ≤2,500
GI tract	Normal	Stress ulcer, Acalculous cholecystitis	Bleeding ulcer; Necrotizing enterocolitis and/or pancreatitis; perforation of gallbladder
CNS	Normal	Diminished responsiveness	Severely disturbed responsiveness; diffuse neuropathy

[a] Dopamine hydrochloride ≤10 µg/kg/min, or nitroglycerin of ≤20 µg/kg/min, or volume loading
[b] Dopamine hydrochloride >10 µg/kg/min, and/or nitroglycerin of >20 µg/kg/min

4.2 Microbiology

The following materials shall be obtained and examined for the molecular biological examination of the possible formation of resistance:

1. Throat Swab
2. Rectal Smear

In the event of the suspicion of an infection the following further tests shall be conducted:

3. quantitative tracheal secretion OR bronchoalveolar lavage
4. blood culture
5. urine culture

In these materials, pathogenic and facultative pathogenic pathogens and/or bacterial flora shall be described in the following manner:

a) phenotypical identification on species level
b) resistance testing with moderate inhibition concentration (MIC)

It shall be endeavoured to obtain a microbiological characterisation for at least 60% of the complicating infections.

4.3 Infection Definitions

4.3.1 Definition Pneumonia

pneumonia

must correspond to one of the following criteria:

Crackles during auscultation

Or

X-ray examination of the thorax shows new or progressive infiltrate, aggregation, cavitation or pleural effusion and one of the following signs

And one of the following signs:

1. productive cough with purulent sputum
2. microorganisms isolated from respiratory tract or blood culture
3. leucocytosis (>12.000)
4. elevation of C reactive protein

4.3.2 Definition of Sepsis

Primary sepsis confirmed by laboratory

must correspond to the following criteria:

Pathogenic pathogens isolated from blood culture, not related to infection at another location*.

One of the following: Pyrexia (> 38 °C), ague or hypotonia (systolic pressure ≤ 90 mmHg) and one of the following:

1. common skin germ, not related to an infection at another location, isolated from two blood cultures taken at different times
2. common skin germ isolated from at least one blood culture from a patient with intravascular antibodies and physician initiates corresponding antimicrobial therapy
3. positive antigen blood test and the pathogen is not related to infection at another location.

Clinical Primary Sepsis

must correspond to the following criteria:

One of the following signs without other recognizable causes:

1. Pyrexia (> 38 °C), hypotonia (systolic pressure ≤ 90 mmHg), oliguria (< 20 ml/h) without otherwise recognizable cause and all of the following signs:
2. No blood culture carried out or no micro-organisms or antigens discovered in the blood.
3. No obvious infection at another location.
4. Physician initiates a therapy for sepsis.

Secondary Sepsis

A germ isolated in a blood culture corresponds with a related nosocomial infection at another location. This shall be classified as secondary sepsis.

4.3.3 Definition of Urinary Tract Infection

Urinary Tract Infection

must correspond with one of the following criteria:

a. Pyrexia (> 38°C)
b. urine culture of > 10^4 colonies/ml urine with no more than two species of micro-organisms
c. urine test strip positive for leucocyte esterase
d. urine test strip positive for leucocyte esterase nitrate

4.4 Immunology

4.4.1 Small Immune Status

a. total T-cells (CP3), T-helper (CD4), T-Cytotoxic(CD8)

b. B-cells (CD19), NK-cells (CD2/CD16)

c. HLA-DR, CD86 on monocytes as Markers of Immune Competence

Mouse experiments have shown that, after a stroke, a fall in the number of the B and T lymphocytes occurs as a result of apoptosis. Therefore, with the parameters a+ b, the relative and absolute number of the important immune cells are registered.

HLA-DR (is expressed most strongly of all human MHC Class II molecules): antigen presenting cells (in the blood monocytes), in addition to co-stimulatory molecules, such as CD86, are essential for initiating T-helper response and thus for the entire, specific, immune reaction. High regulation of HLA-DR/CD86 by immune stimulating cytocines, such as IFNg, IL-12. Low Regulation by immune inhibiting mediators, such as IL-10, TGFß. A quantification is not necessary (Quantibrite Tests).

Proposition: stongly reduced monocytic HLC-DR/CD86 expression (<10.000 and/or 1500 molecules/cell) is a sign of globally limited immune competence (occurance after serious operation, tauma, stress, immune depressive therapy, systemic infection). Persistence of a strongly reduced HLA-DR expression (several days) indicates a high-risk for the development of bacterial/micotic infections, but also of a poor (survival) prognosis for patients with an already existing infection (sepsis) - (extreme condition with < 5000 HLA-DR is referred to as " immune paralysis").

Test: Multi-parameter flow cytometry (Becton-dickson, Heidelberg), CV <20%

Material: one small test-tube EDTA blood

4.4.2 Whole Blood LPS Stimulation (TNFa)

Measurement is made on TNFa secretion after stimulation of heparinised whole blood with bacterial endotoxin (Lipopolysaccharide). Marker for Immune Competence.

This assay comprises the pro-inflammatory capacity of the monocytes and complements the HLA-DR/C86 test in regard to the functional characterisation of these cells. In most cases, both functions run parallel and are influenced in a similar manner, but asynchronic developments are also known.

In the healthy, the LPS-induced TNF secretion is very stable (CV over one year <25%). However, there also exist (up to Factor 5) inter-individual differences in the level of the LPS induced TNFa secretion capacity. BUT: a TNFa secretion below 300 pg/ml in this assay is a reliable marker for monocyte deactivation and shows a very high correlation with HLA-DR expression. Hence, it is a "reinforcement" parameter for propositions regarding immune competence in addition to HLA-DR.

Test: 4-hr whole blood stimulation (Milenia Biotech) with semi-automatic TNF-measurement (Immulite, DPC Biermann, Bad Nauheim), CV < 15 %

Material: 1 ml Heparin blood

4.4.3 Whole Blood ConA Stimulation

Concanavalin A (ConA) stimulated (polyclonal, not antigen-specific) T-lymphocytes for the production of cytocines. During a 24-hr stimulation, the function of the memory/effector T lymphocytes is recorded. The general importance of this text lies in the determination of the so-called T-helpers (Th) 1 and Th 2 cytocines and thus the evaluation of Type 1 (multicellular) and Type 2 (multi-humoral) immune reactivity. Prototypical Th1 cytocine is IFNg, Th2 it is IL-4 and IL-5). In the 24-hr assay, these cytocines are only formed by memory/effector T-cells. Moreover , IL-2 (naive and memory/effector T cells) as well as TNF and IL-10 (for the most part of monocytes after T-cell activation) are also recorded. In immune depressive states (after trauma, shock, Sepsis), a transposition of the Th cytocine-response after ConA Stimulation of Th1 in the direction of Th2 can be found, which is expressed by a reduction of the Th1/Th2-cytocine-ratio (IFNg/IL-4). A lowered IFNg/IL-4 ratio shows a reduced cellular immune reactivity(reduced reactivity against bacterial/viral infections and has prognostic significance.

Test: 24-hour whole blood stimulation with flow-cytometric cytocine measurement (Cytometric based Array [CBA]for IL-2, 4, 5, 10, TNFa, IFNg , Becton-Dickinson,

CV < 20 %

Material: 1 ml Heparin blood

4.4.4 T-Cell Stimulation with MBP/PLP/TT

Test for the determination of the frequency of antigen-specific T-cells. Is used in immunological routine diagnosis(as so-called lymphocyte transformation test= LTT), in order to obtain information regarding quality and quantity of the specific immune response. For this, recall-antigens such as tetanus (TT) are normally used, for which, as a rule, a vaccination status is available).

For this test, mononuclear cells (these are: monocytes and lymphocytes) are isolated from whole blood and cultivated in vitro together with the respective antigens for several days, and subsequently the proliferation of the T-cells and the cytocine production in the supernatant are determined (by means of 3H-thymidine integration). Alternatively, the cytocine production of individual cells and their frequency can be elegantly determined by means of ELISPOT. In the course of the trial, it shall be determined, by testing the MBP/PLP reactivity, whether the frequency of CNS antigen-specific, autoreactive T-cells changes in consequence of a stroke. (Although, according to conventional theory, autoreactive T-cells should be eliminated in the thymus, they are still to be found in small frequency in the periphery.) An increase in their frequency could lead to autoimmune phenomena. In principle, this might be expected after a stroke, since the CNS tissue becomes “visible” for the immune system (all the more in the framework of an inflammation reaction). Hence, it is interesting to discover whether their frequency increases after a stroke or whether this is prevented in the framework of immune suppression after a stroke).

As already mentioned, tetanus toxide is used as a control antigen for this test, as it is a widely used recall-antigen. With the help of recall antigens, the (postulated) immune inhibitory effect of the stroke on the specific immune reactivity can be examined.

4.4.5 Procalcitonin (PCT)

PCT is used as a marker for systemic bacterial/fungal infections. Locally confined infections (e.g. pneumoniae) are marked by normal PCT values. However, PCT is also increased after LPS translocation (from the intestines or the lungs, e.g. after major surgical interventions or traumas). In cardio-surgical patients, an early, temporary increase of PCT, resulting from LPS translocation, has shown itself has a negative prediction parameter for further clinical developments. Nearly all patients who suffered from post-operative infections or ARDS had high PCT values. It is assumed that a perioperative LPS translocation has a causal relationship with the development of an immune depression. The half-life of PCT (24 hrs.) makes this marker interesting for differentiating between LPS translocation and systemic infection by means of multiple measurement.

Test: ELISA, CV < 10 %

Material: 0.5 ml EDTA - or Heparin plasma

4.4.6 DC-PCR

Data obtained by experiments with animals show a massive stroke-induced immune depression. This also affects the thymus. The aim is to prove a thymus atrophia after a stroke, also in humans, by using an indirect method (Deletion Circle PCR). With this method, T-lymphocytes, freshly emigrated from the thymus can be evidenced. In the case of thymus atrophia, we expect a massive fall of the naive thymus emigrants in the peripheral blood. A loss of the thymus function could have both a destructive (sustained immune depression) as well as a protective significance (prevention of autoaggressivity against cerebral tissue).

Test: PCR

Material: one small test-tube EDTA blood

4.5 Biochemistry

4.5.1 AP, Plasma

Abbreviations AP and/or AP/P

Unit U/l

Material: Plasma (Li/Hep.)

General Remarks

Alcalic phosphatases are membrane-linked cellzymes which are localised in great amounts in the skeletal system, in the liver parenchyme and in the duct epithelia of the gall bladder. Therefore, increases in the activity of the entire AP in the serum nearly always result from damage to these organs.

Method

DGKCH, optimised, recommendations 1972 (DEA- Puffer)

Indication:

Suspicion of cholestatic liver disease, bone disease with other basic diseases, such as malignant tumours, kidney diseases, osteomalacia, hyperparathyroidism, deformations of the skeleton.

Heightening

Cholestatic liver diseases, acute and chronic hepatitis, liver cirrhosis, M. Paget, osteomalacia, vitamin D deficiency, phosphate diabetes, metastatic bone tumours, myeloma, hyperthyreosis, pregnancy, acromegaly, malign tumours, hereditory hyperphosphatasemia, Macro AP.

Lowering

Congenital disturbances of the skeletal system, hypothyreosis, critical anaemia, zinc deficiency.

Disturbance Factor

Allopurinol, Carbamazepin, Erythromacin, Ranitadin, Verapamil: increased values; clofibrate, oral contraceptives: lowered values
haemolysis and lipaemia

false lowered values

Analysis Days

CVK & CCM 7x per week

also at weekends; emergency analysis

Ranges of Reference

Men	Women
17 to 99 years<br>up to 180 U/l	17 to 99 years<br>up to 160 U/l

4.5.2 AST (GOT), Plasma

Abbreviations AST and/or AST/P

Unit U/l

Material: Plasma (Li/Hep.)

General Remarks

The ubiquity enzyme is found particularly in cytoplasm and in the mitochondria of myocardial muscle, skeletal muscle and liver cells. The GOT catalyses the transmission of the 2-amino group from the aspartate to 2-Oxoglutarate with the formation of glutamate and oxalacetate. After damage or cell necrosis the enzyme enters into the extracellular space and can be used as a measure for cell damage.

Method

DGKCH, optimised, recommendations 1972

Indication:

Diagnosis, differentiation and assessment of the course of disorders of the liver and the bile ducts, myocardial infarction, damage to the skeletal muscle.

Increasing

Acute and chronic viral hepatitis, malaria, leptospirosis, alcohol hepatitis, toxic liver damage,medicinal products, fatty liver, liver cirrhosis, primary liver carcinoma, liver metastases, cholestases, after epileptic seizures, myocardial infarction, myocarditis, pericarditis, progressive musclar dystrophy, myositis, hypothyreosis

Disturbance Factor

Haemolysis: false increased values
pyridoxalphosphate deficiency: lowered values

Ranges of Reference

Men	Women
17 to 99 years	17 to 99 years
up to 18 U/l	up to 15 U/l

4.5.3 Bilirubin (whole), Plasma

Abbreviations tBil and/or tBil/P

Unit mg/dl

Material: Plasma (Li/Hep.)

General Remarks

Bilirubin is formed to 80-85% from the decomposition of haemoglobin, overmature erythrocites and of 15-20% from the decomposition of other haem-containing proteins; under pathological conditions also in the case of maturation disturbances of the erythrocytes in the bone marrow (innefective erythropoiesis). As a result of poor solubility, bilirubine in the serum is either conjugated to albumin (indirect bilirubin) and/or covalently bound or esterified with glucuronic acid (direct bilirubin).

Method
Photometry (Azobilirubin)

Indication:

Differential diagnosis and control of the course of the icterus

Increasing

Hepatitis, liver cirrhosis, fatty liver, liver tumours, intra-and posthepatic cholestasis, Dubin Johnson syndrome, Rotor syndrome, massive rhabdomyolysis

Disturbance Factor

Sun ray exposure of the sample vessels: false lowered values

Analysis Days

CVK & CCM 7x per week

also at weekends; emergency analysis

Ranges of Reference

14 to 99 years

from 0,1 to 1,2 mg/dl

4.5.4 Large Haemogram, Blood

Abbreviations DIFF. and/or G-BB/B

Material: EDTA-blood

General Remarks

Whilst the peripheral haemogram continues to be an integral component of the diagnosis and control of the development of systemic diseases, its importance for the assessment of inflammatory processes has been relativized by the introduction of CRP determination.

Method

Through-flow cytometry (cytometric/cytochemical/calculation)

Indication:

Diagnosis and control of the development of haematological and malign disorders, of leucocytoses and leucopeniae, of inflammatory, infectious, toxic and allergic changes in the haemogram, monitoring of an immune suppressive or cytostatic therapy.

Analysis Days

CVK & CCM 7x per week

also at weekends; emergency analysis

Parameters contained in this profile

Small haemogram, blood (BB, K-BB/B)
MPV, blood (MPV, MPV/B)
RDW, blood (RDW, RDW/B)
Basophile, blood (Baso%/B)
Eosinophile, blood (Eos%/B)
lymphocytes, blood (Lcyt%/B)
monocytes, blood (Mono%/B)
Neutrophile, blood (Neutr%/B)
Segmented nuclei, blood (Seg%/B)
Rod-shaped nuclei (Rod%/B)
LUC (Large unstained Cells) (LUC%/B)

4.5.5 Cortisol, 24-hr Urine

Abbreviations Cortisol/U/d

Unit nmol/d

Material: Collected urine

General Remarks

90% of the cortisol transported in the blood is conjugated to protein. The free fraction is kept as constant as possible in the control cycle hypothalamus-hypophysis-adrenal gland, whereby the CRH formed in the hypothalamus stimulates the ACTH secretion of the hypophysis and the ACTH, in turn, stimulates the production of cortisol in the adrenal gland. Inversely, cortisol inhibits the secretion of CRH and ACTH. This control cycle is subject to impulses from the central nervous system, which are not precisely characterised. Thereby, there are two biorhythms, one being short-term episodic fluctuations and the other a circadian rhythm with maximum values between 06.00 and 08.00 hrs., falling during the day with minimum values between 00.00 and 06.00 hrs. With the Cushing Sydrome, higher values are found. In mild cases, the base values are often within the normal range in the morning, while the arcadian rhythm is already out of action and, in the evening and night hours, increased values are found. In reverse, an increased cortisol serum is by no means proof of a Cushing Syndrome, since, due to the strong episodic fluctuations, also in healthy people, high peak values can be found.

Indication:

Diagnosis of the hyper and hypocortisolism, analyses while testing the functioning of the adrenal gland (dexamethason, ACTH test, etc.).

Increasing

Cushing Syndrome, Pseudo-Cushing (e.g. alcohol abuse), pregnancy, chronic stress, adiposity

Lowering

M. Addison, adrenogenital Syndrome, sub-function of the Hypophysis

Analysis days

CVK & CCM 5x per week

not at weekends; no emergency analysis

Ranges of Reference

10 to 20 years	20 to 99 years
from 14 to 152 nmol/d	from 55 to 248 nmol/d

4.5.6 CRP, Plasma

Abbreviations CRP and/or CRP/P

Unit mg/dl

Material: Plasma (Li/Hep.)

General Remarks

In the case of acute inflammations (acute tissue lesions, infections), certain plasma proteins increase within 6 - 48 hours (Acute Phase Reaction). As the classical acute phase protein, CRP is a sensitive, albeit unspecific, indicator of such an event. The acute phase proteins are synthesised in the liver and serve as humoral defence. CRP is able to bind a broad spectrum of ligands - both of exogenic as well as endogenic origin - and to activate the complement system. Normal CRP values do not generally contradict an inflammatory event.

Method

Immune Turbidimetry

Indication:

Diagnosis of organ diseases, assessment of the extent of an inflammatory disease, recognition of intercurring infections (e.g. postoperatively developing sepsis), assessment of the success of the therapy (e.g. antibiotic pyelonephritis treatment for children, Gold therapy for rheumatoid arthritis), prognostic proposition (e.g. with infectious diseases, malignant tumours).

Increasing

1-10 mg/dl indicate mild to moderate inflammatory processes, or such with small extent, e.g. local bacterial infektions, uncomplicated cystitis, bronchitis, traumas, postoperative, accident, myocardia infarct, tuberculosis, sarcoidosis. 10 mg/dl with acute disease events speak for high and/oder extensive inflammatory activity (Sepsis, larger traumas, bacterial infections, metastasing tumours, active rheumatoid arthritis, seronegative spondylarthritis. Immune Vasculitis, Polymyalgia rheumatica, Morbus Crohn, deep vein thrombosis, etc.).

Analysis days

CVK & CCM 7x per Week

also at weekends; emergency analysis

Ranges of Reference

1 month to 99 years	up to 0,82 mg/dl

4.5.7 Glucose, Plasma

Abbreviations Gluc and/or Gluc/P

Unit mg/dl

Material: Plasma (Li/Hep.)

General Remarks

The blood glucose concentration is largely regulated by the hormones insulin and glucagon. Insulin develops its effect, among other things, by increasing the glycogen synthesis and inhibiting the glycogenolysis and the glycogenesis; glucagon has the contrary effect. Above a glucose concentration of approximately 180 mg /dl in the blood, the renal threshold is exceeded and glucose appears in the urine.

Method

Photometry (Hexokinases)

Indication:

Recognition of a diabetic disturbance of the metabolism, therapy and control of the development of mellitus, proof of hypoglycaemia

Increasing

Diabetes mellitus, pancreas disorders, endocrinological disorders (M. Cushing, acromegalia, phaeochromocytoma, hyperthyreosis), haemochromatosis, liver disorders

Lowering

Overdosage of antidiabetics, island-cell tumours, extrapancreatic tumours, malnutrition, heavy physical labour, malabsoprtion, chronic alcoholism, liver disorders.

Disturbance Factor

Antidiabetics: lowered values
long sample storage time: false lowered values

Analysis Days

CVK & CCM 7x per week

also at weekends; emergency analysis

Ranges of Reference

7 to 65 years

from 60 to 100 mg/dl

4.5.8 Potassium, Plasma

Abbreviations P and/or P/P

Unit mmol/l

Material: Plasma (Li/Hep.)

General Remarks

Potassium is the main cation in the intracellular space (ICS), whereas in the extracellular space (ECS) only small amounts are found. 98% of the body potassium is found in the ICS and only 2% in the ECS. This concentration gradient (internal balance) is maintained by the Na-K-ATPase of the cell membrane and is influenced by the acid base status, by insulin, catecholamine and aldosterone. The external potassium balance is largely regulated by renal elimination and is subject to modulating influences, such as the potassium amount added through food, the sodium and chloride content in the distal tubulus, the pH value and the activity of the mineral corticoids.

Method

Indirect ISE

Indication:

Disturbances in the electrolyte-acid bases and water content, cardiac dysrhythmia, renal insufficiency

Sampling Conditions

haemolysis-free

Increasing

renal insufficiency, acute acidosis, cell collapse, increased influx, M. Addison, diabetes mellitus

Lowering

Alcalosis, vomiting, diarrhoea, severe combustions, hyperaldosteronism, M. Cushing, renal tubular acidosis, inadequate influx

Disturbance Factor

Potassium-saving diuretics, pentamidine, ACE inhibitors, beta-blocker, digitalis, heparin, cyclosporine A: increased values
haemolysis, leucocytosis, thrombocytosis false lowered values

Analysis Days

CVK & CCM 7x per week

also at weekends; emergency analysis

Ranges of Reference

15 to 65 years	65 to 99 years
from 3.7 bis 5.4 mmol/l	from 3.6 to 4.8 mmol/l

4.5.9 Creatinine, Plasma

Abbreviations Crea and/or Crea/P

Unit mg/dl

Material: Plasma (Li/Hep.)

General Remarks

The creatinine formed in the liver, pancreas and kidney is converted in the muscle tissue into creatine phosphate. Creatine serves as an energy storer and carrier in the muscle tissue. In the decomposition of creatine and creatine phosphate creatinine is formed non-enzymatically, which is completely eliminated by glomular filtration. The amount of creatinine formed and its concentration in the plasma are individually specific and dependent upon the muscle mass and, hence, indirectly upon constitution, sex and age.

Method

Enzymatic stain test (Creatininase) Cratinine Plus (Roche Diagnostics)

Indication:

Screening examination, diagnosis and control of the development of renal disorders, in pathological urine findings, extra-renal disorders with fluid loss.

Increasing

Renal insufficiency (after a reduction of the GFR by more than 50%) muscle traumata, rhabdomyolysis, muscle dystrophia, acromegalia, combustions

Lowering

Gravidity, juvenile diabetes mellitus, myopathiae, reduction of the muscle mass

Disturbance Factor

Bilirubin anaemia: false lowered values

Analysis Days

CVK & CCM 7x per week

also at weekends; emergency analysis

Ranges of Reference

Men	Women
18 to 99 years	18 to 99 years
from 0.67 to 1.17 mg/dl	from 0.51 to 0.95 mg/dl

4.5.10 Sodium, Plasma

Abbreviations Na and/or Na/P

Unit mmol/l

Material: Plasma (Li/Hep.)

Method

Indirect ISE (Hitachi)

Sampling Conditions

Li-Heparinate

Disturbance Factor

Lipidaemia, Hypo and/or Hyperproteinaemia! (Direct ISE to be used!)

Analysis Days

CVK & CCM 7x per week

also at weekends; emergency analysis

Ranges of Reference

18 to 65 years	65 to 99 years
from 133 to 145 nmol/d	from 132 to 146 nmol/d

4.5.11 Noradrenaline, 24-hr. Urine

Abbreviations Noradr and/or Noradr/U/d

Unit nmol/d

Material: Collected urine

General Remarks

Catecholamine dopamine, noradrenaline and adrenaline are formed in the sympathoadrenal system (brain, adrenal gland marrow, extra-adrenal chromaffine tissue, sympathetic nerve endings) in a controlled synthesis chain - starting from amino acid L Tyrosine via the intermediate product L-dopa. The adrenal medulla secretes for the large part adrenaline, the sympathetic nerve endings mainly noradrenaline; dopamine is the transmitter substance in the CNS. On suspicion of phaeochromocytoma, initial determination is reommended via the analysis of adrenaline and noradrenaline (sensitivity 67-100%) in the 24-hr. urine. The determination of vanillin mandelic acid in the collected urine, in contrast, possesses lower sensitivity (42-100%).

Method

High performance liquid chromatography

Indication:

many other phaeochromocytoma, dissorders associated with phaeochromocytoma (neurofibromatosis, Men Type 2 and Type 3), arterial hypertonia

Sampling Conditions

Special vessel (contains 20ml 10 percentile hydrochloric acid) request from the central laboratory

Increasing

Phaeochromocytoma, arterial hypertonia, renal artery stenosis

Lowering

Shy-Drager-Syndrome, Lesh-Nyhan-Syndrome, Riley-Day-Syndrome (familiar Dysautonomia)

Disturbance Factor

Coffein, Adrenaline, Alcohol, L-Dopa, Nikotine, Nitroglycerine, Reserpine, Theophylline: increased values
Clonidine, Prazosin: lowered values

Analysis Days

CVK & CCM 2x per week

not at weekends; no emergency analysis

Ranges of Reference

0 to 99 years

from 136 to 620 nmol/d

4.5.12 Urine Strip Test

Abbreviations Stix: and/or UStat/TS

Material: Spontaneous urine

Method Reflectrometry (test strip)

Indication: Disorder of the kidney and evacuating urinary tracts, screening examination

Sampling Conditionsfresh mid-flow urine

Analysis Days

CVK & CCM 7x per week

also at weekends; emergency analysis

Parameters contained in this profile

Erythrocytes, Urine (Ery/U-F)
Bacterian evidence (Nitritate), Urine (Bact/U-TS)
Glucose, Urine (Gluc, Gluc/U)
Leucocytes, Urine (Leuc/U-F)
Protein, Urine (Prot, Prot/U)
Bilirubin, Urine (Bili/U-TS)
pH, Urine (pH/U-TS)
Density, urine (Density/U-TS)
Urobilinogen, Urine (Ubg/U-TS)

4.6 The Manufacturing of the Investigational Medicinal product

The manufacturing shall take place under aseptic conditions in Room 1.0113 on the workbench on the right.

4.6.1 Moxifloxacin (Verum)

Connect the Moxifloxacin infusion flask to the infusion system and the infusion tube to a 250 ml EVA infusion bag. Convey the solution by means of gravity. Seal the finished bag with a red cone, label it and pack it inside a black, lightproof sack. The sack shall also be labelled.

Materials:

1 Moxifloxacin 400 mg/250ml	R. 1.0117
1 EVA infusion bag 250 ml	R. 1.0117
1 Infusion system	R. 1.0117
1 cone	R. 1.0113
1 lightproof sack, black	R. 1.0117
2 labels	File folder "Moxifloxacin/Pantheris"
1 label "Store at Room Temperature"	File folder "Moxifloxacin/Pantheris"

4.6.2 Riboflavin (Placebo)

Manufacturing a Loading Solution:

Manufacturing regulation with 1 vial Vitamin B2 injectopas 20 mg/ml = 20 mg, Riboflavin - 5′-phosphate, monosodium salt 2 H_2O = 14.6 mg Riboflavin:

From one vial, draw Vitamin B2 Injektopas 1.00 ml and top up with NaCl 0.9% to 20 ml in a syringe.

The Manufacturing of the infusion solution

From this loading solution 0.68ml (o.5mg) inject 250 ml NaCl 0.9 % into an infusion flask. After brief shaking, connect the flask with the infusion attachments and the infusion tube with a 250ml EVA infusion bag. Convey the solution by means of gravity. Seal the finished bag with a red cone, label it and pack it inside a black, lightproof sack. The sack shall also be labelled.

Materials:

1 vial Vitamin B2 Injectopas 20 mg/ml - manufactured by Pascoe	R. 10117
1 NaCl 0.9 % 250ml	R. 1.0117
1 syringe 1 ml	R. 1.0113
1 syringe 20 ml	R. 1.0113
1 EVA infusion bag 250 ml	R. 1.0117
1 Infusion system	R. 1.0117
1 cone	R. 1.0113
1 lightproof sack, black	R. 1.0117
2 labels	File folder "Moxifloxacin/Pantheris"
1 label "Store at Room Temperature"	File folder "Moxifloxacin/Pantheris"

4.7 AVELOX® – product characteristics

SUMMARY OF PRODUCT CHARACTERISTICS

1. NAME OF THE MEDICINAL PRODUCT

Avelox 400 mg/250 ml solution for infusion

2. QUALITATIVE AND QUANTITATIVE COMPOSITION

Moxifloxacin 1.6 mg/ml (400 mg/250 ml) (as moxifloxacin hydrochloride).
Excipient: The solution for infusion (250 ml) contains 34 mmol sodium (see section 4.4).
For a full list of excipients, see section 6.1.

3. PHARMACEUTICAL FORM

Solution for infusion
Clear, yellow solution

4. CLINICAL PARTICULARS

4.1 Therapeutic indications

Avelox 400 mg solution for infusion is indicated for the treatment of:
- Community acquired pneumonia
- Complicated skin and skin structure infections (see section 4.4)

caused by bacteria susceptible to moxifloxacin in patients requiring initial parenteral therapy.

Consideration should be given to official guidance on the appropriate use of antibacterial agents.

4.2 Posology and method of administration

Dosage (adults)
400 mg moxifloxacin, infused once daily.

For community acquired pneumonia therapy may be initial intravenous administration, followed by oral tablet administration, when clinically indicated.

For treatment of complicated skin and skin structure infections requiring initial intravenous therapy followed by oral administration of 400 mg moxifloxacin tablets.

Renal/hepatic impairment
No adjustment of dosage is required in patients with mild to severely impaired renal function or in patients on chronic dialysis i.e. haemodialysis and continuous ambulatory peritoneal dialysis (see section 5.2 for more details).
There is insufficient data in patients with impaired liver function (see section 4.3).

Other special populations
No adjustment of dosage is required in the elderly and in patients with low bodyweight.

Children and adolescents
Moxifloxacin is contraindicated in children and growing adolescents. Efficacy and safety of moxifloxacin in children and adolescents have not been established (see section 4.3).

Method of administration
For intravenous use; **constant infusion over 60 minutes** (see also section 4.4).

If medically indicated the solution for infusion can be administered via a T-tube, together with compatible infusion solutions (see section 6.6).

Duration of administration
The recommended total treatment duration for sequential administration (intravenous followed by oral) is dependent on the indication, the type and severity of the disease and the clinical response:
-Community acquired pneumonia 7 - 14 days
In clinical trials in hospitalised patients with community acquired pneumonia most patients were switched to oral therapy within 4 days.

-Complicated skin and skin structure infections 7 - 21 days
In clinical trials in patients with complicated skin and skin structure infections the mean duration of intravenous therapy was approximately 6 days with an overall mean treatment duration of approximately 13 days.

The recommended dose (400 mg once daily) and duration of therapy for the indication being treated should not be exceeded.

4.3 Contraindications

- Hypersensitivity to moxifloxacin, other quinolones or to any of the excipients.
- Pregnancy and lactation (see section 4.6).
- Children and growing adolescents.
- Patients with a history of tendon disease/disorder related to quinolone treatment.

Both in preclinical investigations and in humans, changes in cardiac electrophysiology have been observed following exposure to moxifloxacin, in the form of QT prolongation. For reasons of drug safety, moxifloxacin is therefore contraindicated in patients with:
- Congenital or documented acquired QT prolongation
- Electrolyte disturbances, particularly in uncorrected hypokalaemia
- Clinically relevant bradycardia
- Clinically relevant heart failure with reduced left-ventricular ejection fraction
- Previous history of symptomatic arrhythmias

Moxifloxacin should not be used concurrently with other drugs that prolong the QT interval (see also section 4.5).

Due to limited clinical data, moxifloxacin is also contraindicated in patients with impaired liver function (Child Pugh C) and in patients with transaminases increase > 5fold ULN.

4.4 Special warnings and precautions for use

- Moxifloxacin solution for infusion is for intravenous administration only. Intra-arterial administration should be avoided since preclinical studies demonstrated peri-arterial tissue inflammation following infusion by this route.
- Moxifloxacin has been shown to prolong the QTc interval on the electrocardiogram in some patients. In the analysis of ECGs obtained in the clinical trial program, QTc prolongation associated with the 400 mg dose of moxifloxacin in steady state reached on day 3 (see section 5.2) was in the same range after intravenous as compared to oral administration (60 minutes infusion: 7 ± 30 msec, 1.6% compared to baseline; tablet: 6 ± 26 msec, 1.4% compared to baseline).

  Medication that can reduce potassium levels should be used with caution in patients receiving moxifloxacin.

  Moxifloxacin should be used with caution in patients with ongoing proarrhythmic conditions, such as acute myocardial ischaemia or QT prolongation as this may lead to an increased risk for ventricular arrhythmias (incl. torsade de pointes) and cardiac arrest (see also section 4.3). The magnitude of QT prolongation may increase with the infusion rate and with increasing plasma concentrations of the drug. Therefore, the recommended duration of infusion (60 minutes) should not be shortened and the recommended dose should not be exceeded.

  Intravenous therapy should be initiated with caution and patients should be carefully monitored.

  If signs of cardiac arrhythmia occur during treatment with moxifloxacin, treatment should be stopped and an ECG should be performed.
- Hypersensitivity and allergic reactions have been reported for fluoroquinolones including moxifloxacin after first administration. Anaphylactic reactions can progress to a life threatening shock, even after the first administration. In these cases moxifloxacin should be discontinued and suitable treatment (e.g. treatment for shock) initiated.
- Quinolones are known to trigger seizures. Use should be with caution in patients with CNS disorders which may predispose to seizures or lower the seizure threshold.
- Pseudomembranous colitis has been reported in association with the use of broad spectrum antibiotics including moxifloxacin; therefore it is important to consider this diagnosis in patients who develop serious diarrhoea during or after the use of moxifloxacin. In this situation adequate therapeutic measures should be initiated immediately. Drugs inhibiting peristalsis are contraindicated in this situation.
- Elderly patients with renal disorders should use moxifloxacin with caution if they are unable to maintain adequate fluid intake, because dehydration may increase the risk of renal failure.
- Tendon inflammation and rupture may occur with quinolone therapy including moxifloxacin, particularly in elderly patients and in those treated concurrently with corticosteroids. At the first sign of pain or inflammation, patients should discontinue treatment with moxifloxacin and rest the affected limb(s).
- Liver function tests/investigations should be performed in cases where indications of liver dysfunction occur.
- Patients with a family history of, or actual glucose-6-phosphate dehydrogenase deficiency are prone to haemolytic reactions when treated with quinolones. Therefore, moxifloxacin should be used with caution in these patients.
- If vision becomes impaired or any effects on the eyes are experienced, an eye specialist should be consulted immediately.

- Quinolones have been shown to cause photosensitivity reactions in patients. However, studies have shown that moxifloxacin has a lower risk to induce photosensitivity. Nevertheless patients should be advised to avoid exposure to either UV irradiation or extensive and/or strong sunlight during treatment with moxifloxacin.
- Experience of the use of sequential intravenous/oral moxifloxacin in the treatment of severe community-acquired pneumonia (defined as Pneumonia Severity Index > III) is currently limited to approximately 25% of patients treated with moxifloxacin in clinical studies.
- Clinical efficacy of moxifloxacin in the treatment of severe burn infections, fasciitis, major abscesses and diabetic foot infections with osteomyelitis has not been established.
- This medicinal product contains 787 mg (approximately 34 mmol) sodium per dose. To be taken into consideration by patients on a controlled sodium diet.

4.5 Interaction with other medicinal products and other forms of interaction

Interactions with medicinal products
An additive effect on QT interval prolongation between moxifloxacin and the following drugs cannot be excluded: antiarrhythmics class IA (e.g. quinidine, hydroquinidine, disopyramide) or antiarrhythmics class III (e.g. amiodarone, sotalol, dofetilide, ibutilide), neuroleptics (e.g. phenothiazines, pimozide, sertindole, haloperidol, sultopride), tricyclic antidepressive agents, certain antimicrobials (sparfloxacin, erythromycin IV, pentamidine, antimalarials particularly halofantrine), certain antihistaminics (terfenadine, astemizole, mizolastine), others (cisapride, vincamine IV, bepridil, diphemanil). This effect might lead to an increased risk of ventricular arrhythmias, notably torsade de pointes. Therefore moxifloxacin is contraindicated in patients treated with these drugs (see also section 4.3).

After repeated dosing in healthy volunteers moxifloxacin increased C_{max} of digoxin approximately 30% without affecting AUC or trough levels. No precaution is required for use with digoxin.

In studies conducted in diabetic volunteers, concomitant administration of oral moxifloxacin with glibenclamide resulted in a decrease of approximately 21% in the peak plasma concentrations of glibenclamide. The combination of glibenclamide and moxifloxacin could theoretically result in a mild and transient hyperglycaemia. However, the observed pharmacokinetic changes for glibenclamide did not result in changes of the pharmacodynamic parameters (blood glucose, insulin). Therefore no clinically relevant interaction was observed between moxifloxacin and glibenclamide.

Changes in INR
A large number of cases showing an increase in oral anticoagulant activity have been reported in patients receiving antibiotics, especially fluoroquinolones, macrolides, tetracyclines, cotrimoxazole and some cephalosporins. The infectious and inflammatory conditions, age and general status of the patient appear to be risk factors. Under these circumstances, it is difficult to evaluate whether the infection or the antibiotic therapy cause the INR (international normalised ratio) disorder. A precautionary measure would be to more frequently monitor the INR. If necessary, the oral anticoagulant dosage should be adjusted as appropriate. Even if during an interaction study performed in healthy volunteers between moxifloxacin and warfarin, negative results have been observed, the precautionary measures as above stated should apply to warfarin as for other anticoagulants.

Clinical studies have shown that there are no interactions following concomitant administration of moxifloxacin with: ranitidine, probenecid, oral contraceptives, calcium supplements, morphine administered parenterally, theophylline or itraconazole.

In vitro studies with human cytochrome P450 enzymes support this data. Considering these results a metabolic interaction via cytochrome P450 enzymes is unlikely.

Interaction with food
Moxifloxacin has no clinically relevant interaction with food including dairy products.

4.6 Pregnancy and lactation

Pregnancy
The use of moxifloxacin during pregnancy is contraindicated. The safety of moxifloxacin in human pregnancy has not been evaluated. Reversible joint injuries are described in children receiving some quinolones, however this effect has not been reported as occurring on exposed foetuses. Animal studies have shown reproductive toxicity (see section 5.3). The potential risk for humans is unknown.

Lactation
The use of moxifloxacin during breast feeding is contraindicated. As with other quinolones, moxifloxacin has been shown to cause lesions in the cartilage of the weight bearing joints of immature animals. Preclinical data indicate that moxifloxacin passes into milk.

4.7 Effects on ability to drive and use machines

No studies on the effects of moxifloxacin on the ability to drive and use machines have been performed. However, fluoroquinolones including moxifloxacin may result in an impairment of the patient's ability to drive or operate machinery due to CNS reactions (e.g. dizziness, see section 4.8). Patients should be advised to see how they react to moxifloxacin before driving or operating machinery.

4.8 Undesirable effects

Adverse reactions based on all clinical trials with moxifloxacin 400 mg (oral and sequential therapy) sorted by frequencies are listed below:

Apart from nausea and diarrhoea all adverse reactions were observed at frequencies below 3%.

**Common**<br>≥1% to <10%	**Uncommon**<br>≥0.1% to <1%	**Rare**<br>≥0.01% to <0.1%	**Very Rare**<br><0.01%
**Infections and Infestations**			
Superinfections due to resistant bacteria or fungi e.g. oral and vaginal candidiasis			
**Blood and Lymphatic System Disorders**			

**Common**<br>≥1% to <10%	**Uncommon**<br>≥0.1% to <1%	**Rare**<br>≥0.01% to <0.1%	**Very Rare**<br><0.01%
	Anemia<br>Leucopenia(s)<br>Neutropenia<br>Thrombocytopenia<br>Thrombocythemia<br>Blood eosinophilia	Prothrombin time prolonged / INR increased	Prothrombin level increased / INR decreased
**Immune System Disorders**			
	Allergic reaction	Anaphylaxis incl. very rarely life threatening shock<br>Allergic edema / angioedema (incl. laryngeal edema, potentially life threatening)	
**Metabolic and Nutritional Disorders**			
	Hyperlipidemia	Hyperglycemia<br>Hyperuricemia	
**Psychiatric Disorders**			
	Anxiety reactions<br>Psychomotor hyperactivity	Emotional lability<br>Depression (in very rare cases potentially culminating in self-endangering behaviour)<br>Hallucination	Depersonalization<br>Psychotic reaction (potentially culminating in self-endangering behaviour)
**Nervous System Disorders**			
Headache<br>Dizziness	Par- / Dysesthesia<br>Taste disorder (incl. ageusia in very rare cases)<br>Confusion and disorientation<br>Sleep disorder (predominantly insomnia)<br>Tremor<br>Vertigo<br>Somnolence	Hypoesthesia<br>Smell disorders (incl. anosmia)<br>Abnormal dreams<br>Disturbed coordination (incl. gait disturbances, esp. due to dizziness or vertigo)<br>Seizures incl. grand mal convulsions<br>Disturbed attention<br>Speech disorders<br>Amnesia	Hyperesthesia
**Eye Disorders**			
	Visual disturbances incl. diplopia and blurred vision (especially in the course of CNS reactions)		
**Ear and Labyrinth Disorders**			
		Tinnitus	
**Cardiovascular System Disorders**			
QT prolongation in patients with hypokalaemia	QT prolongation<br>Palpitations<br>Tachycardia<br>Atrial fibrillation<br>Angina pectoris	Ventricular tachyarrhythmias<br>Syncope<br>Hypertension<br>Hypotension<br>Vasodilatation	Unspecific arrhythmia<br>Torsade de Pointes (see section 4.4)<br>Cardiac arrest (see section 4.4)
**Respiratory Disorders**			

Common<br>≥1% to <10%	Uncommon<br>≥0.1% to <1%	Rare<br>≥0.01% to <0.1%	Very Rare<br><0.01%
	Dyspnea (including asthmatic conditions)		
**Gastrointestinal Disorders**			
Nausea<br>Vomiting<br>Gastrointestinal and abdominal pains<br>Diarrhoea	Anorexia<br>Constipation<br>Dyspepsia<br>Flatulence<br>Gastritis<br>Increased amylase	Dysphagia<br>Pseudomembranous colitis (in very rare cases associated with life threatening complications)	
**Hepatobiliary Disorders**			
Increase in transaminases	Hepatic impairment (incl. LDH increase)<br>Increased bilirubin<br>Increased gamma-glutamyl-transferase<br>Increase in blood alkaline phosphatase	Jaundice<br>Hepatitis (predominantly cholestatic)	
**Skin and Subcutaneous Tissue Disorders**			
	Pruritus<br>Rash<br>Urticaria<br>Dry skin		Stevens-Johnson-Syndrome
**Musculoskeletal and Connective Tissue Issue Disorders**			
	Arthralgia<br>Myalgia	Tendonitis<br>Muscle cramp<br>Muscle twitching	Tendon rupture<br>Arthritis<br>Muscle rigidity
**Renal and Urinary Disorders**			
	Dehydration	Renal impairment (incl. increase in BUN and creatinine)<br>Renal failure (see section 4.4)	
**General Disorders and Administration Site Conditions**			
Injection and infusion site reactions	Asthenia<br>Painful conditions (incl. pain in back, chest, pelvic and extremities)<br>Sweating<br>Infusion site (thrombo-) phlebitis	Peripheral edema	

The following undesirable effects have a higher frequency category in the subgroup of IV treated patients with or without subsequent oral therapy:

Common: Increased gamma-glutamyl-transferase

Uncommon: Ventricular tachyarrythmias, hypotension, vasodilatation, pseudomembranous colitis (in very rare cases associated with life threatening complications), seizures incl. grand mal convulsions, hallucination, renal impairment (incl. increase in BUN and creatinine), renal failure (see section 4.4)

There have been very rare cases of the following side effects reported following treatment with other fluoroquinolones, which might possibly also occur during treatment with moxifloxacin: transient loss of vision, hypernatraemia, hypercalcaemia, haemolysis, photosensitivity reactions.

4.9 Overdose

No specific countermeasures after accidental overdose are recommended. General symptomatic therapy should be initiated. Concomitant administration of charcoal with a dose of 400 mg oral or intravenous moxifloxacin will reduce systemic availability of the drug by more than 80% or 20% respectively. The use of charcoal early during absorption may be useful to prevent excessive increase in the systemic exposure to moxifloxacin in cases of oral overdose.

5. PHARMACOLOGICAL PROPERTIES

5.1 Pharmacodynamic properties

Pharmacotherapeutic group: Quinolone antibacterials, fluoroquinolones, ATC code: J01 MA 14

Mechanism of action
Moxifloxacin has *in vitro* activity against a wide range of Gram-positive and Gram-negative pathogens.
The bactericidal action of moxifloxacin results from the inhibition of both type II topoisomerases (DNA gyrase and topoisomerase IV) required for bacterial DNA replication, transcription and repair. It appears that the C8-methoxy moiety contributes to enhanced activity and lower selection of resistant mutants of Gram-positive bacteria compared to the C8-H moiety. The presence of the bulky bicycloamine substituent at the C-7 position prevents active efflux, associated with the *nor*A or *pmr*A genes seen in certain Gram-positive bacteria.
Pharmacodynamic investigations have demonstrated that moxifloxacin exhibits a concentration dependent killing rate. Minimum bactericidal concentrations (MBC) were found to be in the range of the minimum inhibitory concentrations (MIC).

Interference with culture test
Moxifloxacin therapy may give false negative culture results for *Mycobacterium* spp. by suppression of mycobacterial growth.

Effect on the intestinal flora in humans
The following changes in the intestinal flora were seen in volunteers following oral administration of moxifloxacin: *Escherichia coli*, *Bacillus* spp., *Enterococcus* spp., and *Klebsiella* spp. were reduced, as were the anaerobes *Bacteroides vulgatus*, *Bifidobacterium* spp., *Eubacterium* spp., and *Peptostreptococcus* spp.. For *Bacteroides fragilis* there was an increase. These changes returned to normal within two weeks.

Mechanism of resistance
Resistance mechanisms that inactivate penicillins, cephalosporins, aminoglycosides, macrolides and tetracyclines do not interfere with the antibacterial activity of moxifloxacin. Other resistance mechanisms such as permeation barriers (common in *Pseudomonas aeruginosa*) and efflux mechanisms may also effect susceptibility to moxifloxacin.
In vitro resistance to moxifloxacin is acquired through a stepwise process by target site mutations in both type II topoisomerases, DNA gyrase and topoisomerase IV. Moxifloxacin is a poor substrate for active efflux mechanisms in Gram-positive organisms.
Cross-resistance is observed with other fluoroquinolones. However, as moxifloxacin inhibits both topoisomerase II and IV with similar activity in some Gram-positive bacteria, such bacteria may be resistant to other quinolones, but susceptible to moxifloxacin.

In vitro Susceptibility Data

EUCAST clinical MIC breakpoints for moxifloxacin (31.01.2006):

Organism	Susceptible	Resistant
Staphylococcus spp.	≤ 0.5 mg/l	> 1 mg/l
S. pneumoniae	≤ 0.5 mg/l	> 0.5 mg/l
Streptococcus Groups A, B, C, G	≤ 0.5 mg/l	> 1 mg/l
H. influenzae and *M. catarrhalis*	≤ 0.5 mg/l	> 0.5 mg/l
Enterobacteriaceae	≤ 0.5 mg/l	> 1 mg/l
Non-species related breakpoints*	≤ 0.5 mg/l	> 1 mg/l
* Non-species related breakpoints have been determined mainly on the basis of pharmacokinetic/pharmacodynamic data and are independent of MIC distributions of specific species. They are for use only for species that have not been given a species-specific breakpoint and are not for use with species where interpretative criteria remain to be determined (Gram-negative anaerobes).		

Clinical and Laboratory Standards Institute™ (CLSI), formerly NCCLS breakpoints are presented in the below table for MIC testing (mg/l) or disc diffusion testing (zone diameter [mm]) using a 5-μg moxifloxacin disc.

Clinical and Laboratory Standards Institute™ (CLSI) MIC and disc diffusion breakpoints for aerobes (M100-S16, 2006) and MIC breakpoints for anaerobes (M11-A7, 2007):

Organism	Susceptible	Intermediate	Resistant
S. pneumoniae	≤ 1 mg/l<br>≥ 18 mm	2 mg/l<br>15-17 mm	≥ 4 mg/l<br>≤ 14 mm
Haemophilus spp.	≤ 1 mg/l<br>≥ 18 mm	-<br>-	-<br>-
Staphylococcus spp.	≤ 0.5 mg/l<br>≥ 24 mm	1 mg/l<br>21-23 mm	≥ 2 mg/l<br>≤ 20 mm
Anaerobes	≤ 2 mg/l	4 mg/l	≥ 8 mg/l

The prevalence of acquired resistance may vary geographically and with time for selected species and local information of resistance is desirable, particularly when treating severe infections. As necessary, expert advice should be sought where the local prevalence of resistance is such that utility of the agent in at least some types of infections is questionable.

**Commonly susceptible species**
Aerobic Gram-positive micro-organisms<br>*Staphylococcus aureus** (methicillin-susceptible)<br>*Streptococcus agalactiae* (Group B)<br>*Streptococcus milleri* group* (*S. anginosus, S. constellatus and S. intermedius*)<br>*Streptococcus pneumoniae**<br>*Streptococcus pyogenes** (Group A)
Aerobic Gram-negative micro-organisms<br>*Haemophilus influenzae**<br>*Klebsiella pneumoniae**[#]<br>*Moraxella (Branhamella) catarrhalis*<br>*Proteus mirabilis**
Anaerobic micro-organisms<br>*Bacteroides fragilis*<br>*Prevotella* spp.*
"Other" micro-organisms<br>*Chlamydophila (Chlamydia) pneumoniae**<br>*Coxiella burnettii*<br>*Legionella pneumophila*<br>*Mycoplasma pneumoniae**
**Species for which acquired resistance may be a problem**
Aerobic Gram-positive micro-organisms<br>*Staphylococcus aureus* (methicillin-resistant)[+]
Aerobic Gram-negative micro-organisms<br>*Enterobacter cloacae*<br>*Enterococcus faecalis**<br>*Escherichia coli**<br>*Klebsiella oxytoca*
**Inherently resistant organisms**
Aerobic Gram-negative micro-organisms<br>*Enterococcus faecalis* (gentamicin-resistant)<br>*Pseudomonas aeruginosa*
*Activity has been satisfactorily demonstrated in clinical studies.<br>[#]ESBL-producing strains are commonly resistant to fluoroquinolones<br>[+]Resistance rate > 50% in one or more countries

Comparison of Pharmacokinetic/Pharmacodynamic (PK/PD) surrogate parameters
Certain pharmacokinetic/pharmacodynamic parameters appear to predict clinical efficacy of antibiotics. For quinolones and in patients requiring hospitalisation, an AUC/MIC_{90} ratio of greater than 125 and a C_{max}/MIC_{90} ratio of 8 - 10 are predictive for clinical cure. In patients suffering from community acquired pneumococcal infections, these surrogate parameters are generally smaller, i.e. AUC/MIC_{90} greater than 30 - 40 is predictive of clinical efficacy. The following table provides the respective PK/PD surrogates for intravenous and oral administration of 400 mg moxifloxacin calculated from single dose data:

**Mode of administration**	**intravenous**		**oral**	
**Parameter** (median)	AUC/MIC_{90} [h]	C_{max}/MIC_{90} [a]	AUC/MIC_{90} [h]	C_{max}/MIC_{90}
MIC_{90} 0.125 mg/l	313	32.5	279	23.6
MIC_{90} 0.25 mg/l	156	16.2	140	11.8

[a] 1hour infusion

For example, in case of a hospital infection caused by *Streptococcus pneumoniae* with a minimal inhibitory concentration of 0.125 mg/l, and treated with 400 mg moxifloxacin, AUC/MIC and C_{max}/MIC would be at least twice above the required threshold values predictive of clinical efficacy, independent of the route of administration.

5.2 Pharmacokinetic properties

Absorption and Bioavailability

After a single 400 mg intravenous 1 hour infusion peak plasma concentrations of approximately 4.1 mg/l were observed at the end of the infusion corresponding to a mean increase of approximately 26% relative to those seen after oral administration (3.1 mg/l). The AUC value of approximately 39 mg·h/l after i.v. administration is only slightly higher than that observed after oral administration (35 mg·h/l) in accordance with the absolute bioavailability of approximately 91%. In healthy subjects mean peak and trough plasma concentrations at steady-state following 400 mg 1 hour infusion once daily were 4.1 - 5.9 and 0.43 - 0.84 mg/l, respectively vs. 3.2 and 0.6 mg/l, respectively following oral administration. In patients mean peak plasma concentrations of 4.4 mg/l were observed at steady-state.

In Phase I studies, higher plasma concentrations were observed in volunteers with low body weight (such as women) and in elderly volunteers. This was not confirmed in Phase III clinical trials. In patients, there is no need for age or gender related dose adjustment on intravenous moxifloxacin.

Pharmacokinetics are linear in the range of 50 - 1200 mg single oral dose, up to 600 mg single intravenous dose and up to 600 mg once daily dosing over 10 days.

There is a linear relationship between moxifloxacin C_{max} and QTc prolongation characterised by a rather flat curve. Most likely there is a delay in time between C_{max} and the maximum QTc prolongation.

Distribution

Moxifloxacin is distributed to extravascular spaces rapidly. The steady-state volume of distribution (Vss) is approximately 2 l/kg. *In vitro* and *ex vivo* experiments showed a protein binding of approximately 40 - 42% independent of the concentration of the drug. Moxifloxacin is mainly bound to serum albumin.

The following peak concentrations (geometric mean) were observed following intravenous (upper panel) and oral (lower panel) administration of a single dose of 400 mg moxifloxacin:

Tissue	Concentration (i.v.)	Site: Plasma ratio (i.v.)
Plasma	4.1 mg/l	-
Saliva	5.0 mg/l	0.82 - 1.37
Blister fluid	1.75[1] mg/l	1.7[1]
Interstitial fluid	1.0[2] mg/l	0.8 - 2.5[2,3]
**Tissue**	**Concentration (p.o.)**	**Site: Plasma ratio (p.o.)**
Plasma	3.1 mg/l	-
Saliva	3.6 mg/l	0.75 - 1.3
Blister fluid	1.6[1] mg/l	1.7[1]
Bronchial mucosa	5.4 mg/kg	1.7 - 2.1
Alveolar Macrophages	56.7 mg/kg	18.6 - 70.0
Epithelial lining fluid	20.7 mg/l	5 - 7
Interstitial fluid	1.0[2] mg/l	0.8 - 1.4[2,3]

[1] 10 h after administration
[2] unbound concentration
[3] from 3 h up to 36 h post dose

Metabolism

Moxifloxacin undergoes Phase II biotransformation and is excreted via renal (approximately 40%) and biliary/faecal (approximately 60%) pathways as unchanged drug as well as in the form of a sulpho-compound (M1) and a glucuronide (M2). M1 and M2 are the only metabolites relevant in humans, both are microbiologically inactive.

In clinical Phase I and *in vitro* studies no metabolic pharmacokinetic interactions with other drugs undergoing Phase I biotransformation involving cytochrome P450 enzymes were observed. There is no indication of oxidative metabolism.

Elimination

Moxifloxacin is eliminated from plasma with a mean terminal half life of approximately 12 hours. The mean apparent total body clearance following a 400 mg dose ranges from 179 to 246 ml/min. Renal clearance amounted to about 24 - 53 ml/min suggesting partial tubular reabsorption of the drug from the kidneys.

After administration of 400 mg of moxifloxacin, the recovery of the dose (unchanged drug and metabolites) totalled approximately 96 - 98%, independent of the route of administration. Following a 400 mg intravenous infusion recovery of unchanged drug from urine was approximately 22% and from faeces approximately 26%. After a 400 mg oral dose, recovery of unchanged drug from urine was approximately 19% and from faeces approximately 25%.

Concomitant administration of moxifloxacin with ranitidine or probenecid did not alter renal clearance of the parent drug.

The pharmacokinetic properties of moxifloxacin are not significantly different in patients with renal impairment (including creatinine clearance > 20 ml/min/1.73 m^2). As renal function decreases, concentrations of the M2 metabolite (glucuronide) increase by up to a factor of 2.5 (with a creatinine clearance of < 30 ml/min/1.73 m^2).

On the basis of the pharmacokinetic studies carried out so far in patients with liver failure (Child Pugh A, B), it is not possible to determine whether there are any differences compared with

healthy volunteers. Impaired liver function was associated with higher exposure to M1 in plasma, whereas exposure to parent drug was comparable to exposure in healthy volunteers. There is insufficient experience in the clinical use of moxifloxacin in patients with impaired liver function.

5.3 Preclinical safety data

Effects on the haematopoetic system (slight decreases in the number of erythrocytes and platelets) were seen in rats and monkeys. As with other quinolones, hepatotoxicity (elevated liver enzymes and vacuolar degeneration) was seen in rats, monkeys and dogs. In monkeys CNS toxicity (convulsions) occurred. These effects were seen only after treatment with high doses of moxifloxacin or after prolonged treatment.

After intravenous administration findings indicative of systemic toxicity were limited to effects on the CNS (monkeys 80 mg/kg 50 min. infusion: hypoactivity, spastic movements, salivation). The effects were most pronounced when moxifloxacin was given by bolus injection (45 mg/kg) but they were not observed when moxifloxacin (40 mg/kg) was given as slow infusion over 50 minutes.

Moxifloxacin, like other quinolones, was genotoxic in *in vitro* tests using bacteria or mammalian cells. Since these effects can be explained by an interaction with the gyrase in bacteria and - at higher concentrations - by an interaction with the topoisomerase II in mammalian cells, a threshold concentration for genotoxicity can be assumed. In *in vivo* tests, no evidence of genotoxicity was found despite the fact that very high moxifloxacin doses were used. Thus, a sufficient margin of safety to the therapeutic dose in man can be provided. Moxifloxacin was non-carcinogenic in an initiation-promotion study in rats.

Many quinolones are photoreactive and can induce phototoxic, photomutagenic and photocarcinogenic effects. In contrast, moxifloxacin was proven to be devoid of phototoxic and photogenotoxic properties when tested in a comprehensive programme of *in vitro* and *in vivo* studies. Under the same conditions other quinolones induced effects.

At high concentrations, moxifloxacin is an inhibitor of the rapid component of the delayed rectifier potassium current of the heart and may thus cause prolongations of the QT interval. Toxicological studies performed in dogs using oral doses of ≥ 90 mg/kg leading to plasma concentrations ≥ 16 mg/l caused QT prolongations, but no arrhythmias. Only after very high cumulative intravenous administration of more than 50fold the human dose (> 300 mg/kg), leading to plasma concentrations of ≥ 200 mg/l (more than 30fold the therapeutic level after intravenous administration), reversible, non-fatal ventricular arrhythmias were seen. After intravenous administration of moxifloxacin to dogs (30 mg/kg infused over 15, 30 or 60 minutes) the degree of QT prolongation was clearly depending on the infusion rate, i.e. the shorter the infusion time the more pronounced the prolongation of the QT interval. No prolongation of the QT interval was seen when a dose of 30 mg/kg was infused over 60 minutes.

Quinolones are known to cause lesions in the cartilage of the major diarthrodial joints in immature animals. The lowest oral dose of moxifloxacin causing joint toxicity in juvenile dogs was four times the maximum recommended therapeutic dose of 400 mg (assuming a 50 kg bodyweight) on a mg/kg basis, with plasma concentrations two to three times higher than those at the maximum therapeutic dose.

Toxicity tests in rats and monkeys (repeated dosing up to six months) revealed no indication regarding an oculotoxic risk. In dogs, high oral doses (≥ 60 mg/kg) leading to plasma concentrations ≥ 20 mg/l caused changes in the electroretinogram and in isolated cases an atrophy of the retina.

Reproductive studies performed in rats, rabbits and monkeys indicate that placental transfer of moxifloxacin occurs. Studies in rats (p.o. and i.v.) and monkeys (p.o.) did not show evidence of teratogenicity or impairment of fertility following administration of moxifloxacin. A slightly increased incidence of vertebral and rib malformations was observed in foetuses of rabbits but only at a dose (20 mg/kg i.v.) which was associated with severe maternal toxicity. There was an increase in the incidence of abortions in monkeys and rabbits at human therapeutic plasma concentrations. In rats, decreased foetal weights, an increased prenatal loss, a slightly increased duration of pregnancy and an increased spontaneous activity of some male and female offspring was observed at doses which were 63 times the maximum recommended dose on a mg/kg basis with plasma concentrations in the range of the human therapeutic dose.

In a local tolerability study performed in dogs, no signs of local intolerability were seen when moxifloxacin was administered intravenously. After intra-arterial injection inflammatory changes involving the peri-arterial soft tissue were observed suggesting that intra-arterial administration of moxifloxacin should be avoided.

6. PHARMACEUTICAL PARTICULARS

6.1 List of excipients

Sodium chloride
Hydrochloric acid
Sodium hydroxide
Water for injections

6.2 Incompatibilities

The following solutions were incompatible with moxifloxacin solution for infusion:
Sodium chloride 10% and 20% solutions
Sodium bicarbonate 4.2% and 8.4% solutions

6.3 Shelf life

Glass bottle: 5 years
Use immediately after first opening.

6.4 Special precautions for storage

Do not refrigerate or freeze. At cool storage temperatures precipitation may occur, which will re-dissolve at room temperature.
Store in the original container.

6.5 Nature and contents of container

Glass bottles (type 2) with a rubber stopper as closure. The 250 ml bottle is packaged in a carton. The product is available in packs of 1 and 5 bottles.

Not all pack sizes may be marketed.

6.6 Special precautions for disposal and other handling

This product is for single use only. Any unused solution should be discarded.
The following co-infusions were found to be compatible with moxifloxacin 400 mg solution for infusion:
Water for injections, Sodium chloride 0.9%, Sodium chloride 1 molar, Glucose 5%/10%/40%, Xylitol 20%, Ringer's solution, Compound Sodium Lactate Solution (Hartmann's Solution, Ringer-Lactate Solution).
Moxifloxacin solution for infusion should not be co-infused with other drugs.
Do not use if there are any visible particulate matter or if the solution is cloudy.
At cool storage temperatures precipitation may occur, which will re-dissolve at room temperature. It is therefore recommended not to store the infusion solution in a refrigerator.

7. MARKETING AUTHORISATION HOLDER

Bayer plc, Bayer House, Strawberry Hill,
Newbury, Berkshire, RG14 1JA, United Kingdom

8. MARKETING AUTHORISATION NUMBER

PA 21/48/3

9. DATE OF FIRST AUTHORISATION / RENEWAL OF THE AUTHORISATION

30.11.2003

10. DATE OF REVISION OF THE TEXT

26.07.2006
LEGAL CATEGORY

S1A

4.8 The Declaration of Helsinki

A. INTRODUCTION

1. The World Medical Association has developed the Declaration of Helsinki as a statement of ethical principles to provide guidance to physicians and other participants in medical research involving human subjects. Medical research involving human subjects includes research on identifiable human material or identifiable data.

2. It is the duty of the physician to promote and safeguard the health of the people. The physician's knowledge and conscience are dedicated to the fulfillment of this duty.

3. The Declaration of Geneva of the World Medical Association binds the physician with the words, "The health of my patient will be my first consideration," and the International Code of Medical Ethics declares that, "A physician shall act only in the patient's interest when providing medical care which might have the effect of weakening the physical and mental condition of the patient."

4. Medical progress is based on research which ultimately must rest in part on experimentation involving human subjects.

5. In medical research on human subjects, considerations related to the well-being of the human subject should take precedence over the interests of science and society.

6. The primary purpose of medical research involving human subjects is to improve prophylactic, diagnostic and therapeutic procedures and the ANDerstanding of the aetiology and pathogenesis of disease. Even the best proven prophylactic, diagnostic, and therapeutic methods must continuously be challenged through research for their effectiveness, efficiency, accessibility and quality.

7. In current medical practice and in medical research, most prophylactic, diagnostic and therapeutic procedures involve risks and burdens.

8. Medical research is subject to ethical standards that promote respect for all human beings and protect their health and rights. Some research populations are vulnerable and need special protection. The particular needs of the economically and medically disadvantaged must be recognized. Special attention is also required for those who cannot give or refuse consent for themselves, for those who may be subject to giving consent under duress, for those who will not benefit personally from the research and for those for whom the research is combined with care.
9. Research Investigators should be aware of the ethical, legal and regulatory requirements for research on human subjects in their own countries as well as applicable international requirements. No national ethical, legal or regulatory requirement should be allowed to reduce or eliminate any of the protections for human subjects set forth in this Declaration.

B. BASIC PRINCIPLES FOR ALL MEDICAL RESEARCH

1. It is the duty of the physician in medical research to protect the life, health, privacy, and dignity of the human subject.

2. Medical research involving human subjects must conform to generally accepted scientific principles, be based on a thorough knowledge of the scientific literature, other relevant sources of information, and on adequate laboratory and, where appropriate, animal experimentation.

3. Appropriate caution must be exercised in the conduct of research which may affect the environment, and the welfare of animals used for research must be respected.

4. The design and performance of each experimental procedure involving human subjects should be clearly formulated in an experimental protocol. This protocol should be submitted for consideration, comment, guidance, and where appropriate, approval to a specially appointed ethical review committee, which must be independent of the investigator, the sponsor or any other kind of undue influence. This independent committee should be in conformity with the laws and regulations of the country in which the research experiment is performed. The committee has the right to monitor ongoing trials. The researcher has the obligation to provide monitoring information to the committee, especially any serious adverse events. The researcher should also submit to the committee, for review, information regarding Funding, sponsors, institutional affiliations, other potential conflicts of interest and incentives for subjects.

5. The research protocol should always contain a statement of the ethical considerations involved and should indicate that there is compliance with the principles enunciated in this Declaration.

6. Medical research involving human subjects should be conducted only by scientifically qualified persons and under the supervision of a clinically competent medical person. The responsibility for the human subject must always rest with a medically qualified person and never rest on the subject of the research, even though the subject has given consent.

7. Every medical research project involving human subjects should be preceded by careful assessment of predictable risks and burdens in comparison with foreseeable benefits to the subject or to others. This does not preclude the participation of healthy volunteers in medical research. The design of all studies should be publicly available.

8. Physicians should abstain from engaging in research projects involving human subjects unless they are confident that the risks involved have been adequately assessed and can be satisfactorily managed. Physicians should cease any investigation if the risks are found to outweigh the potential benefits or if there is conclusive proof of positive and beneficial results.

9. Medical research involving human subjects should only be conducted if the importance of the objective outweighs the inherent risks and burdens to the subject. This is especially important when the human subjects are healthy volunteers.

10. Medical research is only justified if there is a reasonable likelihood that the populations in which the research is carried out stand to benefit from the results of the research.

11. The subjects must be volunteers and informed participants in the research project.

12. The right of research subjects to safeguard their integrity must always be respected. Every precaution should be taken to respect the privacy of the subject, the confidentiality of the patient's information and to minimize the impact of the trial on the subject's physical and mental integrity and on the personality of the subject.

13. In any research on human beings, each potential subject must be adequately informed of the aims, methods, sources of Funding, any possible conflicts of interest, institutional affiliations of the researcher, the anticipated benefits and potential risks of the trial and the discomfort it may entail. The subject should be informed of the right to abstain from participation in the trial or to withdraw consent to participate at any time without reprisal. After ensuring that the subject has understood the information, the physician should then obtain the subject's freely-given informed consent, preferably in writing. If the consent cannot be obtained in writing, the non-written consent must be formally documented and witnessed.

14. When obtaining informed consent for the research project the physician should be particularly cautious if the subject is in a dependent relationship with the physician or may consent under duress. In that case the informed consent should be obtained by a well-informed physician who is not engaged in the investigation and who is completely independent of this relationship.

15. For a research subject who is legally incompetent, physically or mentally incapable of giving consent or is a legally incompetent minor, the investigator must obtain informed

consent from the legally authorized representative in accordance with applicable law. These groups should not be included in research unless the research is necessary to promote the health of the population represented and this research cannot instead be performed on legally competent persons.

16. When a subject deemed legally incompetent, such as a minor child, is able to give assent to decisions about participation in research, the investigator must obtain that assent in addition to the consent of the legally authorized representative.

17. Research on individuals from whom it is not possible to obtain consent, including proxy or advance consent, should be done only if the physical/mental condition that prevents obtaining informed consent is a necessary characteristic of the research population. The specific reasons for involving research subjects with a condition that renders them unable to give informed consent should be stated in the experimental protocol for consideration and approval of the review committee. The protocol should state that consent to remain in the research should be obtained as soon as possible from the individual or a legally authorized surrogate.

18. Both authors and publishers have ethical obligations. In publication of the results of research, the investigators are obliged to preserve the accuracy of the results. Negative as well as positive results should be published or otherwise publicly available. Sources of Funding, institutional affiliations and any possible conflicts of interest should be declared in the publication. Reports of experimentation not in accordance with the principles laid down in this Declaration should not be accepted for publication.

J. ADDITIONAL PRINCIPLES FOR MEDICAL RESEARCH COMBINED WITH MEDICAL CARE

1. The physician may combine medical research with medical care, only to the extent that the research is justified by its potential prophylactic, diagnostic or therapeutic value. When medical research is combined with medical care, additional standards apply to protect the patients who are research subjects.
2. The benefits, risks, burdens and effectiveness of a new method should be tested against those of the best current prophylactic, diagnostic, and therapeutic methods. This does not exclude the use of placebo, or no treatment, in studies where no proven prophylactic, diagnostic or therapeutic method exists. See footnote

3. At the conclusion of the trial, every patient entered into the trial should be assured of access to the best proven prophylactic, diagnostic and therapeutic methods identified by the trial. See footnote

4. The physician should fully inform the patient which aspects of the care are related to the research. The refusal of a patient to participate in a trial must never interfere with the patient-physician relationship.

5. In the treatment of a patient, where proven prophylactic, diagnostic and therapeutic methods do not exist or have been ineffective, the physician, with informed consent from the patient, must be free to use unproven or new prophylactic, diagnostic and therapeutic measures, if in the physician's judgement it offers hope of saving life, re-establishing health or alleviating suffering. Where possible, these measures should be made the object of research, designed to evaluate their safety and efficacy. In all cases, new information should be recorded and, where appropriate, published. The other relevant guidelines of this Declaration should be followed.

Note: **Note of clarification on paragraph 29 of the WMA Declaration of Helsinki**

The WMA hereby reaffirms its position that extreme care must be taken in making use of a placebo-controlled trial and that in general this methodology should only be used in the absence of existing proven therapy. However, a placebo-controlled trial may be ethically acceptable, even if proven therapy is available, under the following circumstances:

- Where for compelling and scientifically sound methodological reasons its use is necessary to determine the efficacy or safety of a prophylactic, diagnostic or therapeutic method; or

- Where a prophylactic, diagnostic or therapeutic method is being investigated for a minor condition and the patients who receive placebo will not be subject to any additional risk of serious or irreversible harm.

All other provisions of the Declaration of Helsinki must be adhered to, especially the need for appropriate ethical and scientific review.

Page back to paragraph 29.

Note: **Note of clarification on paragraph 30 of the WMA Declaration of Helsinki**

The WMA hereby reaffirms its position that it is necessary during the trial planning process to identify post-trial access by trial participants to prophylactic, diagnostic and therapeutic procedures identified as beneficial in the trial or access to other appropriate care. Post-trial access arrangements or other care must be described in the trial protocol so the ethical review committee may consider such arrangements during its review.

Page back to paragraph 30.

The Declaration of Helsinki (Document 17.C) is an official policy document of the World Medical Association, the global representative body for physicians. It was first adopted in 1964 (Helsinki, Finland) and revised in 1975 (Tokyo, Japan), 1983 (Venice, Italy), 1989 (Hong Kong), 1996 (Somerset-West, South Africa) and 2000 (Edinburgh, Scotland). Note of clarification on Paragraph 29 added by the WMA General Assembly, Washington 2002.

9.10.2004

4.9 Medicinal Products Act (Extract)

The Protection of Human Subjects in Clinical Trials

§ 40

General Conditions for the Clinical Trial

(1) The clinical trial of a medicinal product may only be conducted on human beings if and as long as:

1. The foreseeable risks are medically justifiable in relation to the expected benefits for the persons upon whom the trial shall be conducted and in consideration of the anticipated significance of the medicinal product for medical science.

2. The person on whom the trial shall be conducted, after being informed by a physician about the nature, significance and implications of the clinical trial, has consented to the trial and has declared that this consent also relates to the recording and Communication of health data to the sponsor, to the competent supervisory authority or Federal Agency and, in so far as it concerns person-related data that these may be viewed by persons authorised by the sponsor or by the authorities.

3. The person on whom the trial shall be conducted has not been committed to an institution by a court order or by order of the authorities,

4. the trial is conducted by an investigating physician who can provide evidence of at least two years of experience in the clinical trial of medicinal products.

5. a pharmacological-toxicological test of the medicinal product according to the prevailing state of scientific knowledge has been carried out.

6. the documents pertaining to the pharmacological-toxicological trial, the trial flow-chart in accordance with the prevailing state of medical knowledge, giving the names of the investigating physicians and the location of the trials and the vote of the Ethics Committee responsible for the management of the clinical trial have been submitted to the competent Federal authority.

7. The management of the clinical trial has been informed by a research scientist responsible for the pharmacological-toxicological test concerning the results of the pharmacological-toxicological test and the risks anticipated in connection with the clinical trial.

8. In the event that a person is killed or a person's body or health is injured during the course of the clinical trial, an insurance policy must exist, in accordance with the provisions stipulated under Section 3, which provides benefits even when no other third party is liable for damages.

The clinical trial of a medicinal product may only be conducted, subject to Sentence 3, if the trial has been positively assessed by an Ethics Commttee formed in compliance with the applicable laws of the Land; prerequisite to this approval is the adherence to the provisions stipulated under Sentence 1 Numbers 1 - 5, and Number 6, as far as pertaining to documents concerning the pharmacological-toxicological test and the trial flow-chart, as well as 7 and 8. In so far as no positive assessment from the Ethics Committee is available, the clinical trial may not be initiated unless the competent Federal authority has not objected within 60 days after receipt of the documents according to Sentence 1 Number 6. The Ethics Committee must be informed of all serious or unexpected, undesired events that occur during the trial and which may have an adverse effect on the safety of the trial participants or the execution of the trial

(2) A declaration of consent, according to Section 1 No. 2, is only effective if the person submitting the declaration

1. is legally competent and in a position to comprehend the nature, significance and implications of the clinical trial and to make a decision on this basis,

2. has granted his or her consent personally in writing.

Consent may be revoked at any time.

The insurance, in compliance with subsection 1 No. 8, must be taken out in favour of the person concerned in the clinical trial with an insurance provider authorised to conduct business in the area of validity of this law. The insurance cover must be in a commensurate relationship to the risks involved in the clinical trial and, in the event of the death or permanent inability to work of the person concerned, must amount to at least the equivalent of one million deutsche mark. Insofar as indemnification is paid by the insurance, further claims to compensation are extinguished.

(4) In respect of clinical trials on minors, subsection 1 to 3 shall apply with the following proviso:

1. The medicinal product must be intended to diagnose or prevent diseases in minors.

2. The use of the medicinal product must be indicated in accordance with medical scientific knowledge for the purpose of diagnosing or preventing disease in the minor.

3. According to medical, scientific knowledge, clinical trials performed on adults may not be expected to produce satisfactory test results.

4. The consent shall be granted by the legal representative. It is only effective if the legal representative has been fully informed by the attending physician concerning the nature, significance and implications of the clinical trial. If the minor is in a position to comprehend the nature, significance and implications of the clinical trial and to make a decision on this basis, a written declaration of consent is required.

(5) the Federal Ministry is empowered by statutory ordinance with the consent of the German Upper House (Bundesrat) to impose regulations to ensure the proper execution of the clinical trial and to obtain corresponding documents in accordance with the state of scientific medical knowledge. The ordinance may contain more detailed stipulations in regard to the tasks and the fields of responsibility of the persons initiating or controlling the clinical trial and requirements concerning the keeping and storage of documentary records. Furthermore, the ordinance may contain the granting of authorisation for the recording, processing and use of person-related data, in so far as these are required for the monitoring of the clinical trial. This also applies to the processing of data which are not processed or used in digital files.

UNIVERSITY CLINIC - MEDICAL FACULTY OF THE HUMBOLDT UNIVERSITY - BERLIN

Clinic and Polyclinic for Neurology
Director: Prof. Dr. K. M. Einhäupl

Campus Charité Mitte
Schumannstrasse 20/21
10098 Berlin
Tel.: 030-45056102
Fax 030-450560032

16/07/2002

Information Sheet for Patients **16/07/2002**

Information Sheet for Patients 16/07/2002

concerning scientific medical trial:

"Pilot study on preventive antibacterial short-term therapy

for patients with acute MCA territory, ischemic infarction"

at the Neurological Clinic of the Charité University

of the Humboldt University, Berlin

Dear Patient,

You have suffered a stroke and we, as physicians, will do everything in our power to help you in recovering from this stroke and to overcome its consequences.

Of course, we are constantly working on improving our treatment methods. Innovations in the therapy which give us reason to hope for an improvement for our patients, however, must always be subjected to precise scientific tests while maintaining high safety standards.

We have requested you to participate voluntarily in such a scientific medical trial.

Already, in a personal consultation with you, we have explained why we regard this scientific trial and your participation as important and what it would mean for you if you were to declare your willingness to take part.

To make it easier for you to decide on this matter, we should like to present to you once more in writing the aims, background information and procedures as well as explain questions in regard to your safety and the risks involved in the trial.

The Aims of the Study

Patients who, like yourself, have suffered a larger stroke, unfortunately very often fall ill in the first few days after the stroke with additional infections, such as pneumonia or even blood poisoning (sepsis). Such complications not only hamper the healing process, but they can often be life-threatening as well.

Of course, every patient with such infections immediately receives intensive treatment with antibiotics as soon as the infections have been detected.

On the basis of new research findings, we now have reason to suppose that patients, such as yourself, could benefit from receiving treatment with antibiotics even before the onset of complicating infections, in other words, looking ahead, a treatment that would be conducted for a few days in advance.

Should it be possible, with the help of this treatment, to reduce the frequency of serious infections, this might also bring with it better prospects of a successful healing process after a stroke.

However, so far, all this is only supposition on the basis of animal experiments and has not yet been scientifically tested. First, before it can be applied as a matter of routine, it must be proved in a scientific trial whether a prophylactic treatment of this nature really leads to noticeably better results for the patients .

We are conducting this study

1. in order to compare the results of the therapy used so far with those of the new therapy approach: the treatment principle applied so far,

according to which infections are treated as quickly as possible after they break out, and the new treatment principle, in which stroke patients, like yourself, shall already receive antibiotic treatment prophylactically in order to prevent serious infections.

2. in order to describe more precisely with the help of microbiological and immunological tests the risk of infection on the part of stroke patients and to better understand its causes.

Who is conducting the study?

The scientific medical planning and execution of this study is being conducted by investigating physicians of the Neurological Clinic of the Charité.

For the execution of our study, we are being sponsored by the German Research Foundation and by the German company Bayer Vital Ltd, which is also providing the antibiotic Moxifloxacin, which we shall be using in the course of the study.

How is it planned to conduct the study?

We shall try to win over *all* of our patients with serious strokes to take part in the study.

Once the patient has declared himself willing to participate, he or she will be allotted at random to one of the two study groups:

A) The one group will be treated with an antibiotic, as normally, once an infection has already broken out. The treatment of these patients corresponds, therefore, to the normal modern standard.

B) The other group of patients shall receive, as early as possible after the stroke, a preventive five-day treatment with the antibiotic *Moxifloxacin*.

Because most, but not all, stroke patients suffer complications caused by infection, this means that some of the patients of this group will receive the

antibiotic without really needing it. Hence, not the infection, but already the risk of infection shall be treated. This is currently not the standard treatment.

In order to prevent prejudices on the part of the physicians and patients during the execution and assessment of the study, it is important that **neither the patient nor the physician knows which patient belongs to which study group**. If, at any time, there is the least suspicion that the safety of a patient seems to be endangered, the physician is able to obtain this information immediately without difficulty.

Now, so as to avoid that the two patient groups can be outwardly distinguished from one another, the patients in Group A, who at the beginning are not to receive any antibiotics, are given an ineffective saline solution, which is packed to look exactly like the antibiotic.

Both the prophylactic antibiotic as well as the saline solution are administered in the form of an infusion.

Physical Examinations

All participants in the study remain under our hospital care during the acute treatment phase. During this time, they are subjected to regular examinations and health monitoring.

Besides the regular examination of the lungs and heart, the functions of the brain, e.g. speech, strength, coordination capacity and sensitivity to the touch are monitored.

Ex-ray and MRT Examinations

It is planned to conduct x-ray examinations of the lung in the course of the study.

In addition, over a period of six months, examinations of the head using Magnetic Resonance Tomography (MRT) shall be conducted.

Blood and Urine Tests

During the course of the study, blood samples will be taken from the participants on ten days. These tests are conducted in the normal course of routine sampling.

Urine samples will also be collected and examined.

<u>Throat and Rectal Smears</u>

In order to avoid that the new treatment method may lead, in the long term, to undesired changes in the viruses (the formation of resistance), throat and rectal smears will be taken before the beginning and after the end of the treatment (9th day). These samples will then be examined by the Institute for Microbiology and Hygiene.

How long does the study take?

The participants in the study will receive direct hospital care during the entire acute phase of the stroke conducted by physicians of the Neurological Clinic. As soon as a stable state of health has been reached, the patients will be transferred to a rehabilitation establishment. Subsequently, three and six months after the stroke, we shall be conducting follow-up examinations.

The examination programme in the follow-up phase includes a medical consultation and the physical examinations, each comprising an MRT of the head and a blood test.

Does the study modify your treatment?

The essential differences from the routine stroke treatment at out clinic, for the study participants, consist in the longer follow-up phase and the administering of the study medicament, i.e. a saline solution for the one group of patients and an antibiotic for the other.

Over and above this, the normal medical procedure in the treatment of a stroke is essentially not influenced.

Most of the examinations necessary for the study are also conducted as a matter of routine for other stroke patients.

The taking of blood samples is hardly any more frequent than in the case of other stroke patients.

Are complications to be expected as a result of the examinations conducted during the course of the study?

All of the examination methods presented here involve little risk and are also used during routine treatment.

The x-ray examinations involve a certain radiation exposure. However, these exposure levels are approximately the same as those for patients who are not taking part in the study.

The MRT examinations are performed using magnetic fields and radio waves and do not constitute any radiation exposure. MRT examinations have been conducted for many years without negative health effects.

For patients with pace-makers or similar medical appliances and in the case of metallic parts in the body (screws, shell splinters, prostheses) this examination method, however, cannot be used.

After the taking of blood samples, it may come to small bruises or painful swellings at the pricking point, but rarely to nerve or vein irritations. These fade away of their own accord.

The amount taken is so small that no negative effect for the patient can be expected. Moreover, the body continually replenishes the amount of blood.

Which medicament will be used?

What side-effects does it have?

Can it damage your health?

For the antibacterial, short-term therapy applied in this study, we shall be using the antibiotic *Moxifloxacin* produced by the German company Bayer Vital.

Moxifloxacin is an antibiotic which is used primarily for the treatment of infections of the respiraratory tracts, bacterial pneumonia and supperative, paranasal sinusitis.

Like all medicinal products, apart from the desired effects, *Moxifloxacin* also has undesired side-effects. However, it is a safe drug and has been examined by the competent authorities of the Federal Republic of Germany and has been approved for the treatment of infections.

The most frequent side-effects are **nausea, diarrhoea and vomiting**, which may occur in every tenth patient.

Furthermore, it is known that *Moxifloxacin* may tend to favour certain kinds of cardiac arrhythmia. However, we have not been able to detect any sign that you have such a tendency. To be on the safe side, however, we shall monitor your heart reaction during the entire period of treatment with this medicament. Should, contrary to expectation, dangerous cardiac disturbances occur, we shall immediately discontinue your participation in the study. As a matter of course, you will at all times receive treatment in accordance with state-of-the-art medical practice.

Since *Moxifloxacin*, like many medicaments, exerts strain on the **liver metabolism**, we shall monitor any possible effects this medicament may have on you in regard to your liver by means of regular blood tests.

If you have ever experienced an **allergic reaction** to an antibiotic, we must ascertain whether it was caused by a medicament similar to *Moxifloxacin*. Should this be the case, you must under no circumstances participate in the study. Other allergies, also against penicillin, on the other hand, are of no consequence.

Although *Moxifloxacin* is a safe drug, which only very rarely gives rise to serious complications, in case of doubt, we shall always interrupt the study

at the first sign of any disquieting symptoms, in order to avoid any unnecessary risk for the study participants.

INSURANCE COVER

In the event that, notwithstanding all efforts and all precautionary measures, contrary to expectation, damage to your health should occur as a result of your participation in the study, it is important for you to know that, during the course of the study, you enjoy the following insurance cover:

Under an insurance policy, taken out with Gerling Industrie-Service GmbH West, Prinzenallee 21, 40549 Düsseldorf, Telephone 0211/49560, policy number 70-5560355, for health damage resulting from participation in the study, the study participants are insured to the amount of 500,000 euros.

To ensure that this insurance cover is valid, you must observe the following points:

1. during the period of the clinical trial, except in an emergency, you may not undergo medical treatment provided by other physicians without the consent of the investigating physician.
2. In the event that, contrary to expectation, health damage should occur which could be a result of the clinical trial, this must be reported immediately to the investigating physician and to the insurance company.

What happens to your data?

Should you participate in this study, your person-related data recorded in the course of the study, health data, illness records, as well as the findings of the tests carried out during the study, together with your treatment data will be processed in the following manner:

Your **personal data** (name, Christian name, sex, date of birth and address) will be recorded by the physician who supplies you with information about the study and will be noted on your declaration of consent. This declaration of consent, together with the uncoded person-related data, will remain in the custody of the investigating physician. This data will be treated in strict confidence.

The **data concerning your health and illnesses** obtained in the course of the study, as well as the findings of the study, will be **encoded** (pseudonymised) and stored electronically in this form. The encryption code, which is used to be able to relate these data to your personal data, is stored separately from the other data on a different computer and is only accessible to the investigating physician.

The **anonymous findings of the study** are likely to be published in a medical journal, i.e. without mentioning your name or other data which might reveal your identity.

The data concerning your health and/or illness recorded and encoded during the course of this study may be sent, or electronically transmitted, to the sponsor of this study, the company BayerVital, as well as to the competent supervisory authorities in Germany or abroad and to the Supreme Federal Authority for the purposes of this study.

Furthermore, representatives of the aforesaid agencies, who are bound to secrecy, may receive, in individual cases, insight into the records of the investigating physician pertaining to your participation in the study, from which your name, address as well as your full date of birth can be seen. This is to ensure, in particular, that the data transmitted by the investigating physician is correct.

The stored or otherwise recorded data / person-related information and the encryption code will be stored and/or archived for a period of 15 years and then erased or destroyed in accordance with the legal regulations on the compulsory storage of data. The recorded data pertaining to the above mentioned study possibly contained in the patient's case record will be stored for a period of 30 years and then destroyed.

At any time, you are entitled to object to the further processing of your data. In this case, the personal data and corresponding code relating to your person will be erased and/or destroyed, in so far as this does not contravene legal or professional regulations in respect of the compulsory storage of data.

At any time, you may request the correction of incorrect data referring to your person.

The blood samples taken in the course of the study will also be encoded (pseudonymised), so that a relationship between your person and the blood sample can only be established together with the encryption code stored on a separate computer by the investigating physician.

At your request, we shall, naturally, inform you concerning the findings of the tests performed and the content of any other information recorded in relation to your person.

If you any questions.

Should you have any questions in regard to the study or to the examinations in the course of the study, but also any other questions, the investigating physician is at your disposal at any time and will be happy to answer them for you.

The investigating physician responsible for you is:

....

Telephone:

....

We should also like to point out that you, as study participant, are obliged to follow the instructions of the investigating physician during your participation in the trial and to inform him immediately of any changes in your state of health or well-being. The same applies to medical measures and other circumstances not relating to the study which could have an influence on the course of the clinical study and/or your participation.

Can you refuse to participate?

Can you withdraw your consent?

Naturally, you have the inviolable right to refuse participation in the study.

It is also quite normal if you make use of this right. You are under absolutely no obligation!

Moreover, also **after** having given your consent, you may, **at any time** and **without giving any reasons,** withdraw your consent to participation in the study.

Whichever way you decide, your health is our utmost concern and we shall conduct your treatment, in any case, in accordance with the state-of-the-art of medical practice.

The same applies in the case of your possible objection to the further processing of your data.

Your participation in the trial, however, can also be discontinued by the investigating physician should he deem this necessary for safety reasons, due to changes in the procedure for executing the study or for other reasons.

Your Questions and Comments

I have read the patients information regarding the "Pilot study on the preventive antibacterial therapy of patients with acute MCA territory ischemic infarction"

With my signature, I hereby confirm that I have received the study information.

Berlin, dated ………………………………

....

Signature of the study participant

....

Signature of the legal representative, as applicable

Declaration of Consent

Declaration of Consent

concerning participation in the

Pilot Study on Preventive Antibacterial Short-term Therapy on Patients with Acute, MCA Territory Ischemic Infarction

I hereby declare

....
(name and Christian name of the patient)

....
(resident at)

....
(born on …)

that I have been informed by

....
(name of investigating physician)

verbally and in writing, concerning the nature significance and implications as well as the risks of the scientific trial in the course of the " pilot study on the preventive antibacterial short-term therapy on patients with acute MCA territory ischemic infarction", which is to be carried out by the Neurological Clinic of the Charité, and have received sufficient opportunity to clarify my questions in this regard in consultation with the investigating physician.

In particular, I have read and understood the patient information sheet, dated …………., a copy of which I have received together with a copy of this declaration of consent.

I declare my willingness to participate in the scientific trial in the course of the above-mentioned study.

I am aware that I may withdraw my consent at any time without giving any reasons and without any disadvantageous consequences for me and that, at any time, I may also object to the further processing of my data.

I have been informed about the existing insurance cover and my obligations in this connection.

I am in agreement that the chief investigating physician or investigating physician may contact my attending physician during the course of this study.

Declaration of Consent to the Processing of Data

I hereby grant my consent that the Neurological Clinic may process data relating to my person, health and/or illness data in the framework and for the purposes of the above-mentioned research project.

I am in agreement that my illness data may be recorded in the course of the above-mentioned study, that it may be encrypted (pseudonymised and/or anonymised), stored in encrypted form (pseudonymised and/or anonymised) and that it may be transmitted to the supervisory authorities and may be published in anonymised form.

Furthermore, I am in agreement that an authorised representative of the competent supervisory authority, who is bound to secrecy, and the Supreme

Federal Authority may be granted random insight into the illness data recorded in the course of the study for investigative purposes in so far as such data is related to my person.

I likewise declare my agreement that my family doctor may be informed by the investigating physicians concerning my participation in the above-mentioned study.

In the course of the transmission of data and the granting of random insight into records pertaining to my person, as described above, I hereby release my attending physicians and investigating physicians from their legal obligation to maintain professional secrecy.

Moreover, I am in agreement with the taking, removal, examination and storage of encrypted (pseudonyminised and/or anonymised) blood samples and/or tissue and the taking therefrom of genetic materials in the course of the study for the purposes of this clinical trial by the investigating physicians and/or the scientists involved in the study.

Berlin, dated ……………………………

....
Signature of the study participant

Berlin, dated ……………………………

....
Signature of the legal representative or witnesses, as applicable

I hereby declare that I have informed the above named study participant on (date)… concerning the nature, significance, implications and risks of the above-mentioned study both verbally and in writing and that I have handed out to the participant a copy of this information sheet as well as a copy of the declaration of consent.

Berlin, dated ……………………………

....

Signature of the investigating physician responsible for patient information
